# The presence of genetic risk variants within PTPN2 and PTPN22 is associated with intestinal microbiota alterations in Swiss IBD cohort patients

**DOI:** 10.1371/journal.pone.0199664

**Published:** 2018-07-02

**Authors:** Bahtiyar Yilmaz, Marianne R. Spalinger, Luc Biedermann, Yannick Franc, Nicolas Fournier, Jean-Benoit Rossel, Pascal Juillerat, Gerhard Rogler, Andrew J. Macpherson, Michael Scharl

**Affiliations:** 1 Maurice Müller Laboratories, Department for Biomedical Research, University of Bern, Bern, Switzerland; 2 Department of Visceral Surgery and Medicine, Bern University Hospital, University of Bern, Bern, Switzerland; 3 Department of Gastroenterology and Hepatology, University Hospital Zürich, University of Zürich, Zürich, Switzerland; 4 Institute of Social and Preventive Medicine (IUMSP), Lausanne University Hospital, Lausanne, Switzerland; Laikon Hospital, GREECE

## Abstract

**Background:**

Genetic risk factors, intestinal microbiota and a dysregulated immune system contribute to the pathogenesis of inflammatory bowel disease (IBD). We have previously demonstrated that dysfunction of protein tyrosine phosphatase non-receptor type 2 (PTPN2) and PTPN22 contributes to alterations of intestinal microbiota and the onset of chronic intestinal inflammation *in vivo*. Here, we investigated the influence of PTPN2 and PTPN22 gene variants on intestinal microbiota composition in IBD patients.

**Methods:**

Bacterial DNA from mucosa-associated samples of 75 CD and 57 UC patients were sequenced using 16S rRNA sequencing approach. Microbial analysis, including alpha diversity, beta diversity and taxonomical analysis by comparing to PTPN2 (rs1893217) and PTPN22 (rs2476601) genotypes was performed in QIIME, the *phyloseq* R package and MaAsLin pipeline.

**Results:**

In PTPN2 variant UC patients, we detected an increase in relative abundance of unassigned genera from *Clostridiales* and *Lachnospiraceae* families and reduction of *Roseburia* when compared to PTPN2 wild-type (WT) patients. *Ruminoccocus* was increased in PTPN22 variant UC patients. In CD patients with severe disease course, *Faecalibacterium*, *Bilophila*, *Coprococcus*, unclassified *Erysipelotrichaeceae*, unassigned genera from *Clostridiales* and *Ruminococcaceae* families were reduced and *Bacteroides* were increased in PTPN2 WT carriers, while *Faecalibacterium*, *Bilophila*, *Coprococcus*, and *Erysipelotrichaeceae* were reduced in PTPN22 WT patients when compared to patients with mild disease. In UC patients with severe disease, relative abundance of *Lachnobacterium* was reduced in PTPN2 and PTPN22 WT patients, *Dorea* was increased in samples from PTPN22 WT carriers and an unassigned genus from *Ruminococcaceae gen*. was increased in patients with PTPN2 variant genotype.

**Conclusions:**

We identified that IBD-associated genetic risk variants, disease severity and the interaction of these factors are related to significant alterations in intestinal microbiota composition of IBD patients.

## Introduction

The gut microbiota is vital for several critical host physiological processes including digestion of dietary factors, development of the host immune system, and colonization resistance against invading pathogens[1–3]. An alteration of the gut microbial composition has been associated with the development of various intestinal and extraintestinal disorders, including inflammatory bowel disease (IBD) [4–6]. Crohn’s disease (CD) and ulcerative colitis (UC), the major IBD sub-types, represent phenotypically different diseases in terms of sites of intestinal involvement, histopathology and clinical characteristics [7, 8]. However, there are intermediate forms and genetic linkages show substantial overlaps between CD, UC and other autoimmune and inflammatory conditions. Recent studies suggest a crucial role of intestinal microbiota together with environmental factors in the genetically susceptible host consequently resulting in chronic intestinal inflammation [7, 9].

To date, more than 240 IBD risk genes (e.g. NOD2, CARD9, ATG16L1, IRGM, TNFSF15, GPR65, IL23R, IL12B and TTC7A) have been identified and many of them are involved in the regulation of host-microbiota interactions [10–12]. Among those risk genes, protein tyrosine phosphatase non-receptor type 2 (PTPN2) as well as PTPN22 are well-studied. While single nucleotide polymorphisms (SNP) in PTPN2 are a risk factor for the onset of CD as well as UC, SNP rs2476601 within the gene locus encoding PTPN22 protects from CD [13–15].

We have previously demonstrated that PTPN2 is involved in maintaining intestinal epithelial barrier function, regulating autophagosome function and immune responses to invading bacteria in intestinal cells, limiting pro-inflammatory cytokine secretion in the intestine as well as controlling differentiation and function of CD4^+^ T-cells *in vivo* [16]. Hereby, the presence of the IBD-associated PTPN2 variant promotes a pro-inflammatory phenotype [16–22]. Of particular interest, we found that the presence of the disease-associated PTPN2 variant in patients from the Swiss IBD Cohort Study (SIBDCS) is associated with a more severe disease course, in particular increased frequency of pancolitis and intestinal surgery in UC patients, however a better treatment response to anti-TNF antibodies in both CD and UC patients [23]. We also showed that mice deficient for PTPN2 in CD4^+^ T-cells compartment have altered intestinal microbiota composition with a similar trend as observed in IBD patients [16].

In the intestine of CD patients, mRNA and protein levels of PTPN22 are mostly downregulated resulting in attenuated IFN-gamma signalling [24]. Our recent data demonstrate that PTPN22 is a critical regulator of the NLRP3 inflammasome by controlling NLRP3 tyrosine phosphorylation [25]. Further, we found that PTPN22 controls NLRP3-mediated IL-1beta secretion in an autophagy dependent manner [26]. In SIBDC patients suffering from CD, the presence of the protective PTPN22 allele was associated with less use of steroids and antibiotics and reduced prevalence of vitamin D and calcium deficiency [27].

The role of the microbiota for developing and maintaining the immune system as well as in the pathogenesis of IBD has been well documented [11, 28–30]. Aberrant intestinal microbiota compositions, often referred to as intestinal dysbiosis, in UC patients is characterized by a decreased microbial diversity within the individual gut microbiota [29, 30]. In addition, UC has been described to have a distinct phylogenetic fingerprint with enrichment in putative beneficial indicator bacteria, such as *Proteobacteria* and *Fusobacterium*, and decreased abundance of protective indicator bacteria, such as *Lachnospiraceae*, *Ruminococcaceae*, *Bifidobacterium*, *Roseburia* and *Sutterella* [31, 32]. In CD patients, a depletion of beneficial bacteria, such as *Firmicutes*, *Bifidobacterium* or *Clostridia* and an increase in taxa from *Bacteroidetes* and *Enterobacteria* are the main characteristics of altered intestinal microbiota [33, 34]. In addition, it was shown that mutations in several IBD susceptibility genes lead to impaired immune responses to commensal bacteria, and subsequently to an imbalance in the taxonomic composition of the intestinal microbiota [12]. However, it has not been yet investigated whether PTPN2 and PTPN22 gene variants are associated with intestinal microbiota changes in IBD patients.

In the present study, we aimed to investigate the relevance of genetic variations in the IBD risk genes PTPN2 and PTPN22 for intestinal microbiota composition and disease course in CD and UC patients.

## Materials and methods

### Cohorts and study design

Most IBD studies have focused on fecal bacteria (non-invasive sampling method for gut microbial characterization), instead of mucosa-associated bacteria, although the mucosal microbiota is in closer proximity to intestinal immune system. Even though fecal microbiota may simply be a numerical transformation of the mucosa-associated microbiota, microbial comparisons of mucosa-associated samples and fecal samples showed that these two compartments have significantly different microbial communities [35, 36] Hence, we have decided to investigate the gut microbial profile of IBD patient at the disease site.

Patient mucosa-associated samples and data were entirely extracted from the register of the nationwide Swiss IBD cohort study (SIBDCS) which comprises more than 3500 patients from all regions of Switzerland [37]. The SIBDCS is an observational cohort study funded by the Swiss National Science Foundation for research (Grant N° 3347CO-108792/1) started in 2006 by collaboration of the five university hospitals of Switzerland and subsequently including gastroenterologists working in smaller hospitals or private practice, finally covering the whole country. Since then, at least one third of the current 3500 patients have been followed prospectively for more than 10 years (data from March 2017) [37]. We have collected 941 biopsies of 346 IBD patients for microbial characterization of these patients in the presence of PTPN2 and PTPN22 variants.

### Ethics statement

The SIBDCS has been approved by the respective ethics committees in Switzerland. The lead ethics committee is the Cantonal Ethics Committee of Zürich (KEK Zurich: EK-1316). The presented study is part of the research plan of the SIBDCS which means that no separate ethical approval was necessary for this project and the project is approved under the EK-1316 approval. Basic demographic data, clinical data and information about possible confounders of microbiota analysis such as age, gender, BMI, medication were collected at the time of inclusion for all patients. All patients signed an informed consent and confirmed their participation in the cohort study at the time of enrolment and gave informed consent for data collection and analysis for research purposes. The current sub-study has been evaluated and approved by the scientific board of SIBDC.

### DNA extraction from human biopsies

Intestinal endoscopic biopsies from different location along gut of each patient were initially collected into 2 ml microfuge tubes containing RNAlater (Sigma-Aldrich) and stored at -80°C prior to DNA extraction protocol that was carried out using AllPrep DNA/RNA Mini Kit (Qiagen) according to the manufacturer’s instructions [38]. Briefly, 600 μL of Buffer RLT Plus β-mercaptoethanol with a metal bead were added into each tube. Samples were then homogenized using the Retsch Tissue Lyser (Qiagen) at 30/frequency for 3 min and followed by 3 min centrifugation at maximum speed (Eppendorf). Supernatants were transferred into all Prep DNA mini spin column and centrifuged at 10.000 rpm for 30 sec. DNA attached to spin columns (Qiagen) were washed/de-salted using 500 μl of Buffer AW1 and Buffer AW2. Then, DNA samples were eluted with 30 μl EB buffer (Qiagen) into 1.5 ml microfuge tubes. The concentrations and purity of the isolated DNA was evaluated using NanoDrop® (ThermoScientific).

### Definition of mild and severe disease course

Classification of disease severity was based on parameters suggested by Peyrin-Biroulet *et al*. as well on data availability in the SIBDC database [39]. In detail, we included the following patient parameters: history of intestinal surgery, steroid-dependent/steroid-refractory disease or at least 6 months continuous treatment with corticosteroids (prednisone, prednisolone, cortisol), use of at least two anti-TNF antibodies, pancolitis in UC patients or the presence of L4, B2 or B3 according to disease location involvement (L1:ileal, L2:colonic, L3:ileocolonic, L4: upper gastrointestinal) and disease behavior (B1:inflammatory, B2:stricturing, and B3:penetrating–both internal and perianal) based on Montreal classification in CD patients as well as hospitalization due to IBD [40–42]. A mild disease course was defined by the absence of any of those clinical criteria and a severe disease course was defined by the presence of at least one of those clinical criteria.

### Microbial profiling of biopsies and multivariate statistical analyses

Ileum and colon biopsies of each patient were processed and were sequenced for the V5/V6 region of the 16S rRNA genes with the barcoded forward primer in combination with the reverse primer, as described elsewhere [43]. The 16s PCR amplicons were pooled at 26pM and was prepared for sequencing on the Ion torrent PGM system according to the manufacturer’s instructions (ThermoFisher) [44].

Raw sequences of samples with over 5000 reads were first loaded into the QIIME 1.9.1 pipeline, as described [45]. Sequences were initially clustered into ~350,000 taxa based on their shared sequence similarity at 3% sequence divergence. Operational taxonomic units (OTUs) were picked using UCLUST with a 97% sequence identity threshold followed by taxonomy assignment using the latest Greengenes database from May, 2013. (http://greengenes.secondgenome.com/downloads).

Further analysis of alpha diversity (Observed OTUs, Simpson and Shannon index) and beta diversity (Bray-Curtis dissimilarities) were performed in the *phyloseq* pipeline using R (v3.4) including vegan [46], multtest [47], and graphed via the *ggplot2* packages [48]. The non-parametric Mann-Whitney U-tests used to assess alpha diversity and Adonis from vegan package (Permanova method on distances; 9999 permutations used for F-tests based on continuous sums of squares from permutations of the original data) were used to assess the statistical significance of groups for beta diversity analysis. A p-value < 0.05 was considered significant. Multivariate homogeneity of groups dispersions analysis calculated the average distance of test groups and identified whether the dispersions of the groups were similar and p>0.05 value in this analysis was considered as the samples had the same dispersion.

The associations of microbial abundances (at phylum and genus levels) with clinical metadata and PTPN variants were analyzed using multivariate analysis by linear models (MaAsLin) R package [49]. A false discovery rate (FDR; Benjamini-Hochberg false discovery rate correction) of 0.05, taxa present in at least 30% of the samples and OTUs that had more than %0.0001 of total counts were set as the cut-off value for significance. Significant taxa were plotted as arcsin(x)-square root transformed microbial relative abundances.

## Results

### Demographic aspects

Swiss IBD cohort study (SIBDCS) comprises ~1000 patients from all regions of Switzerland that have been followed prospectively for more than 10 years. In order to characterize the microbial community of IBD patients, among those patients, we have selected IBD patients that were sampled at more than one location along the cephalocaudal axis of the distal gut during the time attending for colonoscopy. We have initially processed 941 biopsy samples of 346 IBD patients obtained from the cohort. 314 of the 941 samples were initially excluded from further analysis due to insufficient number of reads (<5000). Further exclusion criteria were the age of the patients (samples in the range of 1^st^ and 3^rd^ quartiles of overall population) and gender balance, since we aimed at obtaining a homogenous distribution. Lastly, we set our focus on ileum and colon (combination of ascending colon, transversal colon, and descending colon) biopsy samples for microbiota analysis. Filtering out low-quality samples (samples with mean quality score <25) with excluding non-bacterial reads (filtered with filter_taxa_from_otu_table.py command in QIIME) after balancing the age/gender variables to eliminate the cofounder effect we generated high-quality reads (44057 reads per sample in average for CD and 30696 reads per samples for UC group) of 292 samples from ileum and colon biopsies of 75 CD patients (24–45 years old) and 57 UC patients (28–52 years old). The extensive information on all clinical characteristics and medication use are presented in **Table 1**.

**Table 1:** Clinical characteristics of Swiss IBD cohort patients in numbers.

	CD	UC
**Number of Patient (Number of Biopsies)**	75 (144)	57 (148)
**Gender** (% female)	61	60
**Mean age at enrolment** (years +/-SD)	33.8 (+/-7.8)	40.8 (+/-7.1)
**Mean age at diagnosis** (years +/- SD)	23.6 (+/-7)	30.5 (+/-7.7)
**PTPN2 (TT—CT)**	55–20	41–16
**PTPN22 (GG—GA)**	64–11	51–6
**Mean BMI**		
Normal (<25 Kg/m^2^)	57	39
Overweight (>25 Kg/m^2^, <30 Kg/m^2^)	14	13
Obese (>30 Kg/m^2^)	4	4
Unknown	-	1
**Mean disease duration at enrolment** (years +/- SD)	10.2 (+/-8.3)	10.3 (+/-6.8)
**Anatomic Location (Ileum—Colon)**	33–42	26–31
**Endoscopical Assessment**		
Inflamed / Non-inflamed	35 / 109	40 / 108
**Disease behaviour** (Montréal Classification)		
Inflammatory (B1)	34	-
Stricturing (B2)	20	-
Penetrating (B3)	20	-
Unknown	1	-
**Disease location**		
CD		
Ileum (L1)	26	-
Colon (L2)	17	-
Ileum & Colon (L3)	8	-
Upper GI Involvement (L4)	2	-
Unknown	2	2
UC		
Proctitis	-	11
Left-sided colitis	-	19
Pancolitis	-	26
**Medical treatment (**current)		
Anti-TNF-α agents	21	7
Systemic corticosteroids	22	11
5-ASA	7	20
Methotrexate	3	-
Thiopurines	11	18
**Number of patient in complete remission**	67	45

### IBD-associated risk variants have a direct impact on gut microbial profile

We initially used the genotype information of the IBD-associated risk variants rs1893217 within the PTPN2 gene locus and rs2476601 within the PTPN22 gene locus for investigating the effect of these variants on gut microbial changes of IBD patients. Even though PTPN22 is significantly associated with the risk of developing CD, but has no association with UC we have included this variant in our analysis to see whether it has any effect on shaping microbiota of UC patients [50]. The respective genotyping had already been previously performed by the SIBDC. The PTPN2 polymorphism rs1893217 [51] occurs in homozygous wild-type (TT), heterozygous (CT) and homozygous variant (CC) genotypes while the PTPN22 polymorphism rs2476601 [52] occurs in homozygous wild-type (GG), heterozygous (GA) and homozygous variant (AA) genotypes. Due to the low number of homozygous variant numbers (1–3 patients) in each IBD-associated risk variant, homozygous variants were included into heterozygous variant category.

**Fig 1:**
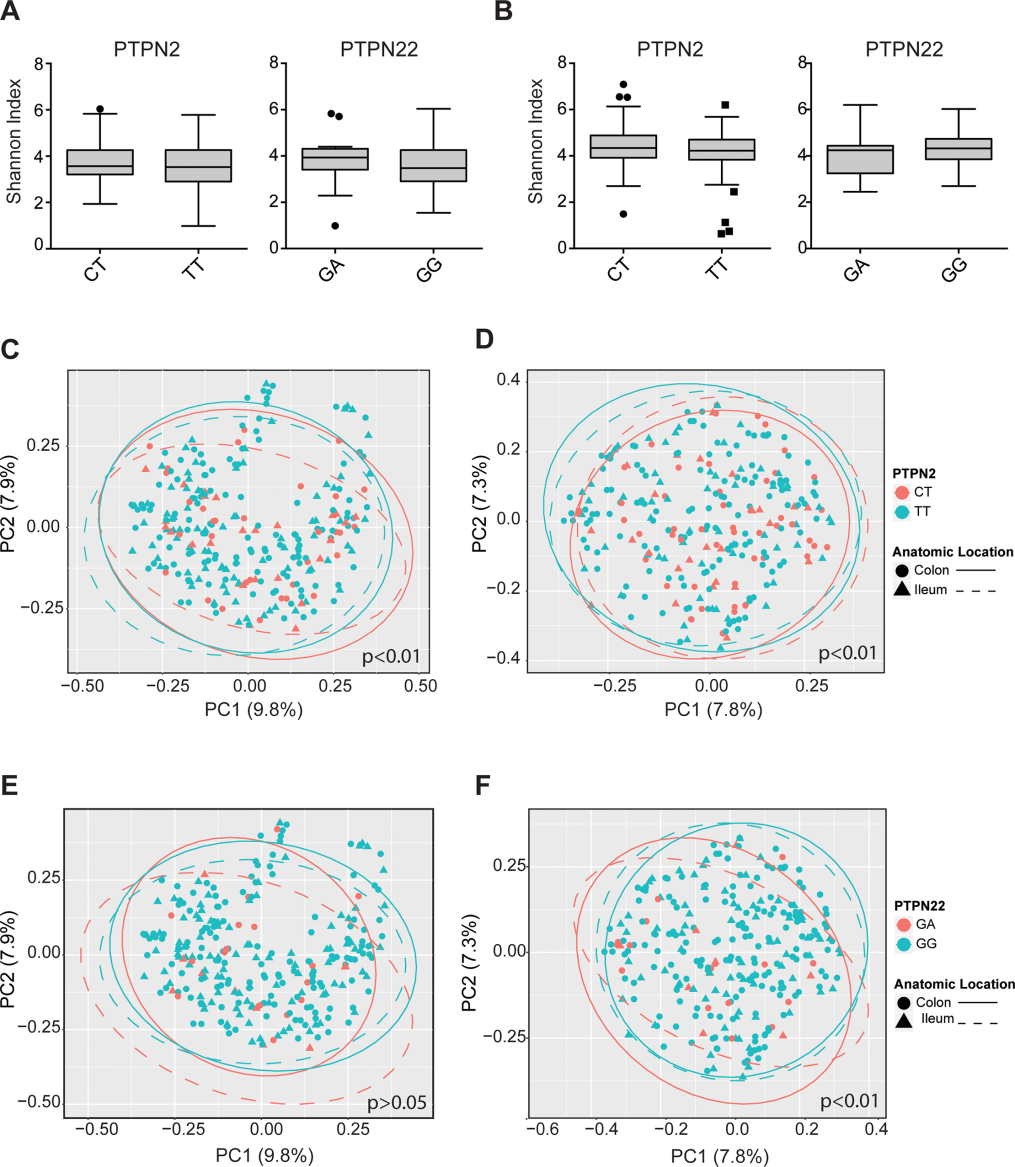
The microbial features associated with disease phenotype and IBD-associated risk variant rs1893217 and rs2476601. Species richness is calculated using Shannon index in CD (A) and UC (B) samples. Beta diversity is calculated for CD (C) and UC (D) samples in the context of PTPN2 variant and for CD (E) and UC (F) samples in the context of PTPN22 variant using Bray-Curtis dissimilarity matrix. Heterozygous variants are red color while homozygous wild-type ones are turquoise. Colon samples are labeled as closed circle and ileum samples are labeled as closed triangles. Ellipses represent dispersion of samples and ellipses for colon samples are labeled with straight line while ellipses for ileum samples are labeled are label dashed line. A p-value <0.05 is considered significant. Significant differences are marked through the panels.

We first identified the microbial features associated with disease phenotype and IBD-associated risk variant rs1893217 within the PTPN2 gene locus and rs2476601 within the PTPN22 gene using MaAsLin pipeline that takes into account the possible effect of clinical metadata as confounding factors in microbiota analysis and runs statistical analysis accordingly. We ran the association analysis of a specific microbial community member with clinical metadata (Table 1) including age, gender, body-mass index (BMI), anatomic location, tissue inflammation status, disease treatment categorized according to administration of anti-TNF-α agents or systemic corticosteroids. We detected no significant differences in alpha diversity metrics including Shannon index, Observed OTU and Simpson Index for CD **(Fig 1A and Fig A in S1 Fig)** and UC patients **(Fig 1B and Fig B in S1 Fig)** when analyzed individually for each risk variants. However, we noted a wider range of baseline community diversity for those in homozygous wild-type (WT) genotypes. There was no significant difference in species richness and diversity within the communities when different IBD-risk variant groups were compared. However, the PCoA plots showed that gut microbial composition differentiated in PTPN2 variant samples of CD patients (Adonis; p<0.01) **(Fig 1C)** but this was not the case for PTPN22 variant samples of CD patients (Adonis; p>0.05) **(Fig 1E).** In contrast, the PCoA plots showed that gut microbial composition differentiated in PTPN2 and PTPN22 variants samples of UC patients (Adonis; p<0.01) (**Fig 1D and Fig 1F)**. The percentage on axis (varying from 7–10%) showed the proportion of variance explained with PC1 or PC2 axis. We further analysed multivariate homogeneity of the groups on the basis of an appropriate measure of dissimilarity which shows non-Euclidean distances between samples and group centroids. Beta dispersion analysis showed that dispersion between CT and TT group in CD samples (p = 0.465), dispersion between CT and TT group in UC samples (p = 0.225) and dispersion between variant allele and wild-type genotypes in UC samples (p = 0.285) were not significantly different **(Fig 1C–1F)**. This allows us to conclude that the observed diversity between samples of variant groups is not due to differences in group dispersions. Overall, PTPN2 variant associated with changes on intestinal microbiota of CD and UC patients while PTPN22 variant affects only intestinal microbiota of UC patients.

**Fig 2:**
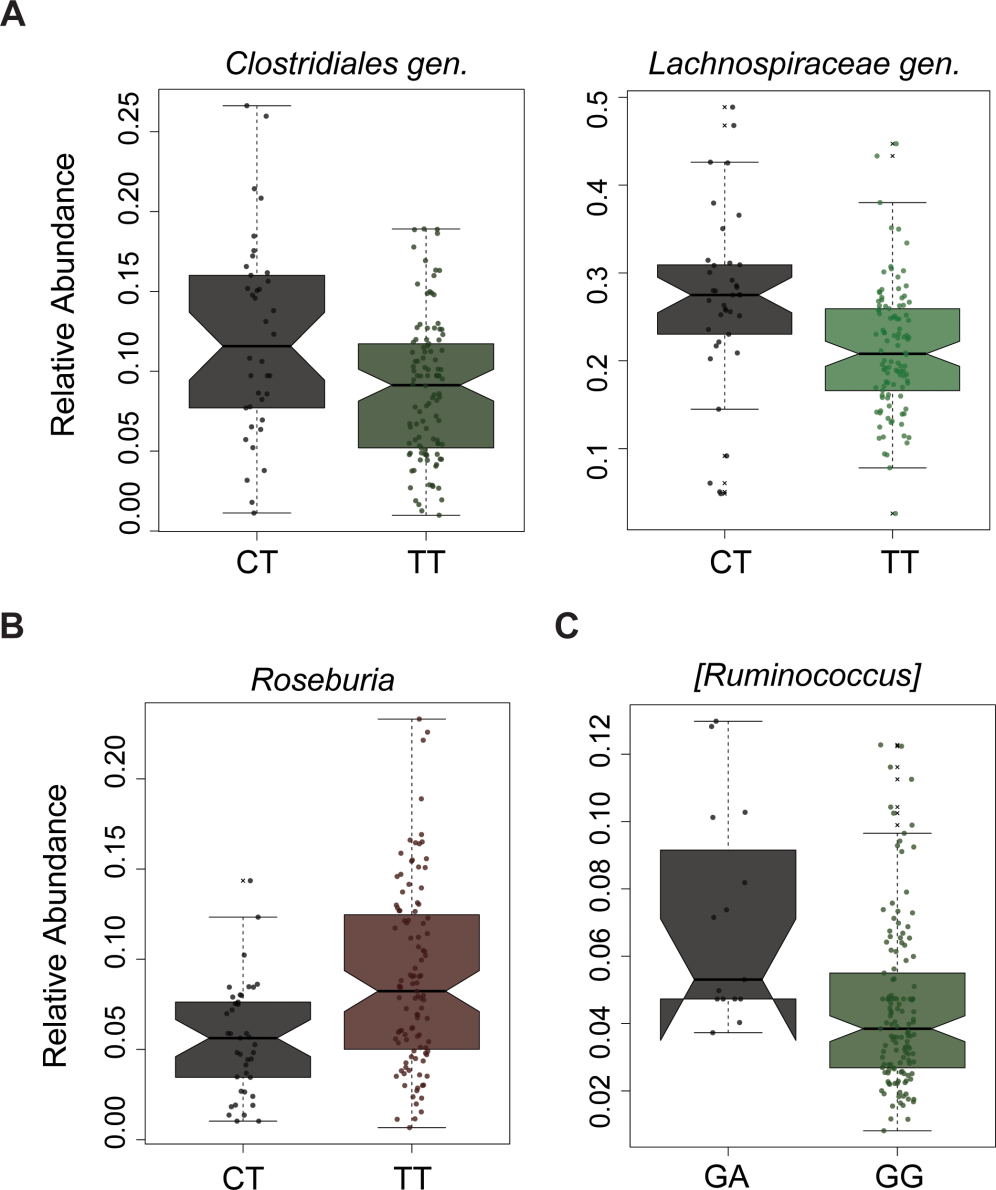
The microbial differences associated with disease and risk variants. The relative abundance of significantly different taxa (q<0.05) were plotted for PTPN2 variant in (A) when analyzed for CD and in (B) when analyzed for UC. The relative abundance of significantly different taxa (q<0.05) was plotted for PTPN22 variants when analyzed for UC (C) samples. The “*gen*.,” was used for the classification of a distinct but unnamed genus in the Greengenes reference database and “unclassified” used for identification of unclassified taxa in Greengenes reference database, as previously used in another study [53].

We next compared the relative abundances (% composition of a particular bacteria relative to bacteria in the sample) of CD samples **(S1 Table)** and UC samples **(S2 Table)** with their respective PTPN variant status. There were no taxa significantly different at phylum rank in both disease conditions. However, at genus rank, several taxa were significantly different in relative abundance according to genotype and disease status **(Fig 2)**. There was an increase in relative abundance of *Clostridiales gen*. and *Lachnospiraceae gen*. in CD patients with the CT genotype in PTPN2 **(Fig 2A and S1 Table)**. On the other hand, the relative abundance of *Roseburia* was reduced in UC patients carrying the CT genotype in PTPN2 gene **(Fig 2B and S2 Table).** *Ruminoccocus* was increased in UC patients carrying the GA genotype in PTPN22 variant **(Fig 2C and S2 Table) (**BH adjusted-p < 0.05). In CD patients, when analyzed according to their PTPN2 genotype, several bacteria including *Bacteroides*, *Desulfovibrionaceae gen*., *Bifidobacterium*, *Megasphaera*, *Mogibacteriaceae gen*, *Actinomyces*, *Erysipelotrichaceae_gen*, *Lachnospira*, *Oscillospira*, *Phascolarctobacterium*, *Akkermansia*, and *Roseburia* showed trends of relative abundance changes (p<0.05, BH adjusted-p > 0.05) **(S1 Table)**. However, only a few taxa, namely *Epulopiscium*, *Neisseria*, *Lactococcus*, and *Rothia* showed trends of relative abundance changes (p<0.05, BH adjusted-p > 0.05) when analyzed according to PTPN22 genotype (**S1 Table)**. By contrast, there were a few taxa identified in UC patients showing the trends of relative abundance changes (p<0.05, BH adjusted-p > 0.05). In addition, in UC patients, *Pseudomonas*, *Staphylococcus*, and *Dialister* showed a trend of relative abundance changes (p<0.05, BH adjusted-p >0.05) when analyzed for PTPN2 genotypes (**S2 Table)**, while *Lactobacillus* showed a trend of relative abundance changes (p<0.05, BH adjusted-p >0.05) when analyzed for PTPN22 genotype (**S2 Table)**. Overall, this hints on that the presence of genetic risk variants within PTPN2 and PTPN22 might have a role on affecting the intestinal flora. However, the overall low number of samples in heterozygous genotypes may be considered as a plausible reason underlying significant differences only in unadjusted testing in some genera analyzed.

### Disease severity and its interaction with PTPN polymorphisms affect several taxa

It has been documented that different complex microbiota can alter disease severity in mouse models of IBD [54]. Therefore, we next analyzed whether gut microbial profiles of IBD patients with different PTPN2 or PTPN22 variants were affected by disease severity.

We initially categorized disease severity into two categories, namely mild and severe suggested by Peyrin-Biroulet *et al*.[39] and investigated whether IBD-associated risk variants and disease severity are associated with gut microbiota alterations in CD and UC **(Table 2)**. Of the entire set of patients, we have mainly low number of samples in severe disease group of each disease condition for patients with PTPN22 heterozygous variant. Due to the numbers of samples specifically in severe UC patients (< 3 patients) with heterozygous variant carriers, the comparisons of microbial relative abundance in these groups were excluded from further analysis.

**Table 2:** The number of CD and UC patients within different disease severity condition and PTPN variants.

Disease Severity	CD (Number of Patients)		UC (Number of Patients)	
	CT / TT	GA / GG	CT / TT	GA / GG
**Mild**	7 / 28	7 / 28	13 / 29	4 / 38
**Severe**	13 / 27	4 / 36	3 / 12	2 / 13

**Fig 3:**
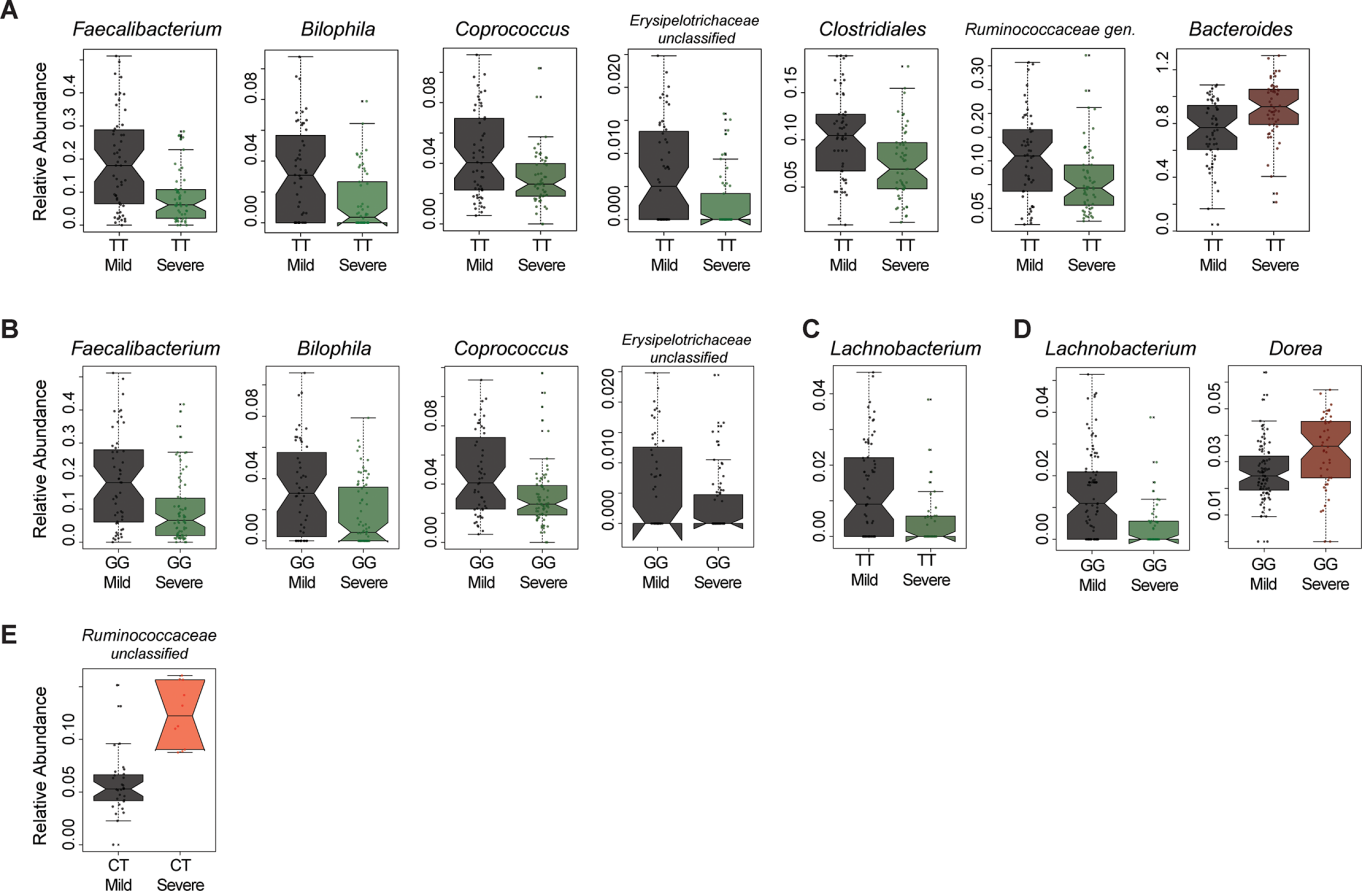
The microbial differences associated with disease severity within same PTPN disease variants. The relative abundance of significantly different microbial taxa (q<0.05) were plotted according to disease severity status for PTPN2 wild-type variant in (A) and PTPN22 wild type variant in (B) when analyzed for CD. The relative abundance of significantly different microbial taxa (q<0.05) were plotted according to disease severity status PTPN2 wild-type variant in (C) and heterozygous variant (D) and PTPN22 wild-type variant in (E) when analyzed for UC.

In taxonomical analysis of CD patients, there were significant changes only observed in TT but not CT genotype in PTPN2 **(Fig 3A and S3 Table)** and in GG but not GA genotype in PTPN22 **(Fig 3B and S4 Table)** when compared according to disease severity status of the patients. The relative abundances of *Faecalibacterium*, *Bilophila*, *Coprococcus*, unclassified *Erysipelotrichaeceae*, *Clostridales gen*., and *Ruminococcaceae gen*., were reduced, while *Bacteroides* taxa were increased in samples from CD patients with severe disease activity carrying PTPN2 TT genotype (BH adjusted-p <0.05) **(Fig 3A)**. Moreover, relative abundances of *Faecalibacterium*, *Bilophila*, *Coprococcus*, and unclassified *Erysipelotrichaeceae* were significantly reduced in samples carrying GG genotype of PTPN22 (BH adjusted-p <0.05) **(Fig 3B)** from CD patients with elevated disease severity.

In contrast, the number of changes in relative abundance in UC samples categorized according to disease severity and IBD-associated risk variants were lower. The relative abundance of *Lachnobacterium* was reduced in samples from patients with TT genotype of PTPN2 **(Fig 3C and S5 Table)** and GG genotype of PTPN22 **(Fig 3D and S6 Table)** with severe disease activity (BH adjusted-p <0.05), while *Dorea* was increased in samples from GG genotype in PTPN22. In addition, the abundance of *Ruminococcaceae gen*. in UC samples from patients with severe disease activity was significantly influenced by the host’s genetic makeup (BH adjusted-p <0.05) **(Fig 3E and S5 Table)** since this taxon was not significantly different in wild-type variant.

## Discussion

In the present study, we analysed the effects of the IBD-associated variants within the gene loci encoding PTPN2 and PTPN22 on intestinal microbiota composition. We found that the presence of variant alleles significantly affects intestinal microbiota composition. The intestinal microbiota alteration was not only affected by PTPN genotypes, but it also was affected by interaction of genotype and disease severity course suggesting a direct link for a genotype-microbiota interaction affecting disease course in IBD patients.

Alterations in intestinal microbiota composition, so-called dysbiosis, have been associated with a broad number of diseases, including obesity, diabetes, rheumatoid arthritis, asthma, depression, malignant diseases, irritable bowel syndrome (IBS) and IBD [55]. Intestinal dysbiosis is particularly one of the most important factors contributing to the onset of the IBD subtypes, CD and UC [9] and a number of studies documented significant differences in intestinal microbiota composition between healthy people and IBD patients [56, 57]. Although there are some overlaps in the respective dysbiotic alterations, there are also considerable differences in intestinal microbiota changes between both diseases [58–60]. *Walker et al*. demonstrated that microbial diversity is reduced in UC and CD patients accompanied by a reduction of *Firmicutes* and an increase in *Bacteroidetes* in all IBD patients and a selective elevation in the abundance of *Enterobacteriaceae* in CD patients [59]. In UC patients an enrichment of putative aggressive *Proteobacteria* and *Fusobacterium* spp. and a decreased abundance of protective bacteria, such as *Lachnospiraceae*, *Ruminococcaceae*, *Bifidobacterium* spp. or *Roseburia* has been shown [9]. Though a role for intestinal bacteria for the pathogenesis of IBD has been well described nowadays, the interplay between genetic risk factors and intestinal microbiota is less clear [9]. Nevertheless, more and more evidence suggests that genetic IBD risk factors, in particular NOD2 and ATG16L1, critically interact with the intestinal microbiota to maintain intestinal homeostasis and to control immune responses in the gastrointestinal tract [10, 11].

We have recently demonstrated that PTPN2 as well as PTPN22 regulate autophagosome function, including ATG16L1 protein expression, and cell responses following activation of NOD2 [17, 19, 61]. Furthermore, the presence of the IBD-associated genetic variants within the gene loci encoding PTPN2 and PTPN22 affects the disease course in IBD patients and PTPN2-deficient mice exhibit intestinal microbiota changes comparable to those observed in IBD patients [16, 23, 27]. In good accordance with those previous observations, we found that the presence of PTPN2 variant affects the gut microbial composition in UC and CD patients, while the presence of the PTPN22 variant affects microbial composition mainly in UC patients. On a genus level, the PTPN2 variant is associated with an increase in relative abundance of *Clostridiales gen*. and *Lachnospiraceae* gen. in CD patients and a decrease in the relative abundance of *Roseburia* in UC patients. Further, we found the increased relative abundance of *Ruminoccocus* in PTPN22 variant patients. In particular, the increased abundance of *Ruminoccocus* in PTPN22 variant patients can be explained by the protective effect of this variant. These data are of particular interest, since the physiologic role for PTPN2 and PTPN22 in the intestinal tract suggests a crucial role for both of the phosphatases in regulating immune responses to intestinal bacteria and in controlling the intestinal immune system. Genetically caused alterations in PTPN2/PTPN22 protein function might therefore directly contribute to the altered microbiota composition that favors the onset of IBD.

**Fig 4:**
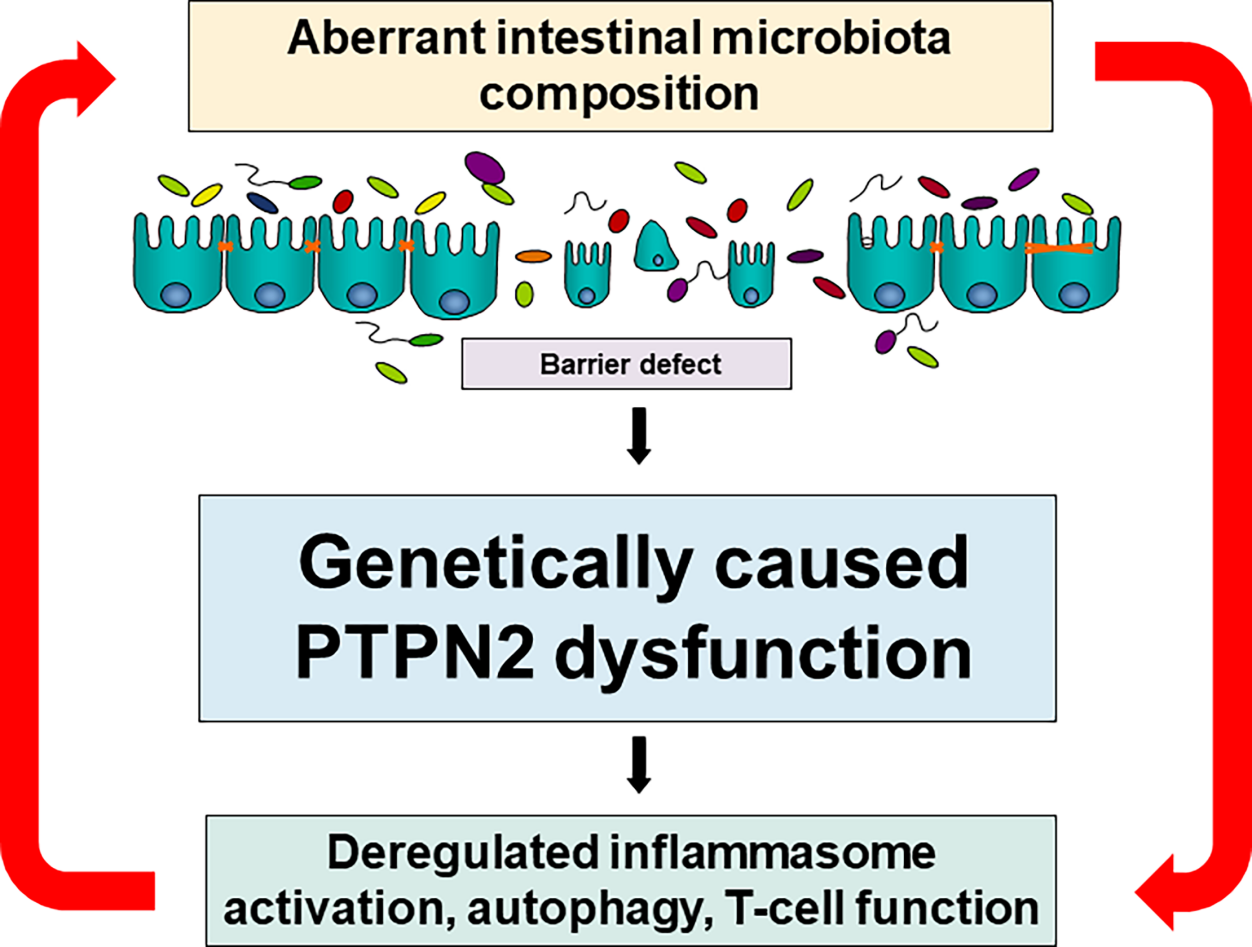
Effects of PTPN2 dysfunction on intestinal microbiota composition. PTPN2-mediated dysfunction of several immune mechanisms that are important for the body’s immune response to luminal bacteria and for maintaining intestinal homeostasis. In particular, dysfunction of autophagy, aberrant inflammasome activation and altered T-cell activation and differentiation seems to play a crucial role for the observed correlation between PTPN2 dysfunction and alterations in intestinal microbiota composition.

The aim of the present study was to determine whether the alterations in intestinal microbiota composition observed in mouse colitis models in T-cell specific PTPN2 deficient mice [16] are comparable to the alterations in intestinal microbiota composition observed in IBD patients. Our data clearly demonstrate comparable effects in PTPN2 knock-out mice and PTPN2 variant carriers. We anticipate that this is due to PTPN2-mediated dysfunction of several immune mechanisms that are important for the body’s immune response to luminal bacteria and for maintaining intestinal homeostasis. In particular, dysfunction of autophagy, aberrant inflammasome activation and altered T-cell activation and differentiation seems to play a crucial role for the observed correlation between PTPN2 dysfunction and alterations in intestinal microbiota composition **(Fig 4)** [16, 17, 62].

We also found differences in intestinal microbiota composition between PTPN wild-type genotype and heterozygous genotype samples of patients with severe disease course. These observations hint on that altered expression levels and/or protein function of e.g. antimicrobial molecules in patients featuring the heterozygous gene variants might have a role on controlling these taxa in severe disease activity conditions. However, though those taxa were not significantly different from each other in heterozygous variants, we must keep in mind that this might be due to the fact that there were low number of samples from heterozygous variant carriers.

Of interest, we found that *Bacteroides* was increased in samples with more severe disease while *Ruminococcaceae gen*. was reduced in samples from severe CD patients with TT genotype in PTPN2. Further, we detected that the abundance of *Ruminococcaceae gen*. showed a trend of reduction with severe disease activity in samples from UC patients with TT genotype in PTPN2. This finding is of particular interest, since *Ruminococcaceae gen*. are regarded as “good” bacteria and a reduction of those bacteria might be a part of the detrimental effect of the PTPN2 variation in human disease. The reduction of *Feaecalibacterium*, *Coprococcus* and *Lachnobacterium* in severe UC patients with the wild-type PTPN22 genotype makes sense in a way that the reduction of those bacteria is regarded as being characteristic for the disease and the preservation of those bacteria in PTPN22 variant patients might be part of the protective role of the PTPN22 variation in human disease.

Our results in addition seem to provide evidence for a causative role of intestinal microbial alterations in patients with IBD as opposed to solely reflecting a sequela of inflammation within the intestinal mucosa (the so called “chicken and egg” question). Our data further suggest, that established risk genes associated with the development of IBD might profoundly impact intestinal microbial composition irrespective of disease activity. Thus, our results point in the same direction as the findings by other groups demonstrating, a microbiota-shaping potential of the Nod2-gene[63], alterations in IBD-risk-gene carriers in the healthy population [64], altered microbiota in siblings of CD patients [65] similar microbial alterations in inflamed and non-inflamed mucosal compartments [32] and therefore indicate a causative role of microbial alterations in IBD pathogenesis. However, the results also point towards a dominant role of disease severity over a certain genotype of the affected patient. Nevertheless, the fact that in our study significant differences were found in mild vs. severe cases of CD in patients only with WT genotypes may be, at least in part, related to the fact that CT (PTPN2) or GA (PTPN22) groups became very small after stratification to mild and severe cases.

Overall, our data show that presence of the IBD-associated variations within the PTPN2 and PTPN22 gene loci are related to significant alterations in intestinal microbiota composition what clearly resembles our previous findings in mouse colitis models using PTPN2 deficient mice. Further, variations within the PTPN2 gene loci are correlated with disease severity in CD patients. Those findings suggest a critical role for PTPN2 and PTPN22 in modulating intestinal microbiota composition and point towards a novel gene (PTPN2)-microbiota interaction directly affecting disease severity in IBD patients. In conclusion, our data indicate a novel role for PTPN2 and PTPN22 in controlling intestinal microbiota composition and further elucidate the complex interplay between genetic risk factors, intestinal microbiota and disease course in IBD patients.

## Supporting information

S1 Fig Alpha diversity measurements in IBD samples. Observed OTU and Simpson index are calculated for PTPN variants. Species richness are compared in (A) for CD samples and in (B) for UC samples.
(PDF)

S1 Table Comparison of relative abundance of CD samples at phylum and genus rank calculated using MaAsLin. Taxonomic difference of PTPN variants in CD disease group was identified and significant and non-significant differences were recorded based on MaAsLin output file. Table shows coefficient value for each taxa and number of samples that were analyzed. A p-value <0.05 is considered significant.
(PDF)

S2 Table Comparison of relative abundance of UC samples at phylum and genus rank calculated using MaAsLin. Taxonomic difference of PTPN variants in UC disease group was identified and significant and non-significant differences were recorded based on MaAsLin output file. Table shows coefficient value for each taxa and number of samples that were analyzed. A p-value <0.05 is considered significant.
(PDF)

S3 Table Comparison of relative abundances of different disease severities and PTPN2 variants in CD samples using MaAsLin. Taxonomic difference of PTPN2 variants in CD disease group was identified based on the disease severity status and significant and non-significant differences were recorded based on MaAsLin output file. Table shows coefficient value for each taxa and number of samples that were analyzed. A p-value <0.05 is considered significant.
(PDF)

S4 Table Comparison of relative abundances of different disease severities and PTPN22 variants in CD samples using MaAsLin. Taxonomic difference of PTPN22 variants in CD disease group was identified based on the disease severity status and significant and non-significant differences were recorded based on MaAsLin output file. Table shows coefficient value for each taxa and number of samples that were analyzed. A p-value <0.05 is considered significant.
(PDF)

S5 Table Comparison of relative abundances of different disease severities and PTPN2 variants in UC samples using MaAsLin. Taxonomic difference of PTPN2 variants in UC disease group was identified based on the disease severity status and significant and non-significant differences were recorded based on MaAsLin output file. Table shows coefficient value for each taxa and number of samples that were analyzed. A p-value <0.05 is considered significant.
(PDF)

S6 Table Comparison of relative abundances of different disease severities and PTPN22 variants in UC samples using MaAsLin. Taxonomic difference of PTPN22 variants in UC disease group was identified based on the disease severity status and significant and non-significant differences were recorded based on MaAsLin output file. Table shows coefficient value for each taxa and number of samples that were analyzed. A p-value <0.05 is considered significant.
(PDF)

## References

[1] Hooper LV, Littman DR, Macpherson AJ. Interactions between the microbiota and the immune system. Science. 2012;336(6086):1268–73. doi: 10.1126/science.1223490 ; PubMed Central PMCID: PMC4420145. 22674334 10.1126/science.1223490 PMC4420145

[2] Yilmaz B, Portugal S, Tran TM, Gozzelino R, Ramos S, Gomes J, et al Gut microbiota elicits a protective immune response against malaria transmission. Cell. 2014;159(6):1277–89. doi: 10.1016/j.cell.2014.10.053 ; PubMed Central PMCID: PMC4261137. 25480293 10.1016/j.cell.2014.10.053 PMC4261137

[3] Clemente JC, Ursell LK, Parfrey LW, Knight R. The impact of the gut microbiota on human health: an integrative view. Cell. 2012;148(6):1258–70. doi: 10.1016/j.cell.2012.01.035 ; PubMed Central PMCID: PMCPMC5050011. 22424233 10.1016/j.cell.2012.01.035 PMC5050011

[4] Tamboli CP, Neut C, Desreumaux P, Colombel JF. Dysbiosis in inflammatory bowel disease. Gut. 2004;53(1):1–4. ; PubMed Central PMCID: PMCPMC1773911. 14684564 10.1136/gut.53.1.1 PMC1773911

[5] Kaur N, Chen CC, Luther J, Kao JY. Intestinal dysbiosis in inflammatory bowel disease. Gut Microbes. 2011;2(4):211–6. doi: 10.4161/gmic.2.4.17863 . 21983063 10.4161/gmic.2.4.17863

[6] Kamada N, Seo SU, Chen GY, Nunez G. Role of the gut microbiota in immunity and inflammatory disease. Nat Rev Immunol. 2013;13(5):321–35. Epub 2013/04/27. doi: 10.1038/nri3430 [pii]. . 23618829 10.1038/nri3430

[7] de Souza HS, Fiocchi C. Immunopathogenesis of IBD: current state of the art. Nat Rev Gastroenterol Hepatol. 2016;13(1):13–27. doi: 10.1038/nrgastro.2015.186 . 26627550 10.1038/nrgastro.2015.186

[8] Cleynen I, Boucher G, Jostins L, Schumm LP, Zeissig S, Ahmad T, et al Inherited determinants of Crohn's disease and ulcerative colitis phenotypes: a genetic association study. Lancet. 2016;387(10014):156–67. doi: 10.1016/S0140-6736(15)00465-1 ; PubMed Central PMCID: PMCPMC4714968. 26490195 10.1016/S0140-6736(15)00465-1 PMC4714968

[9] Sartor RB, Wu GD. Roles for Intestinal Bacteria, Viruses, and Fungi in Pathogenesis of Inflammatory Bowel Diseases and Therapeutic Approaches. Gastroenterology. 2017;152(2):327–39 e4. doi: 10.1053/j.gastro.2016.10.012 ; PubMed Central PMCID: PMCPMC5511756. 27769810 10.1053/j.gastro.2016.10.012 PMC5511756

[10] de Lange KM, Moutsianas L, Lee JC, Lamb CA, Luo Y, Kennedy NA, et al Genome-wide association study implicates immune activation of multiple integrin genes in inflammatory bowel disease. Nat Genet. 2017;49(2):256–61. doi: 10.1038/ng.3760 ; PubMed Central PMCID: PMCPMC5289481. 28067908 10.1038/ng.3760 PMC5289481

[11] Chu H, Khosravi A, Kusumawardhani IP, Kwon AH, Vasconcelos AC, Cunha LD, et al Gene-microbiota interactions contribute to the pathogenesis of inflammatory bowel disease. Science. 2016;352(6289):1116–20. doi: 10.1126/science.aad9948 ; PubMed Central PMCID: PMCPMC4996125. 27230380 10.1126/science.aad9948 PMC4996125

[12] Liu JZ, van Sommeren S, Huang H, Ng SC, Alberts R, Takahashi A, et al Association analyses identify 38 susceptibility loci for inflammatory bowel disease and highlight shared genetic risk across populations. Nat Genet. 2015;47(9):979–86. doi: 10.1038/ng.3359 ; PubMed Central PMCID: PMCPMC4881818. 26192919 10.1038/ng.3359 PMC4881818

[13] Genome-wide association study of 14,000 cases of seven common diseases and 3,000 shared controls. Nature. 2007;447(7145):661–78. Epub 2007/06/08. doi: nature05911 [pii] doi: 10.1038/nature05911 ; PubMed Central PMCID: PMC2719288. 17554300 10.1038/nature05911 PMC2719288

[14] Franke A, Balschun T, Karlsen TH, Hedderich J, May S, Lu T, et al Replication of signals from recent studies of Crohn's disease identifies previously unknown disease loci for ulcerative colitis. Nat Genet. 2008;40(6):713–5. Epub 2008/04/29. doi: ng.148 [pii] doi: 10.1038/ng.148 . 18438405 10.1038/ng.148

[15] Diaz-Gallo LM, Espino-Paisan L, Fransen K, Gomez-Garcia M, van Sommeren S, Cardena C, et al Differential association of two PTPN22 coding variants with Crohn's disease and ulcerative colitis. Inflamm Bowel Dis. 2011;17(11):2287–94. Epub 2011/02/03. doi: 10.1002/ibd.21630 . 21287672 10.1002/ibd.21630

[16] Spalinger MR, Kasper S, Chassard C, Raselli T, Frey-Wagner I, Gottier C, et al PTPN2 controls differentiation of CD4(+) T cells and limits intestinal inflammation and intestinal dysbiosis. Mucosal Immunol. 2015;8(4):918–29. doi: 10.1038/mi.2014.122 . 25492475 10.1038/mi.2014.122

[17] Scharl M, Wojtal KA, Becker HM, Fischbeck A, Frei P, Arikkat J, et al Protein tyrosine phosphatase nonreceptor type 2 regulates autophagosome formation in human intestinal cells. Inflamm Bowel Dis. 2012;18(7):1287–302. Epub 2011/10/12. doi: 10.1002/ibd.21891 . 21987459 10.1002/ibd.21891

[18] Scharl M, Paul G, Weber A, Jung BC, Docherty MJ, Hausmann M, et al Protection of epithelial barrier function by the Crohn's disease associated gene protein tyrosine phosphatase n2. Gastroenterology. 2009;137(6):2030–40 e5. Epub 2009/10/13. doi: S0016-5085(09)01757-0 [pii] doi: 10.1053/j.gastro.2009.07.078 ; PubMed Central PMCID: PMC2855721. 19818778 10.1053/j.gastro.2009.07.078 PMC2855721

[19] Scharl M, Mwinyi J, Fischbeck A, Leucht K, Eloranta JJ, Arikkat J, et al Crohn's disease-associated polymorphism within the PTPN2 gene affects muramyl-dipeptide-induced cytokine secretion and autophagy. Inflamm Bowel Dis. 2012;18(5):900–12. Epub 2011/10/25. doi: 10.1002/ibd.21913 . 22021207 10.1002/ibd.21913

[20] Scharl M, McCole DF, Weber A, Vavricka SR, Frei P, Kellermeier S, et al Protein tyrosine phosphatase N2 regulates TNFalpha-induced signalling and cytokine secretion in human intestinal epithelial cells. Gut. 2011;60(2):189–97. doi: 10.1136/gut.2010.216606 . 21115548 10.1136/gut.2010.216606

[21] Scharl M, Hruz P, McCole DF. Protein tyrosine phosphatase non-receptor Type 2 regulates IFN-gamma-induced cytokine signaling in THP-1 monocytes. Inflamm Bowel Dis. 2010;16(12):2055–64. Epub 2010/09/18. doi: 10.1002/ibd.21325 . 20848498 10.1002/ibd.21325

[22] Kasper SH, Spalinger MR, Leonardi I, Gerstgrasser A, Raselli T, Gottier C, et al Deficiency of Protein Tyrosine Phosphatase Non-Receptor Type 2 in Intestinal Epithelial Cells Has No Appreciable Impact on Dextran Sulphate Sodium Colitis Severity But Promotes Wound Healing. Digestion. 2016;93(4):249–59. doi: 10.1159/000445289 . 27115526 10.1159/000445289

[23] Spalinger MR, Voegelin M, Biedermann L, Zeitz J, Rossel JB, Sulz MC, et al The Clinical Relevance of the IBD-Associated Variation within the Risk Gene Locus Encoding Protein Tyrosine Phosphatase Non-Receptor Type 2 in Patients of the Swiss IBD Cohort. Digestion. 2016;93(3):182–92. doi: 10.1159/000444479 . 26928573 10.1159/000444479

[24] Spalinger MR, Lang S, Weber A, Frei P, Fried M, Rogler G, et al Loss of protein tyrosine phosphatase nonreceptor type 22 regulates interferon-gamma-induced signaling in human monocytes. Gastroenterology. 2013;144(5):978–88 e10. doi: 10.1053/j.gastro.2013.01.048 . 23380085 10.1053/j.gastro.2013.01.048

[25] Spalinger MR, Kasper S, Gottier C, Lang S, Atrott K, Vavricka SR, et al NLRP3 tyrosine phosphorylation is controlled by protein tyrosine phosphatase PTPN22. J Clin Invest. 2016;126(5):1783–800. doi: 10.1172/JCI83669 ; PubMed Central PMCID: PMCPMC4855944. 27043286 10.1172/JCI83669 PMC4855944

[26] Spalinger MR, Lang S, Gottier C, Dai X, Rawlings DJ, Chan AC, et al PTPN22 regulates NLRP3-mediated IL1B secretion in an autophagy dependent manner. Autophagy. 2017:0. doi: 10.1080/15548627.2017.1341453 . 28786745 10.1080/15548627.2017.1341453 PMC5612532

[27] Spalinger MR, Zeitz J, Biedermann L, Rossel JB, Sulz MC, Frei P, et al Genotype-Phenotype Associations of the CD-Associated Single Nucleotide Polymorphism within the Gene Locus Encoding Protein Tyrosine Phosphatase Non-Receptor Type 22 in Patients of the Swiss IBD Cohort. PLoS One. 2016;11(7):e0160215 doi: 10.1371/journal.pone.0160215 ; PubMed Central PMCID: PMCPMC4964985. 27467733 10.1371/journal.pone.0160215 PMC4964985

[28] Macpherson AJ, de Aguero MG, Ganal-Vonarburg SC. How nutrition and the maternal microbiota shape the neonatal immune system. Nat Rev Immunol. 2017;17(8):508–17. doi: 10.1038/nri.2017.58 . 28604736 10.1038/nri.2017.58

[29] Mazmanian SK, Liu CH, Tzianabos AO, Kasper DL. An immunomodulatory molecule of symbiotic bacteria directs maturation of the host immune system. Cell. 2005;122(1):107–18. Epub 2005/07/13. doi: S0092-8674(05)00451-4 [pii] doi: 10.1016/j.cell.2005.05.007 . 16009137 10.1016/j.cell.2005.05.007

[30] Mazmanian SK, Round JL, Kasper DL. A microbial symbiosis factor prevents intestinal inflammatory disease. Nature. 2008;453(7195):620–5. doi: 10.1038/nature07008 . 18509436 10.1038/nature07008

[31] Martinez C, Antolin M, Santos J, Torrejon A, Casellas F, Borruel N, et al Unstable composition of the fecal microbiota in ulcerative colitis during clinical remission. Am J Gastroenterol. 2008;103(3):643–8. doi: 10.1111/j.1572-0241.2007.01592.x . 18341488 10.1111/j.1572-0241.2007.01592.x

[32] Swidsinski A, Ladhoff A, Pernthaler A, Swidsinski S, Loening-Baucke V, Ortner M, et al Mucosal flora in inflammatory bowel disease. Gastroenterology. 2002;122(1):44–54. . 11781279 10.1053/gast.2002.30294

[33] Alhagamhmad MH, Day AS, Lemberg DA, Leach ST. An overview of the bacterial contribution to Crohn disease pathogenesis. J Med Microbiol. 2016;65(10):1049–59. doi: 10.1099/jmm.0.000331 . 27501828 10.1099/jmm.0.000331

[34] Sokol H, Seksik P, Furet JP, Firmesse O, Nion-Larmurier I, Beaugerie L, et al Low counts of Faecalibacterium prausnitzii in colitis microbiota. Inflamm Bowel Dis. 2009;15(8):1183–9. doi: 10.1002/ibd.20903 . 19235886 10.1002/ibd.20903

[35] Zoetendal EG, von Wright A, Vilpponen-Salmela T, Ben-Amor K, Akkermans AD, de Vos WM. Mucosa-associated bacteria in the human gastrointestinal tract are uniformly distributed along the colon and differ from the community recovered from feces. Applied and environmental microbiology. 2002;68(7):3401–7. Epub 2002/06/29. doi: 10.1128/AEM.68.7.3401-3407.2002 ; PubMed Central PMCID: PMCPMC126800. 12089021 10.1128/AEM.68.7.3401-3407.2002 PMC126800

[36] Momozawa Y, Deffontaine V, Louis E, Medrano JF. Characterization of bacteria in biopsies of colon and stools by high throughput sequencing of the V2 region of bacterial 16S rRNA gene in human. PloS one. 2011;6(2):e16952 Epub 2011/02/25. doi: 10.1371/journal.pone.0016952 ; PubMed Central PMCID: PMCPMC3037395. 21347324 10.1371/journal.pone.0016952 PMC3037395

[37] Pittet V, Juillerat P, Mottet C, Felley C, Ballabeni P, Burnand B, et al Cohort profile: the Swiss Inflammatory Bowel Disease Cohort Study (SIBDCS). Int J Epidemiol. 2009;38(4):922–31. doi: 10.1093/ije/dyn180 . 18782896 10.1093/ije/dyn180

[38] Gevers D, Kugathasan S, Denson LA, Vazquez-Baeza Y, Van Treuren W, Ren B, et al The treatment-naive microbiome in new-onset Crohn's disease. Cell host & microbe. 2014;15(3):382–92. doi: 10.1016/j.chom.2014.02.005 ; PubMed Central PMCID: PMCPMC4059512. 24629344 10.1016/j.chom.2014.02.005 PMC4059512

[39] Peyrin-Biroulet L, Panes J, Sandborn WJ, Vermeire S, Danese S, Feagan BG, et al Defining Disease Severity in Inflammatory Bowel Diseases: Current and Future Directions. Clin Gastroenterol Hepatol. 2016;14(3):348–54 e17. doi: 10.1016/j.cgh.2015.06.001 . 26071941 10.1016/j.cgh.2015.06.001

[40] Irwin J, Ferguson E, Simms LA, Hanigan K, Carbonnel F, Radford-Smith G. A rolling phenotype in Crohn's disease. PloS one. 2017;12(4):e0174954 Epub 2017/04/07. doi: 10.1371/journal.pone.0174954 ; PubMed Central PMCID: PMCPMC5383106. 28384331 10.1371/journal.pone.0174954 PMC5383106

[41] Silverberg MS, Satsangi J, Ahmad T, Arnott ID, Bernstein CN, Brant SR, et al Toward an integrated clinical, molecular and serological classification of inflammatory bowel disease: report of a Working Party of the 2005 Montreal World Congress of Gastroenterology. Can J Gastroenterol. 2005;19 Suppl A:5A–36A. . 16151544 10.1155/2005/269076

[42] Louis E, Collard A, Oger AF, Degroote E, Aboul Nasr El Yaf FA, Belaiche J. Behaviour of Crohn's disease according to the Vienna classification: changing pattern over the course of the disease. Gut. 2001;49(6):777–82. doi: 10.1136/gut.49.6.777 ; PubMed Central PMCID: PMC1728556. 11709511 10.1136/gut.49.6.777 PMC1728556

[43] Hai L, Limenitakis J.P., Fuhrer T., Geuking M.B., Lawson M.B., Wyss M., Brugiroux S., Keller I., Macpherson J.A., Rupp S., Stolp B., Stein J., Stecher B., Sauer U., McCoy K.D., Macpherson A.J. The outer mucus layer hosts a distinct intestinal microbial niche. Nature Communications. 2015. 10.1038/ncomms9292 PMC4595636 26392213

[44] Whiteley AS, Jenkins S, Waite I, Kresoje N, Payne H, Mullan B, et al Microbial 16S rRNA Ion Tag and community metagenome sequencing using the Ion Torrent (PGM) Platform. J Microbiol Methods. 2012;91(1):80–8. doi: 10.1016/j.mimet.2012.07.008 . 22849830 10.1016/j.mimet.2012.07.008

[45] Caporaso JG, Kuczynski J, Stombaugh J, Bittinger K, Bushman FD, Costello EK, et al QIIME allows analysis of high-throughput community sequencing data. Nat Methods. 2010;7(5):335–6. doi: 10.1038/nmeth.f.303 ; PubMed Central PMCID: PMCPMC3156573. 20383131 10.1038/nmeth.f.303 PMC3156573

[46] Oksanen J. Multivariate analysis of ecological communities in R: vegan tutoria. 2015.

[47] Pollard K.S. D S, van der Laan M.J. Multiple Testing Procedures: the multtest Package and Applications to Genomics In: Gentleman R. CVJ, Huber W., Irizarry R.A., Dudoit S., editor. Bioinformatics and Computational Biology Solutions Using R and Bioconductor Statistics for Biology and Health New York: Springer, New York, NY; 2005.

[48] Wickham Hadley. ggplot2: Elegant Graphics for Data Analysis: Springer International Publishing; 2016.

[49] Morgan XC, Tickle TL, Sokol H, Gevers D, Devaney KL, Ward DV, et al Dysfunction of the intestinal microbiome in inflammatory bowel disease and treatment. Genome Biol. 2012;13(9):R79 Epub 2012/09/28. doi: 10.1186/gb-2012-13-9-r79 ; PubMed Central PMCID: PMCPMC3506950. 23013615 10.1186/gb-2012-13-9-r79 PMC3506950

[50] Hedjoudje A, Cheurfa C, Briquez C, Zhang A, Koch S, Vuitton L. rs2476601 polymorphism in PTPN22 is associated with Crohn's disease but not with ulcerative colitis: a meta-analysis of 16,838 cases and 13,356 controls. Ann Gastroenterol. 2017;30(2):197–208. doi: 10.20524/aog.2017.0121 ; PubMed Central PMCID: PMCPMC5320033. 28243041 10.20524/aog.2017.0121 PMC5320033

[51] Spalinger MR, Voegelin M, Biedermann L, Zeitz J, Rossel JB, Sulz MC, et al The Clinical Relevance of the IBD-Associated Variation within the Risk Gene Locus Encoding Protein Tyrosine Phosphatase Non-Receptor Type 2 in Patients of the Swiss IBD Cohort. Digestion. 2016;93(3):182–92. doi: 10.1159/000444479 PubMed PMID: WOS:000375772100003. 26928573 10.1159/000444479

[52] Spalinger MR, Zeitz J, Biedermann L, Rossel JB, Sulz MC, Frei P, et al Genotype-Phenotype Associations of the CD-Associated Single Nucleotide Polymorphism within the Gene Locus Encoding Protein Tyrosine Phosphatase Non-Receptor Type 22 in Patients of the Swiss IBD Cohort. PloS one. 2016;11(7). doi: ARTN e0160215 doi: 10.1371/journal.pone.0160215 PubMed PMID: WOS:000381516100131. 27467733 10.1371/journal.pone.0160215 PMC4964985

[53] Palm NW, de Zoete MR, Cullen TW, Barry NA, Stefanowski J, Hao L, et al Immunoglobulin A coating identifies colitogenic bacteria in inflammatory bowel disease. Cell. 2014;158(5):1000–10. doi: 10.1016/j.cell.2014.08.006 ; PubMed Central PMCID: PMCPMC4174347. 25171403 10.1016/j.cell.2014.08.006 PMC4174347

[54] Hart ML, Ericsson AC, Franklin CL. Differing Complex Microbiota Alter Disease Severity of the IL-10-/- Mouse Model of Inflammatory Bowel Disease. Front Microbiol. 2017;8:792 doi: 10.3389/fmicb.2017.00792 ; PubMed Central PMCID: PMCPMC5425584. 28553262 10.3389/fmicb.2017.00792 PMC5425584

[55] Selber-Hnatiw S, Rukundo B, Ahmadi M, Akoubi H, Al-Bizri H, Aliu AF, et al Human Gut Microbiota: Toward an Ecology of Disease. Front Microbiol. 2017;8:1265 doi: 10.3389/fmicb.2017.01265 ; PubMed Central PMCID: PMCPMC5511848. 28769880 10.3389/fmicb.2017.01265 PMC5511848

[56] Wright EK, Kamm MA, Teo SM, Inouye M, Wagner J, Kirkwood CD. Recent advances in characterizing the gastrointestinal microbiome in Crohn's disease: a systematic review. Inflamm Bowel Dis. 2015;21(6):1219–28. doi: 10.1097/MIB.0000000000000382 ; PubMed Central PMCID: PMCPMC4450900. 25844959 10.1097/MIB.0000000000000382 PMC4450900

[57] Qin J, Li R, Raes J, Arumugam M, Burgdorf KS, Manichanh C, et al A human gut microbial gene catalogue established by metagenomic sequencing. Nature. 2010;464(7285):59–65. doi: 10.1038/nature08821 ; PubMed Central PMCID: PMCPMC3779803. 20203603 10.1038/nature08821 PMC3779803

[58] Frank DN, St Amand AL, Feldman RA, Boedeker EC, Harpaz N, Pace NR. Molecular-phylogenetic characterization of microbial community imbalances in human inflammatory bowel diseases. Proc Natl Acad Sci U S A. 2007;104(34):13780–5. doi: 10.1073/pnas.0706625104 ; PubMed Central PMCID: PMCPMC1959459. 17699621 10.1073/pnas.0706625104 PMC1959459

[59] Walker AW, Sanderson JD, Churcher C, Parkes GC, Hudspith BN, Rayment N, et al High-throughput clone library analysis of the mucosa-associated microbiota reveals dysbiosis and differences between inflamed and non-inflamed regions of the intestine in inflammatory bowel disease. BMC Microbiol. 2011;11:7 doi: 10.1186/1471-2180-11-7 ; PubMed Central PMCID: PMCPMC3032643. 21219646 10.1186/1471-2180-11-7 PMC3032643

[60] Swidsinski A, Loening-Baucke V, Vaneechoutte M, Doerffel Y. Active Crohn's disease and ulcerative colitis can be specifically diagnosed and monitored based on the biostructure of the fecal flora. Inflamm Bowel Dis. 2008;14(2):147–61. doi: 10.1002/ibd.20330 . 18050295 10.1002/ibd.20330

[61] Spalinger MR, Lang S, Vavricka SR, Fried M, Rogler G, Scharl M. Protein Tyrosine Phosphatase Non-Receptor Type 22 Modulates NOD2-Induced Cytokine Release and Autophagy. PLoS One. 2013;8(8):e72384 doi: 10.1371/journal.pone.0072384 ; PubMed Central PMCID: PMC3753240. 23991106 10.1371/journal.pone.0072384 PMC3753240

[62] Spalinger MR, Manzini R, Hering L, Riggs JB, Gottier C, Lang S, et al PTPN2 Regulates Inflammasome Activation and Controls Onset of Intestinal Inflammation and Colon Cancer. Cell Rep. 2018;22(7):1835–48. doi: 10.1016/j.celrep.2018.01.052 . 29444435 10.1016/j.celrep.2018.01.052 PMC6636824

[63] Rehman A, Sina C, Gavrilova O, Hasler R, Ott S, Baines JF, et al Nod2 is essential for temporal development of intestinal microbial communities. Gut. 2011;60(10):1354–62. doi: 10.1136/gut.2010.216259 . 21421666 10.1136/gut.2010.216259

[64] Imhann F, Vich Vila A, Bonder MJ, Fu J, Gevers D, Visschedijk MC, et al Interplay of host genetics and gut microbiota underlying the onset and clinical presentation of inflammatory bowel disease. Gut. 2016 doi: 10.1136/gutjnl-2016-312135 . 27802154 10.1136/gutjnl-2016-312135 PMC5699972

[65] Hedin CR, McCarthy NE, Louis P, Farquharson FM, McCartney S, Taylor K, et al Altered intestinal microbiota and blood T cell phenotype are shared by patients with Crohn's disease and their unaffected siblings. Gut. 2014;63(10):1578–86. doi: 10.1136/gutjnl-2013-306226 . 24398881 10.1136/gutjnl-2013-306226

