# Supplementary figures and images for: The presence of genetic risk variants within PTPN2 and PTPN22 is associated with intestinal microbiota alterations in Swiss IBD cohort patients
Source: PLoS One. 2018 Jul 2;13(7):e0199664. doi: 10.1371/journal.pone.0199664 (PMC6028086; doi:10.1371/journal.pone.0199664)

**A**

PTPN2

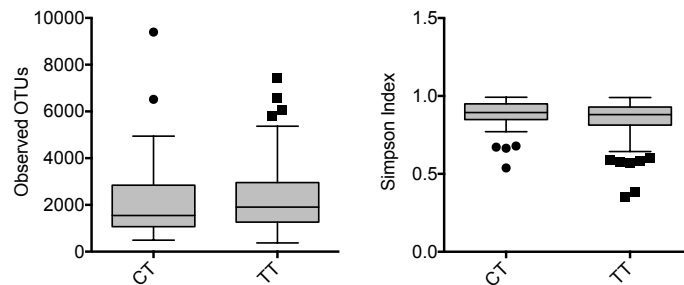

PTPN22

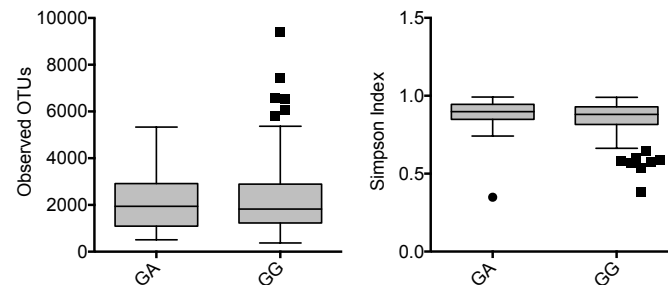**B**

PTPN2

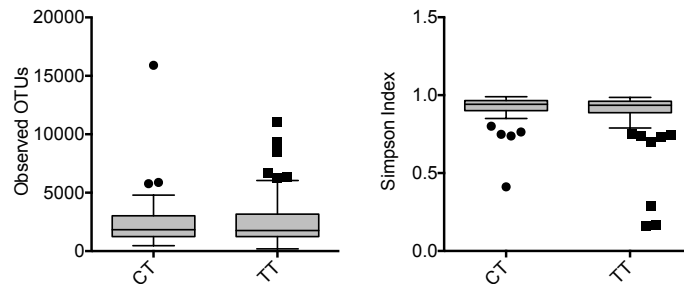

PTPN22

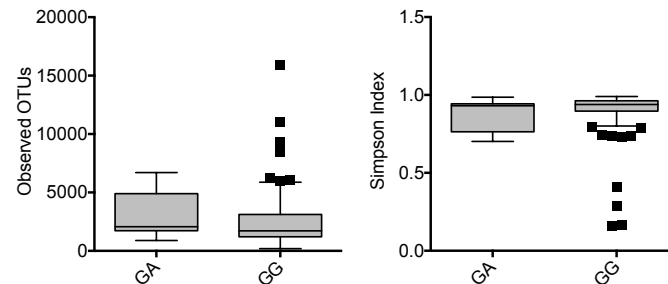

Supplement: S1 Fig — Observed OTU and Simpson index are calculated for PTPN variants. Species richness are compared in (A) for CD samples and in (B) for UC samples. (PDF) [file pone.0199664.s001.pdf]
