# Supplementary material for: The presence of genetic risk variants within PTPN2 and PTPN22 is associated with intestinal microbiota alterations in Swiss IBD cohort patients
Source: PLoS One. 2018 Jul 2;13(7):e0199664. doi: 10.1371/journal.pone.0199664 (PMC6028086; doi:10.1371/journal.pone.0199664)
Supplement: S1 Table — Taxonomic difference of PTPN variants in CD disease group was identified and significant and non-significant differences were recorded based on MaAsLin output file. Table shows coefficient value for each taxa and number of samples that were analyzed. A p-value <0.05 is considered significant. (PDF) [file pone.0199664.s002.pdf]

Suppl. Table 1. Comparison of relative abundance of CD samples at phylum and genus rank calculated using MaAsLin

Variable	Feature	Value	Coefficient	N	N not 0	P-value
PTPN2	Bacteria|Firmicutes	PTPN2TT	-0.0937622	144	144	0.00375245
PTPN2	Bacteria|Proteobacteria	PTPN2TT	0.06589328	144	144	0.04291115
PTPN2	Bacteria|Bacteroidetes	PTPN2TT	0.06378359	144	144	0.10179051
PTPN2	Bacteria|Firmicutes|Clostridia|Clostridiales||	PTPN2TT	-0.0370205	144	144	6.47E-05
PTPN2	Bacteria|Firmicutes|Clostridia|Clostridiales|Lachnospiraceae|	PTPN2TT	-0.0555682	144	144	0.00031274
PTPN2	Bacteria|Bacteroidetes|Bacteroidia|Bacteroidales|Bacteroidaceae|Bacteroides	PTPN2TT	0.14796423	144	144	0.00152667
PTPN2	Bacteria|Proteobacteria|Deltaproteobacteria|Desulfovibrionales|Desulfovibrionaceae|	PTPN2TT	-0.0045445	144	51	0.00226962
PTPN2	Bacteria|Actinobacteria|Actinobacteria|Bifidobacteriales|Bifidobacteriaceae|Bifidobacterium	PTPN2TT	0.00735306	144	114	0.00318391
PTPN2	Bacteria|Firmicutes|Clostridia|Clostridiales|Veillonellaceae|Megasphaera	PTPN2TT	-0.0022799	144	50	0.00414306
PTPN2	Bacteria|Firmicutes|Clostridia|Clostridiales|[Mogibacteriaceae]|	PTPN2TT	-0.0027019	144	65	0.01113443
PTPN2	Bacteria|Actinobacteria|Actinobacteria|Actinomycetales|Actinomycetaceae|Actinomyces	PTPN2TT	-0.0034547	144	87	0.0159728
PTPN2	Bacteria|Firmicutes|Erysipelotrichi|Erysipelotrichales|Erysipelotrichaceae|	PTPN2TT	0.01265071	144	141	0.0170577
PTPN2	Bacteria|Firmicutes|Clostridia|Clostridiales|Lachnospiraceae|Lachnospira	PTPN2TT	0.00899359	144	120	0.01822859
PTPN2	Bacteria|Firmicutes|Clostridia|Clostridiales|Ruminococcaceae|Oscillospira	PTPN2TT	-0.0121487	144	140	0.01879921
PTPN2	Bacteria|Firmicutes|Clostridia|Clostridiales|Veillonellaceae|Phascolarctobacterium	PTPN2TT	-0.0191871	144	118	0.02568454
PTPN2	Bacteria|Verrucomicrobia|Verrucomicrobiae|Verrucomicrobiales|Verrucomicrobiaceae|Akkermansia	PTPN2TT	-0.0059895	144	96	0.03025748
PTPN2	Bacteria|Firmicutes|Clostridia|Clostridiales|Lachnospiraceae|Roseburia	PTPN2TT	0.0146273	144	139	0.03840091
PTPN2	Bacteria|Firmicutes|Clostridia|Clostridiales|Ruminococcaceae|Anaerotruncus	PTPN2TT	-0.0019796	144	57	0.06279705
PTPN2	Bacteria|Firmicutes|Clostridia|Clostridiales|Lachnospiraceae|Unclassified	PTPN2TT	-0.014675	144	144	0.10949932
PTPN2	Bacteria|Bacteroidetes|Bacteroidia|Bacteroidales||	PTPN2TT	-0.0010848	144	55	0.109903
PTPN2	Bacteria|Firmicutes|Bacilli|Gemellales|Gemellaceae|Unclassified	PTPN2TT	-0.0012974	144	71	0.11403868
PTPN2	Bacteria|Firmicutes|Clostridia|Clostridiales|Clostridiaceae|Unclassified	PTPN2TT	0.00256629	144	103	0.12147541
PTPN2	Bacteria|Firmicutes|Bacilli|Lactobacillales|Streptococcaceae|Lactococcus	PTPN2TT	0.00167471	144	69	0.13531461
PTPN2	Bacteria|Firmicutes|Bacilli|Lactobacillales|Streptococcaceae|Streptococcus	PTPN2TT	-0.0043841	144	140	0.15355852
PTPN2	Bacteria|Actinobacteria|Actinobacteria|Actinomycetales|Micrococcaceae|Rothia	PTPN2TT	-0.0016024	144	66	0.20212528
PTPN2	Bacteria|Firmicutes|Bacilli|Lactobacillales|Enterococcaceae|Enterococcus	PTPN2TT	0.00138002	144	59	0.21755828
PTPN2	Bacteria|Firmicutes|Clostridia|Clostridiales|Peptostreptococcaceae|	PTPN2TT	0.00123197	144	73	0.22822526
PTPN2	Bacteria|Cyanobacteria|4C0d-2|YS2||	PTPN2TT	-0.001466	144	49	0.23677972
PTPN2	Bacteria|Firmicutes|Bacilli|Lactobacillales|Streptococcaceae|Unclassified	PTPN2TT	0.00078646	144	46	0.24789621
PTPN2	Bacteria|Firmicutes|Clostridia|Clostridiales|Ruminococcaceae|Unclassified	PTPN2TT	-0.0064096	144	134	0.25004661
PTPN2	Bacteria|Firmicutes|Erysipelotrichi|Erysipelotrichales|Erysipelotrichaceae|Allobaculum	PTPN2TT	0.0007147	144	49	0.33347862
PTPN2	Bacteria|Bacteroidetes|Bacteroidia|Bacteroidales|Rikenellaceae|	PTPN2TT	-0.0086102	144	140	0.39184985
PTPN2	Bacteria|Proteobacteria|Gammaproteobacteria|Pseudomonadales|Moraxellaceae|Acinetobacter	PTPN2TT	-0.0011955	144	75	0.40421877
PTPN2	Bacteria|Actinobacteria|Coriobacteriia|Coriobacteriales|Coriobacteriaceae|Adlercreutzia	PTPN2TT	-0.0005458	144	44	0.46352087
PTPN2	Bacteria|Firmicutes|Bacilli|Lactobacillales|Lactobacillaceae|Lactobacillus	PTPN2TT	-0.0026235	144	120	0.47625675
PTPN2	Bacteria|Firmicutes|Clostridia|Clostridiales|Ruminococcaceae|Ruminococcus	PTPN2TT	-0.0045856	144	142	0.49008825
PTPN2	Bacteria|Proteobacteria|Gammaproteobacteria|Enterobacteriales|Enterobacteriaceae|Gluconacetobacter	PTPN2TT	0.00037464	144	46	0.62532817
PTPN2	Bacteria|Firmicutes|Clostridia|Clostridiales|Veillonellaceae|Acidaminococcus	PTPN2TT	0.00019825	144	52	0.71539965
PTPN2	Bacteria|Bacteroidetes|Bacteroidia|Bacteroidales|[Paraprevotellaceae]|[Prevotella]	PTPN2TT	-0.0004212	144	107	0.73136376
PTPN2	Bacteria|Firmicutes|Clostridia|Clostridiales|Veillonellaceae|Dialister	PTPN2TT	0.0012512	144	91	0.74092954
PTPN2	Bacteria|Firmicutes|Clostridia|Clostridiales|Lachnospiraceae|Blautia	PTPN2TT	-0.002645	144	144	0.7597833
PTPN2	Bacteria|Firmicutes|Clostridia|Clostridiales|Lachnospiraceae|Lachnobacterium	PTPN2TT	0.00013573	144	50	0.80855923
PTPN2	Bacteria|Firmicutes|Bacilli|Bacillales|Unclassified|Unclassified	PTPN2TT	-0.0002515	144	99	0.8859671
PTPN22	Bacteria|Verrucomicrobia	PTPN22GG	-0.0088813	144	97	0.01901026
PTPN22	Bacteria|Firmicutes	PTPN22GG	-0.0116831	144	144	0.7865017
PTPN22	Bacteria|Firmicutes|Clostridia|Clostridiales|Lachnospiraceae|Epulopiscium	PTPN22GG	0.00439509	144	91	0.00692548
PTPN22	Bacteria|Proteobacteria|Betaproteobacteria|Neisseriales|Neisseriaceae|Neisseria	PTPN22GG	-0.003305	144	65	0.01322214
PTPN22	Bacteria|Firmicutes|Bacilli|Lactobacillales|Streptococcaceae|Lactococcus	PTPN22GG	-0.003455	144	69	0.02822296
PTPN22	Bacteria|Actinobacteria|Actinobacteria|Actinomycetales|Micrococcaceae|Rothia	PTPN22GG	-0.0036241	144	66	0.03167643
PTPN22	Bacteria|Verrucomicrobia|Verrucomicrobiae|Verrucomicrobiales|Verrucomicrobiaceae|Akkermansia	PTPN22GG	-0.007286	144	96	0.0587959
PTPN22	Bacteria|Firmicutes|Bacilli|Lactobacillales|Enterococcaceae|Enterococcus	PTPN22GG	-0.0029647	144	59	0.05898402
PTPN22	Bacteria|Firmicutes|Bacilli|Turicibacterales|Turicibacteraceae|Turicibacter	PTPN22GG	-0.0034004	144	62	0.07341242
PTPN22	Bacteria|Bacteroidetes|Bacteroidia|Bacteroidales|[Paraprevotellaceae]|[Prevotella]	PTPN22GG	-0.0023039	144	107	0.16832729
PTPN22	Bacteria|Proteobacteria|Gammaproteobacteria|Pasteurellales|Pasteurellaceae|Actinobacillus	PTPN22GG	-0.0025524	144	83	0.23301187
PTPN22	Bacteria|Firmicutes|Bacilli|Bacillales|Unclassified|Unclassified	PTPN22GG	-0.0027948	144	99	0.24606982
PTPN22	Bacteria|Firmicutes|Clostridia|Clostridiales|Ruminococcaceae|Anaerotruncus	PTPN22GG	-0.001627	144	57	0.27191202
PTPN22	Bacteria|Proteobacteria|Alphaproteobacteria|RF32||	PTPN22GG	-0.0070532	144	89	0.33149719
PTPN22	Bacteria|Firmicutes|Clostridia|Clostridiales|[Mogibacteriaceae]|	PTPN22GG	-0.0002556	144	65	0.85718506
PTPN22	Bacteria|Proteobacteria|Deltaproteobacteria|Desulfovibrionales|Desulfovibrionaceae|	PTPN22GG	1.64E-05	144	51	0.9935961
