# Supplementary material for: The presence of genetic risk variants within PTPN2 and PTPN22 is associated with intestinal microbiota alterations in Swiss IBD cohort patients
Source: PLoS One. 2018 Jul 2;13(7):e0199664. doi: 10.1371/journal.pone.0199664 (PMC6028086; doi:10.1371/journal.pone.0199664)
Supplement: S2 Table — Taxonomic difference of PTPN variants in UC disease group was identified and significant and non-significant differences were recorded based on MaAsLin output file. Table shows coefficient value for each taxa and number of samples that were analyzed. A p-value <0.05 is considered significant. (PDF) [file pone.0199664.s003.pdf]

Suppl. Table 2. Comparison of relative abundance of UC samples at phylum and genus rank calculated using MaAsLin

Variable	Feature	Value	Coefficient	N	N not 0	P-value
PTPN2	Bacteria|Tenericutes	PTPN2TT	-0.0046467	147	101	0.10990211
PTPN2	Bacteria|Firmicutes|Clostridia|Clostridiales|Lachnospiraceae|Roseburia	PTPN2TT	0.03924651	148	148	3.83E-06
PTPN2	Bacteria|Proteobacteria|Gammaproteobacteria|Pseudomonadales|Pseudomonadaceae|Pseudomonas	PTPN2TT	-0.0024535	148	72	0.02308222
PTPN2	Bacteria|Firmicutes|Bacilli|Bacillales|Staphylococcaceae|Staphylococcus	PTPN2TT	-0.0027025	148	75	0.02829586
PTPN2	Bacteria|Firmicutes|Clostridia|Clostridiales|Veillonellaceae|Dialister	PTPN2TT	0.00758187	148	118	0.04143988
PTPN2	Bacteria|Firmicutes|Erysipelotrichi|Erysipelotrichales|Erysipelotrichaceae|[Eubacterium]	PTPN2TT	-0.0123887	148	135	0.05222396
PTPN2	Bacteria|Actinobacteria|Coriobacteriia|Coriobacteriales|Coriobacteriaceae|Collinsella	PTPN2TT	0.0077863	148	133	0.10652241
PTPN2	Bacteria|Cyanobacteria|Chloroplast|Streptophyta||	PTPN2TT	-0.0015694	148	48	0.11434118
PTPN2	Bacteria|Fusobacteria|Fusobacteriia|Fusobacteriales|Leptotrichiaceae|Leptotrichia	PTPN2TT	0.00097171	148	48	0.13606897
PTPN2	Bacteria|Firmicutes|Clostridia|Clostridiales|Ruminococcaceae|Faecalibacterium	PTPN2TT	0.03131739	148	148	0.16369912
PTPN2	Bacteria|Firmicutes|Bacilli|Lactobacillales|Streptococcaceae|Lactococcus	PTPN2TT	0.00118391	148	52	0.17513486
PTPN2	Bacteria|Proteobacteria|Gammaproteobacteria|Pasteurellales|Pasteurellaceae|Aggregatibacter	PTPN2TT	0.0018306	148	57	0.17758986
PTPN2	Bacteria|Firmicutes|Clostridia|Clostridiales|Lachnospiraceae|Oribacterium	PTPN2TT	0.00117815	148	46	0.19630888
PTPN2	Bacteria|Firmicutes|Clostridia|Clostridiales|Veillonellaceae|	PTPN2TT	0.00121658	148	53	0.21132038
PTPN2	Bacteria|Firmicutes|Erysipelotrichi|Erysipelotrichales|Erysipelotrichaceae|Unclassified	PTPN2TT	-0.0018237	148	79	0.217825
PTPN2	Bacteria|Firmicutes|Clostridia|Clostridiales|Veillonellaceae|Acidaminococcus	PTPN2TT	-0.0006712	148	45	0.23374862
PTPN2	Bacteria|Actinobacteria|Coriobacteriia|Coriobacteriales|Coriobacteriaceae|Adlercreutzia	PTPN2TT	-0.0012747	148	52	0.23610136
PTPN2	Bacteria|Firmicutes|Clostridia|Clostridiales|Christensenellaceae|	PTPN2TT	-0.0051727	148	88	0.24403165
PTPN2	Bacteria|Firmicutes|Bacilli|Lactobacillales|Enterococcaceae|Enterococcus	PTPN2TT	0.00102933	148	52	0.27822576
PTPN2	Bacteria|Verrucomicrobia|Verrucomicrobiae|Verrucomicrobiales|Verrucomicrobiaceae|Akkermansia	PTPN2TT	0.00321109	148	115	0.28083327
PTPN2	Bacteria|Proteobacteria|Alphaproteobacteria|RF32||	PTPN2TT	-0.0068201	148	97	0.30613266
PTPN2	Bacteria|Firmicutes|Clostridia|Clostridiales|Lachnospiraceae|Lachnospira	PTPN2TT	-0.0043794	148	143	0.31932501
PTPN2	Bacteria|Firmicutes|Erysipelotrichi|Erysipelotrichales|Erysipelotrichaceae|	PTPN2TT	0.00454496	148	144	0.36635693
PTPN2	Bacteria|Proteobacteria|Gammaproteobacteria|Enterobacteriales|Enterobacteriaceae|Unclassified	PTPN2TT	0.00154696	148	73	0.43860427
PTPN2	Bacteria|Proteobacteria|Betaproteobacteria|Neisseriales|Neisseriaceae|Neisseria	PTPN2TT	0.00089888	148	73	0.45056469
PTPN2	Bacteria|Proteobacteria|Gammaproteobacteria|Unclassified|Unclassified|Unclassified	PTPN2TT	0.00043767	148	47	0.56394304
PTPN2	Bacteria|Firmicutes|Clostridia|Clostridiales|Veillonellaceae|Megasphaera	PTPN2TT	0.0006128	148	50	0.58417625
PTPN2	Bacteria|Bacteroidetes|Bacteroidia|Bacteroidales|[Odoribacteraceae]|Odoribacter	PTPN2TT	-0.0030852	148	131	0.60355608
PTPN2	Bacteria|Bacteroidetes|Bacteroidia|Bacteroidales|Porphyromonadaceae|Porphyromonas	PTPN2TT	0.0006133	148	55	0.61050179
PTPN2	Bacteria|Firmicutes|Bacilli|Turicibacterales|Turicibacteraceae|Turicibacter	PTPN2TT	-0.0005828	148	71	0.65118984
PTPN2	Bacteria|Proteobacteria|Gammaproteobacteria|Enterobacteriales|Enterobacteriaceae|Klebsiella	PTPN2TT	0.00035003	148	49	0.67517818
PTPN2	Bacteria|Firmicutes|Clostridia|Clostridiales|Lachnospiraceae|[Ruminococcus]	PTPN2TT	-0.0016368	148	148	0.73104226
PTPN2	Bacteria|Firmicutes|Clostridia|Clostridiales|Ruminococcaceae|Oscillospira	PTPN2TT	0.00169285	148	147	0.77818439
PTPN2	Bacteria|Bacteroidetes|Bacteroidia|Bacteroidales|Prevotellaceae|Prevotella	PTPN2TT	0.00885563	148	147	0.77891759
PTPN2	Bacteria|Bacteroidetes|Bacteroidia|Bacteroidales|[Paraprevotellaceae]|Paraprevotella	PTPN2TT	-0.0008717	148	64	0.82944167
PTPN2	Bacteria|Firmicutes|Clostridia|Clostridiales|Lachnospiraceae|Lachnobacterium	PTPN2TT	-0.0003184	148	88	0.88190951
PTPN22	Bacteria|Tenericutes	PTPN22GG	0.01123989	147	101	0.00556536
PTPN22	Bacteria|Verrucomicrobia	PTPN22GG	0.00582947	147	115	0.2308361
PTPN22	Bacteria|Firmicutes|Clostridia|Clostridiales|Lachnospiraceae|[Ruminococcus]	PTPN22GG	-0.0266518	148	148	0.00018566
PTPN22	Bacteria|Firmicutes|Bacilli|Lactobacillales|Lactobacillaceae|Lactobacillus	PTPN22GG	0.01279886	148	131	0.00704024
PTPN22	Bacteria|Bacteroidetes|Bacteroidia|Bacteroidales|S24-7|	PTPN22GG	0.01939505	148	144	0.06603612
PTPN22	Bacteria|Firmicutes|Erysipelotrichi|Erysipelotrichales|Erysipelotrichaceae|Unclassified	PTPN22GG	-0.0037752	148	79	0.08170659
PTPN22	Bacteria|Firmicutes|Clostridia|Clostridiales|Lachnospiraceae|Dorea	PTPN22GG	-0.0049673	148	144	0.12775769
PTPN22	Bacteria|Firmicutes|Erysipelotrichi|Erysipelotrichales|Erysipelotrichaceae|[Eubacterium]	PTPN22GG	-0.0095737	148	135	0.29691033
PTPN22	Bacteria|Firmicutes|Erysipelotrichi|Erysipelotrichales|Erysipelotrichaceae|	PTPN22GG	0.00735095	148	144	0.31858307
