# Supplementary material for: The presence of genetic risk variants within PTPN2 and PTPN22 is associated with intestinal microbiota alterations in Swiss IBD cohort patients
Source: PLoS One. 2018 Jul 2;13(7):e0199664. doi: 10.1371/journal.pone.0199664 (PMC6028086; doi:10.1371/journal.pone.0199664)
Supplement: S3 Table — Taxonomic difference of PTPN2 variants in CD disease group was identified based on the disease severity status and significant and non-significant differences were recorded based on MaAsLin output file. Table shows coefficient value for each taxa and number of samples that were analyzed. A p-value <0.05 is considered significant. (PDF) [file pone.0199664.s004.pdf]

Suppl. Table 3. Comparison of relative abundances of different disease severities and PTPN2 variants in CD samples using MaAsLin

Variable	Feature	Value	Coefficient	N	N not 0	P-value	Q-value
PTPN2_Severity	Bacteria|Firmicutes|Clostridia|Clostridiales|Ruminococcaceae|Faecalibacterium	PTPN2_SeverityTT_Severe	-0.1062683	106	102	7.19E-06	0.00065452
PTPN2_Severity	Bacteria|Proteobacteria|Deltaproteobacteria|Desulfovibrionales|Desulfovibrionaceae|Bilophila	PTPN2_SeverityTT_Severe	-0.0185925	106	65	0.00025094	0.00712509
PTPN2_Severity	Bacteria|Firmicutes|Clostridia|Clostridiales|	PTPN2_SeverityTT_Severe	-0.0298648	106	106	0.00033902	0.00712509
PTPN2_Severity	Bacteria|Firmicutes|Erysipelotrichi|Erysipelotrichales|Erysipelotrichaceae|Unclassified	PTPN2_SeverityTT_Severe	-0.0044598	106	41	0.00036253	0.00712509
PTPN2_Severity	Bacteria|Bacteroidetes|Bacteroidia|Bacteroidales|Bacteroidaceae|Bacteroides	PTPN2_SeverityTT_Severe	0.16576772	106	106	0.00039149	0.00712509
PTPN2_Severity	Bacteria|Firmicutes|Clostridia|Clostridiales|Lachnospiraceae|Coprococcus	PTPN2_SeverityTT_Severe	-0.015718	106	104	0.00098259	0.01490257
PTPN2_Severity	Bacteria|Firmicutes|Clostridia|Clostridiales|Ruminococcaceae|	PTPN2_SeverityTT_Severe	-0.0477457	106	106	0.00363797	0.0472936
PTPN2_Severity	Bacteria|Bacteroidetes|Bacteroidia|Bacteroidales|	PTPN2_SeverityTT_Severe	-0.0017937	106	36	0.01012001	0.11511511
PTPN2_Severity	Bacteria|Bacteroidetes|Bacteroidia|Bacteroidales|[Odoribacteraceae]|Butyricimonas	PTPN2_SeverityTT_Severe	-0.0056368	106	42	0.01292761	0.12640218
PTPN2_Severity	Bacteria|Bacteroidetes|Bacteroidia|Bacteroidales|Rikenellaceae|	PTPN2_SeverityTT_Severe	-0.0238074	106	102	0.01389035	0.12640218
PTPN2_Severity	Bacteria|Firmicutes|Clostridia|Clostridiales|Ruminococcaceae|Unclassified	PTPN2_SeverityTT_Severe	-0.0101847	106	101	0.03725036	0.30816211
PTPN2_Severity	Bacteria|Proteobacteria|Gammaproteobacteria|Pasteurellales|Pasteurellaceae|Haemophilus	PTPN2_SeverityTT_Severe	-0.0052235	106	99	0.04935864	0.36312759
PTPN2_Severity	Bacteria|Firmicutes|Clostridia|Clostridiales|Ruminococcaceae|Ruminococcus	PTPN2_SeverityTT_Severe	-0.0134845	106	104	0.05309119	0.36312759
PTPN2_Severity	Bacteria|Proteobacteria|Alphaproteobacteria|RF32|	PTPN2_SeverityTT_Severe	-0.0112705	106	64	0.05867082	0.36312759
PTPN2_Severity	Bacteria|Firmicutes|Clostridia|Clostridiales|Clostridiaceae|Clostridium	PTPN2_SeverityTT_Severe	-0.0026184	106	72	0.0598562	0.36312759
PTPN2_Severity	Bacteria|Firmicutes|Clostridia|Clostridiales|Ruminococcaceae|Anaerotruncus	PTPN2_SeverityTT_Severe	-0.0017214	106	37	0.06560956	0.3731544
PTPN2_Severity	Bacteria|Proteobacteria|Betaproteobacteria|Neisseriales|Neisseriaceae|Neisseria	PTPN2_SeverityTT_Severe	-0.0016674	106	46	0.07483006	0.4005609
PTPN2_Severity	Bacteria|Firmicutes|Clostridia|Clostridiales|Veillonellaceae|Veillonella	PTPN2_SeverityTT_Severe	0.00988823	106	86	0.08978381	0.40711915
PTPN2_Severity	Bacteria|Firmicutes|Bacilli|Lactobacillales|Streptococcaceae|Unclassified	PTPN2_SeverityTT_Severe	0.00122645	106	37	0.0906628	0.40711915
PTPN2_Severity	Bacteria|Bacteroidetes|Bacteroidia|Bacteroidales|[Paraprevotellaceae]|[Prevotella_	PTPN2_SeverityTT_Severe	0.00196517	106	79	0.09280899	0.40711915
PTPN2_Severity	Bacteria|Firmicutes|Erysipelotrichi|Erysipelotrichales|Erysipelotrichaceae|Coprobacillus	PTPN2_SeverityTT_Severe	-0.0012205	106	32	0.09880929	0.40711915
PTPN2_Severity	Bacteria|Actinobacteria|Actinobacteria|Actinomycetales|Propionibacteriaceae|Propionibacterium	PTPN2_SeverityTT_Severe	-0.0019118	106	60	0.10111086	0.40711915
PTPN2_Severity	Bacteria|Firmicutes|Clostridia|Clostridiales|Lachnospiraceae|[Ruminococcus_	PTPN2_SeverityTT_Severe	0.01325452	106	106	0.10742875	0.40711915
PTPN2_Severity	Bacteria|Firmicutes|Clostridia|Clostridiales|Lachnospiraceae|Anaerostipes	PTPN2_SeverityTT_Severe	-0.0019336	106	32	0.1096937	0.40711915
PTPN2_Severity	Bacteria|Firmicutes|Erysipelotrichi|Erysipelotrichales|Erysipelotrichaceae|[Eubacterium_	PTPN2_SeverityTT_Severe	-0.0057294	106	98	0.11184592	0.40711915
PTPN2_Severity	Bacteria|Firmicutes|Unclassified|Unclassified|Unclassified|Unclassified	PTPN2_SeverityTT_Severe	0.00139593	106	39	0.12027969	0.4209789
PTPN2_Severity	Bacteria|Firmicutes|Clostridia|Clostridiales|Lachnospiraceae|Epulopiscium	PTPN2_SeverityTT_Severe	-0.0018363	106	70	0.13907693	0.46874075
PTPN2_Severity	Bacteria|Bacteroidetes|Bacteroidia|Bacteroidales|[Odoribacteraceae]|Odoribacter	PTPN2_SeverityTT_Severe	-0.0108719	106	91	0.14450858	0.4696529
PTPN2_Severity	Bacteria|Bacteroidetes|Bacteroidia|Bacteroidales|Porphyromonadaceae|Parabacteroides	PTPN2_SeverityTT_Severe	-0.0232107	106	106	0.15116721	0.47435229
PTPN2_Severity	Bacteria|Proteobacteria|Gammaproteobacteria|Pseudomonadales|Moraxellaceae|Acinetobacter	PTPN2_SeverityTT_Severe	-0.0019948	106	52	0.16240235	0.48465073
PTPN2_Severity	Bacteria|Proteobacteria|Deltaproteobacteria|Desulfovibrionales|Desulfovibrionaceae|Desulfovibrio	PTPN2_SeverityTT_Severe	-0.0011515	106	46	0.16601417	0.48465073
PTPN2_Severity	Bacteria|Firmicutes|Erysipelotrichi|Erysipelotrichales|Erysipelotrichaceae|Allobaculum	PTPN2_SeverityTT_Severe	0.00109885	106	38	0.17042663	0.48465073
PTPN2_Severity	Bacteria|Proteobacteria|Gammaproteobacteria|Pasteurellales|Pasteurellaceae|Actinobacillus	PTPN2_SeverityTT_Severe	-0.0018928	106	61	0.18457865	0.49492868
PTPN2_Severity	Bacteria|Bacteroidetes|Bacteroidia|Bacteroidales|Unclassified|Unclassified	PTPN2_SeverityTT_Severe	-0.0030683	106	96	0.18935665	0.49492868
PTPN2_Severity	Bacteria|Firmicutes|Clostridia|Clostridiales|[Mogibacteriaceae]|	PTPN2_SeverityTT_Severe	-0.0011268	106	43	0.19035719	0.49492868
PTPN2_Severity	Bacteria|Firmicutes|Clostridia|Clostridiales|Christensenellaceae|	PTPN2_SeverityTT_Severe	-0.0011986	106	52	0.19806443	0.50066286
PTPN2_Severity	Bacteria|Firmicutes|Clostridia|Clostridiales|Lachnospiraceae|Unclassified	PTPN2_SeverityTT_Severe	-0.0113489	106	106	0.22673932	0.55765616
PTPN2_Severity	Bacteria|Actinobacteria|Coriobacteriia|Coriobacteriales|Coriobacteriaceae|Eggerthella	PTPN2_SeverityTT_Severe	0.00206233	106	59	0.23742382	0.56856758
PTPN2_Severity	Bacteria|Firmicutes|Clostridia|Clostridiales|Unclassified|Unclassified	PTPN2_SeverityTT_Severe	-0.0058485	106	106	0.25028409	0.56857995
PTPN2_Severity	Bacteria|Firmicutes|Clostridia|Clostridiales|Peptostreptococcaceae|	PTPN2_SeverityTT_Severe	-0.0012503	106	57	0.25466233	0.56857995
PTPN2_Severity	Bacteria|Firmicutes|Clostridia|Clostridiales|Veillonellaceae|Phascolarctobacterium	PTPN2_SeverityTT_Severe	-0.0095995	106	84	0.25617338	0.56857995
PTPN2_Severity	Bacteria|Actinobacteria|Coriobacteriia|Coriobacteriales|Coriobacteriaceae|Unclassified	PTPN2_SeverityTT_Severe	-0.0007885	106	37	0.30052015	0.64662772
PTPN2_Severity	Bacteria|Proteobacteria|Betaproteobacteria|Burkholderiales|Alcaligenaceae|Sutterella	PTPN2_SeverityTT_Severe	0.01664672	106	105	0.31596326	0.64662772
PTPN2_Severity	Bacteria|Actinobacteria|Actinobacteria|Actinomycetales|Corynebacteriaceae|Corynebacterium	PTPN2_SeverityTT_Severe	-0.002208	106	62	0.33056082	0.64662772
PTPN2_Severity	Bacteria|Firmicutes|Bacilli|Turicibacterales|Turicibacteraceae|Turicibacter	PTPN2_SeverityTT_Severe	-0.0013856	106	43	0.33333218	0.64662772
PTPN2_Severity	Bacteria|Proteobacteria|Gammaproteobacteria|Enterobacteriales|Enterobacteriaceae|Gluconacetobacter	PTPN2_SeverityTT_Severe	0.00077811	106	34	0.33336797	0.64662772
PTPN2_Severity	Bacteria|Proteobacteria|Gammaproteobacteria|Xanthomonadales|Xanthomonadaceae|	PTPN2_SeverityTT_Severe	-0.0008735	106	33	0.33397256	0.64662772
PTPN2_Severity	Bacteria|Firmicutes|Clostridia|Clostridiales|Veillonellaceae|Dialister	PTPN2_SeverityTT_Severe	-0.0034715	106	68	0.35805227	0.67298332
PTPN2_Severity	Bacteria|Bacteroidetes|Bacteroidia|Bacteroidales|[Barnesiellaceae]|	PTPN2_SeverityTT_Severe	-0.0096814	106	99	0.3661803	0.67298332
PTPN2_Severity	Bacteria|Firmicutes|Clostridia|Clostridiales|Clostridiaceae|	PTPN2_SeverityTT_Severe	-0.001789	106	84	0.37576158	0.67298332
PTPN2_Severity	Bacteria|Firmicutes|Clostridia|Clostridiales|Clostridiaceae|Unclassified	PTPN2_SeverityTT_Severe	-0.0015505	106	79	0.37716648	0.67298332
PTPN2_Severity	Bacteria|Firmicutes|Clostridia|Clostridiales|Veillonellaceae|	PTPN2_SeverityTT_Severe	0.00187702	106	58	0.3874784	0.6780872
PTPN2_Severity	Bacteria|Firmicutes|Bacilli|Lactobacillales|Streptococcaceae|Streptococcus	PTPN2_SeverityTT_Severe	-0.0026664	106	102	0.40696549	0.69875207
PTPN2_Severity	Bacteria|Firmicutes|Clostridia|Clostridiales|Lachnospiraceae|Blautia	PTPN2_SeverityTT_Severe	0.0060434	106	106	0.42739912	0.71237511
PTPN2_Severity	Bacteria|Proteobacteria|Betaproteobacteria|Neisseriales|Neisseriaceae|	PTPN2_SeverityTT_Severe	-0.0007855	106	41	0.43055639	0.71237511
PTPN2_Severity	Bacteria|Proteobacteria|Betaproteobacteria|Burkholderiales|Comamonadaceae|Tepidimonas	PTPN2_SeverityTT_Severe	-0.0006741	106	43	0.4845367	0.75567512
PTPN2_Severity	Bacteria|Verrucomicrobia|Verrucomicrobiae|Verrucomicrobiales|Verrucomicrobiaceae|Akkermansia	PTPN2_SeverityTT_Severe	-0.0056076	106	66	0.48548262	0.75567512
PTPN2_Severity	Bacteria|Firmicutes|Erysipelotrichi|Erysipelotrichales|Erysipelotrichaceae|	PTPN2_SeverityTT_Severe	0.00394943	106	105	0.48601715	0.75567512
PTPN2_Severity	Bacteria|Proteobacteria|Gammaproteobacteria|Enterobacteriales|Enterobacteriaceae|Unclassified	PTPN2_SeverityTT_Severe	0.00218592	106	79	0.48994321	0.75567512
PTPN2_Severity	Bacteria|Firmicutes|Clostridia|Clostridiales|Ruminococcaceae|Oscillospira	PTPN2_SeverityTT_Severe	-0.0030991	106	104	0.51734629	0.77831643
PTPN2_Severity	Bacteria|Firmicutes|Bacilli|Bacillales|Unclassified|Unclassified	PTPN2_SeverityTT_Severe	0.00110251	106	68	0.52995231	0.77831643
PTPN2_Severity	Bacteria|Fusobacteria|Fusobacteriia|Fusobacteriales|Fusobacteriaceae|Fusobacterium	PTPN2_SeverityTT_Severe	-0.0016895	106	91	0.53028152	0.77831643
PTPN2_Severity	Bacteria|Firmicutes|Bacilli|Lactobacillales|Streptococcaceae|Lactococcus	PTPN2_SeverityTT_Severe	-0.000652	106	52	0.56746584	0.81431876
PTPN2_Severity	Bacteria|Proteobacteria|Gammaproteobacteria|Pseudomonadales|Pseudomonadaceae|Pseudomonas	PTPN2_SeverityTT_Severe	0.00033775	106	35	0.57463449	0.81431876
PTPN2_Severity	Bacteria|Firmicutes|Clostridia|Clostridiales|Lachnospiraceae|	PTPN2_SeverityTT_Severe	0.0067576	106	106	0.59011884	0.81431876
PTPN2_Severity	Bacteria|Actinobacteria|Coriobacteriia|Coriobacteriales|Coriobacteriaceae|	PTPN2_SeverityTT_Severe	-0.0006464	106	53	0.59060482	0.81431876
PTPN2_Severity	Bacteria|Bacteroidetes|Bacteroidia|Bacteroidales|Bacteroidaceae|Unclassified	PTPN2_SeverityTT_Severe	-0.0005407	106	58	0.6147615	0.83497458
PTPN2_Severity	Bacteria|Firmicutes|Clostridia|Clostridiales|Veillonellaceae|Unclassified	PTPN2_SeverityTT_Severe	0.00043751	106	51	0.64772807	0.85954711
PTPN2_Severity	Bacteria|Actinobacteria|Actinobacteria|Bifidobacteriales|Bifidobacteriaceae|Bifidobacterium	PTPN2_SeverityTT_Severe	0.00117181	106	90	0.65302996	0.85954711
PTPN2_Severity	Bacteria|Firmicutes|Clostridia|Clostridiales|Clostridiaceae|SMB53	PTPN2_SeverityTT_Severe	0.00025902	106	32	0.66119008	0.85954711
PTPN2_Severity	Bacteria|Firmicutes|Clostridia|Clostridiales|Lachnospiraceae|Lachnospira	PTPN2_SeverityTT_Severe	0.0017861	106	92	0.69202359	0.88695981
PTPN2_Severity	Bacteria|Bacteroidetes|Bacteroidia|Bacteroidales|Porphyromonadaceae|Porphyromonas	PTPN2_SeverityTT_Severe	-0.0002934	106	44	0.72182024	0.88780764
PTPN2_Severity	Bacteria|Proteobacteria|Gammaproteobacteria|Enterobacteriales|Enterobacteriaceae|	PTPN2_SeverityTT_Severe	0.01276778	106	106	0.73221227	0.88780764
PTPN2_Severity	Bacteria|Firmicutes|Clostridia|Clostridiales|Veillonellaceae|Acidaminococcus	PTPN2_SeverityTT_Severe	0.00017924	106	40	0.73911514	0.88780764
PTPN2_Severity	Bacteria|Firmicutes|Clostridia|Clostridiales|Lachnospiraceae|Lachnobacterium	PTPN2_SeverityTT_Severe	-0.0001949	106	37	0.73948611	0.88780764
PTPN2_Severity	Bacteria|Proteobacteria|Gammaproteobacteria|Enterobacteriales|Enterobacteriaceae|Klebsiella	PTPN2_SeverityTT_Severe	-0.0008044	106	55	0.74414865	0.88780764
PTPN2_Severity	Bacteria|Actinobacteria|Coriobacteriia|Coriobacteriales|Coriobacteriaceae|Collinsella	PTPN2_SeverityTT_Severe	-0.001649	106	89	0.75122185	0.88780764
PTPN2_Severity	Bacteria|Firmicutes|Bacilli|Lactobacillales|Enterococcaceae|Enterococcus	PTPN2_SeverityTT_Severe	-0.0002813	106	47	0.7847842	0.91558156
PTPN2_Severity	Bacteria|Tenericutes|Mollicutes|RF39|	PTPN2_SeverityTT_Severe	-0.0001598	106	33	0.83489064	0.95806923
PTPN2_Severity	Bacteria|Actinobacteria|Actinobacteria|Actinomycetales|Micrococcaceae|Rothia	PTPN2_SeverityTT_Severe	0.00021078	106	43	0.84253807	0.95806923
PTPN2_Severity	Bacteria|Proteobacteria|Gammaproteobacteria|Unclassified|Unclassified|Unclassified	PTPN2_SeverityTT_Severe	0.00015105	106	57	0.85725913	0.95806923
PTPN2_Severity	Bacteria|Firmicutes|Bacilli|Bacillales|Staphylococcaceae|Staphylococcus	PTPN2_SeverityTT_Severe	0.00023225	106	67	0.87212072	0.95806923
PTPN2_Severity	Bacteria|Proteobacteria|Gammaproteobacteria|Enterobacteriales|Enterobacteriaceae|Erwinia	PTPN2_SeverityTT_Severe	0.00015095	106	51	0.87384337	0.95806923
PTPN2_Severity	Bacteria|Firmicutes|Bacilli|Gemellales|Gemellaceae|Unclassified	PTPN2_SeverityTT_Severe	-8.98E-05	106	48	0.90045498	0.9754929
PTPN2_Severity	Bacteria|Bacteroidetes|Bacteroidia|Bacteroidales|Prevotellaceae|Prevotella	PTPN2_SeverityTT_Severe	-0.0003049	106	106	0.91176222	0.97612191
PTPN2_Severity	Bacteria|Firmicutes|Clostridia|Clostridiales|Lachnospiraceae|Dorea	PTPN2_SeverityTT_Severe	-0.0002821	106	105	0.92895794	0.97640049
PTPN2_Severity	Bacteria|Firmicutes|Bacilli|Lactobacillales|Lactobacillaceae|Lactobacillus	PTPN2_SeverityTT_Severe	0.00028834	106	88	0.93348179	0.97640049
PTPN2_Severity	Bacteria|Cyanobacteria|4C0d_2|YS2|	PTPN2_SeverityTT_Severe	-7.04E-05	106	32	0.95584879	0.97951512
PTPN2_Severity	Bacteria|Firmicutes|Clostridia|Clostridiales|Lachnospiraceae|Roseburia	PTPN2_SeverityTT_Severe	-0.0003909	106	102	0.9604597	0.97951512
PTPN2_Severity	Bacteria|Bacteroidetes|Bacteroidia|Bacteroidales|S24_7|	PTPN2_SeverityTT_Severe	-0.0003428	106	105	0.96875121	0.97951512
PTPN2_Severity	Bacteria|Actinobacteria|Actinobacteria|Actinomycetales|Actinomycetaceae|Actinomyces	PTPN2_SeverityTT_Severe	2.14E-05	106	57	0.98732266	0.98732266
PTPN2_Severity	Bacteria|Firmicutes|Erysipelotrichi|Erysipelotrichales|Erysipelotrichaceae|Unclassified	PTPN2_SeverityCT_Severe	-0.0064477	38	15	0.01414985	0.42279621
PTPN2_Severity	Bacteria|Proteobacteria|Betaproteobacteria|Neisseriales|Neisseriaceae|Neisseria	PTPN2_SeverityCT_Severe	-0.0050855	38	19	0.01513331	0.42279621
PTPN2_Severity	Bacteria|Bacteroidetes|Bacteroidia|Bacteroidales|[Odoribacteraceae]|Butyricimonas	PTPN2_SeverityCT_Severe	0.01699215	38	17	0.01531203	0.42279621
PTPN2_Severity	Bacteria|Firmicutes|Clostridia|Clostridiales|	PTPN2_SeverityCT_Severe	-0.0502056	38	38	0.0174349	0.42279621
PTPN2_Severity	Bacteria|Proteobacteria|Gammaproteobacteria|Enterobacteriales|Enterobacteriaceae|	PTPN2_SeverityCT_Severe	0.12065262	38	38	0.02973395	0.45889715
PTPN2_Severity	Bacteria|Bacteroidetes|Bacteroidia|Bacteroidales|Unclassified|Unclassified	PTPN2_SeverityCT_Severe	-0.0081761	38	32	0.03077102	0.45889715
PTPN2_Severity	Bacteria|Firmicutes|Clostridia|Clostridiales|Veillonellaceae|Phascolarctobacterium	PTPN2_SeverityCT_Severe	0.05264545	38	34	0.03532098	0.45889715
PTPN2_Severity	Bacteria|Proteobacteria|Gammaproteobacteria|Unclassified|Unclassified|Unclassified	PTPN2_SeverityCT_Severe	0.00336085	38	18	0.04243775	0.45889715
PTPN2_Severity	Bacteria|Firmicutes|Clostridia|Clostridiales|Unclassified|Unclassified	PTPN2_SeverityCT_Severe	-0.0145947	38	38	0.04288205	0.45889715
PTPN2_Severity	Bacteria|Actinobacteria|Coriobacteriia|Coriobacteriales|Coriobacteriaceae|Slackia	PTPN2_SeverityCT_Severe	0.00434397	38	13	0.04730898	0.45889715
PTPN2_Severity	Bacteria|Proteobacteria|Gammaproteobacteria|Enterobacteriales|Enterobacteriaceae|Klebsiella	PTPN2_SeverityCT_Severe	0.00439452	38	19	0.09622078	0.71347789
PTPN2_Severity	Bacteria|Actinobacteria|Coriobacteriia|Coriobacteriales|Coriobacteriaceae|Collinsella	PTPN2_SeverityCT_Severe	0.01974625	38	28	0.09738195	0.71347789
PTPN2_Severity	Bacteria|Firmicutes|Clostridia|Clostridiales|Lachnospiraceae|Coprococcus	PTPN2_SeverityCT_Severe	-0.0163396	38	38	0.09761512	0.71347789
PTPN2_Severity	Bacteria|Firmicutes|Bacilli|Gemellales|Gemellaceae|Unclassified	PTPN2_SeverityCT_Severe	0.00276627	38	23	0.11010726	0.71347789
PTPN2_Severity	Bacteria|Firmicutes|Clostridia|Clostridiales|Lachnospiraceae|[Ruminococcus_	PTPN2_SeverityCT_Severe	0.01982535	38	38	0.12442029	0.71347789
PTPN2_Severity	Bacteria|Firmicutes|Clostridia|Clostridiales|Lachnospiraceae|Epulopiscium	PTPN2_SeverityCT_Severe	0.00338589	38	21	0.12751796	0.71347789
PTPN2_Severity	Bacteria|Bacteroidetes|Bacteroidia|Bacteroidales|[Odoribacteraceae]|Odoribacter	PTPN2_SeverityCT_Severe	0.02296953	38	33	0.14023544	0.71347789
PTPN2_Severity	Bacteria|Proteobacteria|Deltaproteobacteria|Desulfovibrionales|Desulfovibrionaceae|Desulfovibrio	PTPN2_SeverityCT_Severe	-0.0183935	38	20	0.14162524	0.71347789
PTPN2_Severity	Bacteria|Actinobacteria|Actinobacteria|Actinomycetales|Micrococcaceae|Rothia	PTPN2_SeverityCT_Severe	-0.0039996	38	23	0.14599651	0.71347789
PTPN2_Severity	Bacteria|Firmicutes|Clostridia|Clostridiales|[Mogibacteriaceae]|	PTPN2_SeverityCT_Severe	-0.0045211	38	22	0.14710884	0.71347789
PTPN2_Severity	Bacteria|Firmicutes|Clostridia|Clostridiales|[Tissierellaceae]|Anaerococcus	PTPN2_SeverityCT_Severe	-0.0020544	38	15	0.17372544	0.77682595
PTPN2_Severity	Bacteria|Actinobacteria|Coriobacteriia|Coriobacteriales|Coriobacteriaceae|Adlercreutzia	PTPN2_SeverityCT_Severe	-0.003885	38	14	0.17618733	0.77682595
PTPN2_Severity	Bacteria|Firmicutes|Clostridia|Clostridiales|Clostridiaceae|Unclassified	PTPN2_SeverityCT_Severe	0.00280915	38	24	0.19879692	0.8035432
PTPN2_Severity	Bacteria|Firmicutes|Clostridia|Clostridiales|Lachnospiraceae|Blautia	PTPN2_SeverityCT_Severe	0.0273852	38	38	0.2029863	0.8035432
PTPN2_Severity	Bacteria|Bacteroidetes|Bacteroidia|Bacteroidales|Bacteroidaceae|Unclassified	PTPN2_SeverityCT_Severe	-0.0025888	38	17	0.23303378	0.8035432
PTPN2_Severity	Bacteria|Firmicutes|Clostridia|Clostridiales|Lachnospiraceae|Lachnospira	PTPN2_SeverityCT_Severe	-0.0058692	38	28	0.24083211	0.8035432
PTPN2_Severity	Bacteria|Proteobacteria|Betaproteobacteria|Burkholderiales|Comamonadaceae|	PTPN2_SeverityCT_Severe	0.00172368	38	15	0.24482042	0.8035432
PTPN2_Severity	Bacteria|Actinobacteria|Actinobacteria|Actinomycetales|Corynebacteriaceae|Corynebacterium	PTPN2_SeverityCT_Severe	0.00288995	38	24	0.24537512	0.8035432
PTPN2_Severity	Bacteria|Proteobacteria|Betaproteobacteria|Burkholderiales|Oxalobacteraceae|	PTPN2_SeverityCT_Severe	0.00160913	38	13	0.27210838	0.8035432
PTPN2_Severity	Bacteria|Firmicutes|Erysipelotrichi|Erysipelotrichales|Erysipelotrichaceae|[Eubacterium_	PTPN2_SeverityCT_Severe	0.0080414	38	36	0.27588475	0.8035432
PTPN2_Severity	Bacteria|Firmicutes|Clostridia|Clostridiales|Ruminococcaceae|Oscillospira	PTPN2_SeverityCT_Severe	-0.0110043	38	36	0.28302267	0.8035432
PTPN2_Severity	Bacteria|Firmicutes|Clostridia|Clostridiales|Lachnospiraceae|Unclassified	PTPN2_SeverityCT_Severe	-0.0171974	38	38	0.29574414	0.8035432
PTPN2_Severity	Bacteria|Proteobacteria|Deltaproteobacteria|Desulfovibrionales|Desulfovibrionaceae|	PTPN2_SeverityCT_Severe	0.00369363	38	20	0.30010029	0.8035432
PTPN2_Severity	Bacteria|Fusobacteria|Fusobacteriia|Fusobacteriales|Leptotrichiaceae|Leptotrichia	PTPN2_SeverityCT_Severe	-0.0021039	38	16	0.30223866	0.8035432
PTPN2_Severity	Bacteria|Firmicutes|Clostridia|Clostridiales|Ruminococcaceae|Anaerotruncus	PTPN2_SeverityCT_Severe	-0.0023073	38	20	0.31499646	0.8035432
PTPN2_Severity	Bacteria|Firmicutes|Clostridia|Clostridiales|Veillonellaceae|Acidaminococcus	PTPN2_SeverityCT_Severe	-0.0010155	38	12	0.34539997	0.8035432
PTPN2_Severity	Bacteria|Actinobacteria|Actinobacteria|Actinomycetales|Propionibacteriaceae|Propionibacterium	PTPN2_SeverityCT_Severe	-0.0028653	38	21	0.34886991	0.8035432
PTPN2_Severity	Bacteria|Firmicutes|Clostridia|Clostridiales|Ruminococcaceae|Unclassified	PTPN2_SeverityCT_Severe	-0.0122338	38	33	0.35013076	0.8035432
PTPN2_Severity	Bacteria|Firmicutes|Clostridia|Clostridiales|[Tissierellaceae]|Parvimonas	PTPN2_SeverityCT_Severe	-0.0015831	38	12	0.37006868	0.8035432
PTPN2_Severity	Bacteria|Bacteroidetes|Bacteroidia|Bacteroidales|[Barnesiellaceae]|	PTPN2_SeverityCT_Severe	0.0136678	38	36	0.37342849	0.8035432
PTPN2_Severity	Bacteria|Actinobacteria|Coriobacteriia|Coriobacteriales|Coriobacteriaceae|	PTPN2_SeverityCT_Severe	-0.0027027	38	22	0.37588644	0.8035432
PTPN2_Severity	Bacteria|Proteobacteria|Gammaproteobacteria|Enterobacteriales|Enterobacteriaceae|Unclassified	PTPN2_SeverityCT_Severe	0.00410613	38	25	0.3875098	0.8035432
PTPN2_Severity	Bacteria|Bacteroidetes|Bacteroidia|Bacteroidales|Porphyromonadaceae|Parabacteroides	PTPN2_SeverityCT_Severe	0.03186918	38	38	0.39920811	0.8035432
PTPN2_Severity	Bacteria|Firmicutes|Clostridia|Clostridiales|Veillonellaceae|Dialister	PTPN2_SeverityCT_Severe	-0.0020334	38	23	0.40096899	0.8035432
PTPN2_Severity	Bacteria|Firmicutes|Clostridia|Clostridiales|Ruminococcaceae|Ruminococcus	PTPN2_SeverityCT_Severe	-0.0092291	38	38	0.4091297	0.8035432
PTPN2_Severity	Bacteria|Firmicutes|Bacilli|Lactobacillales|Enterococcaceae|Enterococcus	PTPN2_SeverityCT_Severe	-0.0011072	38	12	0.41904278	0.8035432
PTPN2_Severity	Bacteria|Proteobacteria|Gammaproteobacteria|Enterobacteriales|Enterobacteriaceae|Erwinia	PTPN2_SeverityCT_Severe	0.00215028	38	17	0.43539691	0.8035432
PTPN2_Severity	Bacteria|Firmicutes|Clostridia|Clostridiales|Ruminococcaceae|	PTPN2_SeverityCT_Severe	-0.025747	38	38	0.43733367	0.8035432
PTPN2_Severity	Bacteria|Firmicutes|Clostridia|Clostridiales|Lachnospiraceae|Dorea	PTPN2_SeverityCT_Severe	0.00353585	38	36	0.4373448	0.8035432
PTPN2_Severity	Bacteria|Firmicutes|Clostridia|Clostridiales|Veillonellaceae|Unclassified	PTPN2_SeverityCT_Severe	0.00181709	38	21	0.4380797	0.8035432
PTPN2_Severity	Bacteria|Proteobacteria|Gammaproteobacteria|Pasteurellales|Pasteurellaceae|Aggregatibacter	PTPN2_SeverityCT_Severe	-0.0024316	38	12	0.4495741	0.8035432
PTPN2_Severity	Bacteria|Actinobacteria|Coriobacteriia|Coriobacteriales|Coriobacteriaceae|Atopobium	PTPN2_SeverityCT_Severe	-0.0012333	38	20	0.45223769	0.8035432
PTPN2_Severity	Bacteria|Proteobacteria|Gammaproteobacteria|Pasteurellales|Pasteurellaceae|Haemophilus	PTPN2_SeverityCT_Severe	-0.0093284	38	35	0.45351786	0.8035432
PTPN2_Severity	Bacteria|Firmicutes|Erysipelotrichi|Erysipelotrichales|Erysipelotrichaceae|	PTPN2_SeverityCT_Severe	-0.0053791	38	36	0.45678368	0.8035432
PTPN2_Severity	Bacteria|Firmicutes|Erysipelotrichi|Erysipelotrichales|Erysipelotrichaceae|Catenibacterium	PTPN2_SeverityCT_Severe	0.00736366	38	12	0.46232837	0.8035432
PTPN2_Severity	Bacteria|Bacteroidetes|Bacteroidia|Bacteroidales|Prevotellaceae|Prevotella	PTPN2_SeverityCT_Severe	0.02097413	38	38	0.46984194	0.8035432
PTPN2_Severity	Bacteria|Actinobacteria|Actinobacteria|Actinomycetales|Actinomycetaceae|Actinomyces	PTPN2_SeverityCT_Severe	0.00201742	38	30	0.47218518	0.8035432
PTPN2_Severity	Bacteria|Proteobacteria|Alphaproteobacteria|RF32|	PTPN2_SeverityCT_Severe	-0.0039223	38	25	0.49400701	0.81984083
PTPN2_Severity	Bacteria|Firmicutes|Clostridia|Clostridiales|Veillonellaceae|Megasphaera	PTPN2_SeverityCT_Severe	0.00151216	38	20	0.52743831	0.81984083
PTPN2_Severity	Bacteria|Firmicutes|Clostridia|Clostridiales|Ruminococcaceae|Faecalibacterium	PTPN2_SeverityCT_Severe	-0.0272477	38	38	0.54348283	0.81984083
PTPN2_Severity	Bacteria|Bacteroidetes|Bacteroidia|Bacteroidales|[Paraprevotellaceae]|[Prevotella_	PTPN2_SeverityCT_Severe	-0.0014497	38	28	0.54642084	0.81984083
PTPN2_Severity	Bacteria|Proteobacteria|Betaproteobacteria|Burkholderiales|Comamonadaceae|Tepidimonas	PTPN2_SeverityCT_Severe	-0.0007892	38	15	0.55221822	0.81984083
PTPN2_Severity	Bacteria|Firmicutes|Bacilli|Lactobacillales|Streptococcaceae|Lactococcus	PTPN2_SeverityCT_Severe	0.00092842	38	17	0.55538275	0.81984083
PTPN2_Severity	Bacteria|Firmicutes|Clostridia|Clostridiales|Veillonellaceae|	PTPN2_SeverityCT_Severe	0.00149402	38	22	0.56240768	0.81984083
PTPN2_Severity	Bacteria|Verrucomicrobia|Verrucomicrobiae|Verrucomicrobiales|Verrucomicrobiaceae|Akkermansia	PTPN2_SeverityCT_Severe	-0.0039913	38	30	0.56378582	0.81984083
PTPN2_Severity	Bacteria|Proteobacteria|Gammaproteobacteria|Pasteurellales|Pasteurellaceae|Actinobacillus	PTPN2_SeverityCT_Severe	-0.0021044	38	22	0.57274736	0.81984083
PTPN2_Severity	Bacteria|Proteobacteria|Gammaproteobacteria|Pseudomonadales|Moraxellaceae|Acinetobacter	PTPN2_SeverityCT_Severe	-0.0014017	38	23	0.57312747	0.81984083
PTPN2_Severity	Bacteria|Firmicutes|Clostridia|Clostridiales|Lachnospiraceae|Roseburia	PTPN2_SeverityCT_Severe	0.0051666	38	37	0.57473378	0.81984083
PTPN2_Severity	Bacteria|Bacteroidetes|Bacteroidia|Bacteroidales|S24_7|	PTPN2_SeverityCT_Severe	-0.0054122	38	37	0.58596895	0.82375346
PTPN2_Severity	Bacteria|Firmicutes|Clostridia|Clostridiales|Clostridiaceae|Clostridium	PTPN2_SeverityCT_Severe	-0.0014343	38	27	0.62423195	0.84039482
PTPN2_Severity	Bacteria|Tenericutes|Mollicutes|RF39|	PTPN2_SeverityCT_Severe	-0.0004555	38	14	0.63713263	0.84039482
PTPN2_Severity	Bacteria|Firmicutes|Bacilli|Lactobacillales|Streptococcaceae|Streptococcus	PTPN2_SeverityCT_Severe	0.00264319	38	38	0.63790519	0.84039482
PTPN2_Severity	Bacteria|Firmicutes|Bacilli|Lactobacillales|Lactobacillaceae|Lactobacillus	PTPN2_SeverityCT_Severe	0.00289899	38	32	0.63976282	0.84039482
PTPN2_Severity	Bacteria|Cyanobacteria|4C0d_2|YS2|	PTPN2_SeverityCT_Severe	0.0010517	38	17	0.65148267	0.84039482
PTPN2_Severity	Bacteria|Proteobacteria|Betaproteobacteria|Burkholderiales|Alcaligenaceae|Sutterella	PTPN2_SeverityCT_Severe	0.01171753	38	38	0.65352707	0.84039482
PTPN2_Severity	Bacteria|Bacteroidetes|Bacteroidia|Bacteroidales|Porphyromonadaceae|Porphyromonas	PTPN2_SeverityCT_Severe	0.00073618	38	16	0.65845368	0.84039482
PTPN2_Severity	Bacteria|Firmicutes|Clostridia|Clostridiales|Clostridiaceae|	PTPN2_SeverityCT_Severe	0.0021909	38	29	0.67305228	0.84677516
PTPN2_Severity	Bacteria|Firmicutes|Bacilli|Bacillales|Staphylococcaceae|Staphylococcus	PTPN2_SeverityCT_Severe	-0.0011005	38	25	0.68091199	0.84677516
PTPN2_Severity	Bacteria|Bacteroidetes|Bacteroidia|Bacteroidales|Rikenellaceae|Unclassified	PTPN2_SeverityCT_Severe	-0.0004944	38	14	0.70922631	0.87082217
PTPN2_Severity	Bacteria|Proteobacteria|Deltaproteobacteria|Desulfovibrionales|Desulfovibrionaceae|Bilophila	PTPN2_SeverityCT_Severe	-0.0025393	38	25	0.73983034	0.88767043
PTPN2_Severity	Bacteria|Firmicutes|Clostridia|Clostridiales|Lachnospiraceae|Anaerostipes	PTPN2_SeverityCT_Severe	-0.0003144	38	13	0.74125057	0.88767043
PTPN2_Severity	Bacteria|Actinobacteria|Coriobacteriia|Coriobacteriales|Coriobacteriaceae|Eggerthella	PTPN2_SeverityCT_Severe	0.00083891	38	22	0.77308227	0.91449976
PTPN2_Severity	Bacteria|Firmicutes|Clostridia|Clostridiales|Lachnospiraceae|	PTPN2_SeverityCT_Severe	0.00920665	38	38	0.79800363	0.91693946
PTPN2_Severity	Bacteria|Firmicutes|Clostridia|Clostridiales|Christensenellaceae|	PTPN2_SeverityCT_Severe	0.00095394	38	17	0.80051386	0.91693946
PTPN2_Severity	Bacteria|Proteobacteria|Gammaproteobacteria|Enterobacteriales|Enterobacteriaceae|Gluconacetobacter	PTPN2_SeverityCT_Severe	-0.000329	38	12	0.80350365	0.91693946
PTPN2_Severity	Bacteria|Firmicutes|Bacilli|Turicibacterales|Turicibacteraceae|Turicibacter	PTPN2_SeverityCT_Severe	-0.0006199	38	19	0.82536422	0.93093406
PTPN2_Severity	Bacteria|Bacteroidetes|Bacteroidia|Bacteroidales|Rikenellaceae|	PTPN2_SeverityCT_Severe	0.00337613	38	38	0.83853743	0.93492104
PTPN2_Severity	Bacteria|Firmicutes|Clostridia|Clostridiales|Peptostreptococcaceae|	PTPN2_SeverityCT_Severe	0.00032307	38	16	0.85415192	0.94150836
PTPN2_Severity	Bacteria|Actinobacteria|Actinobacteria|Bifidobacteriales|Bifidobacteriaceae|Bifidobacterium	PTPN2_SeverityCT_Severe	-0.0003745	38	24	0.89506067	0.97551556
PTPN2_Severity	Bacteria|Firmicutes|Erysipelotrichi|Erysipelotrichales|Erysipelotrichaceae|Coprobacillus	PTPN2_SeverityCT_Severe	-0.000122	38	13	0.93005941	0.98168926
PTPN2_Severity	Bacteria|Bacteroidetes|Bacteroidia|Bacteroidales|Bacteroidaceae|Bacteroides	PTPN2_SeverityCT_Severe	0.00609659	38	38	0.94454166	0.98168926
PTPN2_Severity	Bacteria|Bacteroidetes|Bacteroidia|Bacteroidales|[Paraprevotellaceae]|Paraprevotella	PTPN2_SeverityCT_Severe	-6.61E-05	38	14	0.94879123	0.98168926
PTPN2_Severity	Bacteria|Bacteroidetes|Bacteroidia|Bacteroidales|	PTPN2_SeverityCT_Severe	6.93E-05	38	19	0.95491702	0.98168926
PTPN2_Severity	Bacteria|Firmicutes|Clostridia|Clostridiales|Lachnospiraceae|Lachnobacterium	PTPN2_SeverityCT_Severe	5.63E-05	38	13	0.95546477	0.98168926
PTPN2_Severity	Bacteria|Fusobacteria|Fusobacteriia|Fusobacteriales|Fusobacteriaceae|Fusobacterium	PTPN2_SeverityCT_Severe	-0.000165	38	33	0.96847982	0.98168926
PTPN2_Severity	Bacteria|Firmicutes|Bacilli|Bacillales|Unclassified|Unclassified	PTPN2_SeverityCT_Severe	-8.38E-05	38	31	0.97156875	0.98168926
PTPN2_Severity	Bacteria|Firmicutes|Clostridia|Clostridiales|Veillonellaceae|Veillonella	PTPN2_SeverityCT_Severe	2.52E-05	38	34	0.99720158	0.99720158
