# Supplementary material for: The presence of genetic risk variants within PTPN2 and PTPN22 is associated with intestinal microbiota alterations in Swiss IBD cohort patients
Source: PLoS One. 2018 Jul 2;13(7):e0199664. doi: 10.1371/journal.pone.0199664 (PMC6028086; doi:10.1371/journal.pone.0199664)
Supplement: S4 Table — Taxonomic difference of PTPN22 variants in CD disease group was identified based on the disease severity status and significant and non-significant differences were recorded based on MaAsLin output file. Table shows coefficient value for each taxa and number of samples that were analyzed. A p-value <0.05 is considered significant. (PDF) [file pone.0199664.s005.pdf]

Suppl. Table 4. Comparison of relative abundances of different disease severities and PTPN22 variants in CD samples using MaAsLin

<table>
<tr><th>Variable</th><th>Feature</th><th>Value</th><th>Coefficient</th><th>N</th><th>N not 0</th><th>P-value</th><th>Q-value</th></tr>
<tr><td>PTPN22_Severity</td><td>Bacteria|Firmicutes|Clostridia|Clostridiales|Ruminococcaceae|Faecalibacterium</td><td>PTPN22_SeverityGG_Severe</td><td>-0.0868204</td><td>126</td><td>122</td><td>0.00011203</td><td>0.01041908</td></tr>
<tr><td>PTPN22_Severity</td><td>Bacteria|Proteobacteria|Deltaproteobacteria|Desulfovibrionales|Desulfovibrionaceae|Bilophila</td><td>PTPN22_SeverityGG_Severe</td><td>-0.0169704</td><td>126</td><td>80</td><td>0.00023266</td><td>0.0108187</td></tr>
<tr><td>PTPN22_Severity</td><td>Bacteria|Firmicutes|Clostridia|Clostridiales|Lachnospiraceae|Coprococcus</td><td>PTPN22_SeverityGG_Severe</td><td>-0.0151982</td><td>126</td><td>124</td><td>0.00085765</td><td>0.02658727</td></tr>
<tr><td>PTPN22_Severity</td><td>Bacteria|Firmicutes|Erysipelotrichi|Erysipelotrichales|Erysipelotrichaceae|Unclassified</td><td>PTPN22_SeverityGG_Severe</td><td>-0.0036982</td><td>126</td><td>47</td><td>0.00144338</td><td>0.03355853</td></tr>
<tr><td>PTPN22_Severity</td><td>Bacteria|Firmicutes|Clostridia|Clostridiales|Veillonellaceae|Megasphaera</td><td>PTPN22_SeverityGG_Severe</td><td>0.00178281</td><td>126</td><td>42</td><td>0.01286464</td><td>0.23928229</td></tr>
<tr><td>PTPN22_Severity</td><td>Bacteria|Firmicutes|Clostridia|Clostridiales|Ruminococcaceae|Unclassified</td><td>PTPN22_SeverityGG_Severe</td><td>-0.0127169</td><td>126</td><td>117</td><td>0.01684908</td><td>0.26116074</td></tr>
<tr><td>PTPN22_Severity</td><td>Bacteria|Firmicutes|Clostridia|Clostridiales|Ruminococcaceae|</td><td>PTPN22_SeverityGG_Severe</td><td>-0.0376544</td><td>126</td><td>126</td><td>0.02109803</td><td>0.28030234</td></tr>
<tr><td>PTPN22_Severity</td><td>Bacteria|Firmicutes|Clostridia|Clostridiales|</td><td>PTPN22_SeverityGG_Severe</td><td>-0.0181595</td><td>126</td><td>126</td><td>0.02664883</td><td>0.29260538</td></tr>
<tr><td>PTPN22_Severity</td><td>Bacteria|Bacteroidetes|Bacteroidia|Bacteroidales|Bacteroidaceae|Bacteroides</td><td>PTPN22_SeverityGG_Severe</td><td>0.09668945</td><td>126</td><td>126</td><td>0.02831665</td><td>0.29260538</td></tr>
<tr><td>PTPN22_Severity</td><td>Bacteria|Firmicutes|Clostridia|Clostridiales|Veillonellaceae|Veillonella</td><td>PTPN22_SeverityGG_Severe</td><td>0.01025761</td><td>126</td><td>104</td><td>0.04639485</td><td>0.3252666</td></tr>
<tr><td>PTPN22_Severity</td><td>Bacteria|Firmicutes|Clostridia|Clostridiales|Clostridiaceae|Clostridium</td><td>PTPN22_SeverityGG_Severe</td><td>-0.0025885</td><td>126</td><td>86</td><td>0.04863614</td><td>0.3252666</td></tr>
<tr><td>PTPN22_Severity</td><td>Bacteria|Firmicutes|Clostridia|Clostridiales|Veillonellaceae|Dialister</td><td>PTPN22_SeverityGG_Severe</td><td>-0.0065119</td><td>126</td><td>77</td><td>0.05537606</td><td>0.3252666</td></tr>
<tr><td>PTPN22_Severity</td><td>Bacteria|Bacteroidetes|Bacteroidia|Bacteroidales|Unclassified|Unclassified</td><td>PTPN22_SeverityGG_Severe</td><td>-0.0042168</td><td>126</td><td>112</td><td>0.0557348</td><td>0.3252666</td></tr>
<tr><td>PTPN22_Severity</td><td>Bacteria|Firmicutes|Erysipelotrichi|Erysipelotrichales|Erysipelotrichaceae|Coprobacillus</td><td>PTPN22_SeverityGG_Severe</td><td>-0.0014443</td><td>126</td><td>38</td><td>0.05741629</td><td>0.3252666</td></tr>
<tr><td>PTPN22_Severity</td><td>Bacteria|Proteobacteria|Deltaproteobacteria|Desulfovibrionales|Desulfovibrionaceae|Desulfovibrio</td><td>PTPN22_SeverityGG_Severe</td><td>-0.0021575</td><td>126</td><td>55</td><td>0.06176417</td><td>0.3252666</td></tr>
<tr><td>PTPN22_Severity</td><td>Bacteria|Actinobacteria|Actinobacteria|Actinomycetales|Propionibacteriaceae|Propionibacterium</td><td>PTPN22_SeverityGG_Severe</td><td>-0.0020922</td><td>126</td><td>69</td><td>0.06268757</td><td>0.3252666</td></tr>
<tr><td>PTPN22_Severity</td><td>Bacteria|Firmicutes|Clostridia|Clostridiales|Ruminococcaceae|Ruminococcus</td><td>PTPN22_SeverityGG_Severe</td><td>-0.011842</td><td>126</td><td>124</td><td>0.0658416</td><td>0.3252666</td></tr>
<tr><td>PTPN22_Severity</td><td>Bacteria|Bacteroidetes|Bacteroidia|Bacteroidales|Rikenellaceae|</td><td>PTPN22_SeverityGG_Severe</td><td>-0.0161002</td><td>126</td><td>123</td><td>0.06618544</td><td>0.3252666</td></tr>
<tr><td>PTPN22_Severity</td><td>Bacteria|Bacteroidetes|Bacteroidia|Bacteroidales|</td><td>PTPN22_SeverityGG_Severe</td><td>-0.0011846</td><td>126</td><td>46</td><td>0.06645232</td><td>0.3252666</td></tr>
<tr><td>PTPN22_Severity</td><td>Bacteria|Firmicutes|Clostridia|Clostridiales|Unclassified|Unclassified</td><td>PTPN22_SeverityGG_Severe</td><td>-0.0080445</td><td>126</td><td>126</td><td>0.0740629</td><td>0.33363962</td></tr>
<tr><td>PTPN22_Severity</td><td>Bacteria|Firmicutes|Clostridia|Clostridiales|Lachnospiraceae|Blautia</td><td>PTPN22_SeverityGG_Severe</td><td>0.01549343</td><td>126</td><td>126</td><td>0.07786873</td><td>0.33363962</td></tr>
<tr><td>PTPN22_Severity</td><td>Bacteria|Bacteroidetes|Bacteroidia|Bacteroidales|[Paraprevotellaceae]|[Prevotella_</td><td>PTPN22_SeverityGG_Severe</td><td>0.00204727</td><td>126</td><td>90</td><td>0.0789255</td><td>0.33363962</td></tr>
<tr><td>PTPN22_Severity</td><td>Bacteria|Proteobacteria|Betaproteobacteria|Neisseriales|Neisseriaceae|Neisseria</td><td>PTPN22_SeverityGG_Severe</td><td>-0.001545</td><td>126</td><td>51</td><td>0.08281922</td><td>0.33487771</td></tr>
<tr><td>PTPN22_Severity</td><td>Bacteria|Firmicutes|Clostridia|Clostridiales|Christensenellaceae|</td><td>PTPN22_SeverityGG_Severe</td><td>-0.0015412</td><td>126</td><td>62</td><td>0.09550571</td><td>0.37008463</td></tr>
<tr><td>PTPN22_Severity</td><td>Bacteria|Firmicutes|Clostridia|Clostridiales|Lachnospiraceae|</td><td>PTPN22_SeverityGG_Severe</td><td>0.02238727</td><td>126</td><td>126</td><td>0.12753557</td><td>0.4442103</td></tr>
<tr><td>PTPN22_Severity</td><td>Bacteria|Firmicutes|Clostridia|Clostridiales|Ruminococcaceae|Anaerotruncus</td><td>PTPN22_SeverityGG_Severe</td><td>-0.0013785</td><td>126</td><td>45</td><td>0.1287423</td><td>0.4442103</td></tr>
<tr><td>PTPN22_Severity</td><td>Bacteria|Firmicutes|Clostridia|Clostridiales|Lachnospiraceae|[Ruminococcus_</td><td>PTPN22_SeverityGG_Severe</td><td>0.01141981</td><td>126</td><td>126</td><td>0.12896428</td><td>0.4442103</td></tr>
<tr><td>PTPN22_Severity</td><td>Bacteria|Actinobacteria|Coriobacteriia|Coriobacteriales|Coriobacteriaceae|Unclassified</td><td>PTPN22_SeverityGG_Severe</td><td>-0.0009932</td><td>126</td><td>41</td><td>0.14404553</td><td>0.47843695</td></tr>
<tr><td>PTPN22_Severity</td><td>Bacteria|Proteobacteria|Alphaproteobacteria|RF32|</td><td>PTPN22_SeverityGG_Severe</td><td>-0.0071988</td><td>126</td><td>73</td><td>0.15170157</td><td>0.48445957</td></tr>
<tr><td>PTPN22_Severity</td><td>Bacteria|Proteobacteria|Gammaproteobacteria|Pseudomonadales|Moraxellaceae|Acinetobacter</td><td>PTPN22_SeverityGG_Severe</td><td>-0.0018874</td><td>126</td><td>66</td><td>0.15627728</td><td>0.48445957</td></tr>
<tr><td>PTPN22_Severity</td><td>Bacteria|Firmicutes|Bacilli|Gemellales|Gemellaceae|Unclassified</td><td>PTPN22_SeverityGG_Severe</td><td>0.00116009</td><td>126</td><td>63</td><td>0.17415524</td><td>0.51603133</td></tr>
<tr><td>PTPN22_Severity</td><td>Bacteria|Firmicutes|Clostridia|Clostridiales|[Mogibacteriaceae]|</td><td>PTPN22_SeverityGG_Severe</td><td>-0.0014003</td><td>126</td><td>52</td><td>0.17755917</td><td>0.51603133</td></tr>
<tr><td>PTPN22_Severity</td><td>Bacteria|Actinobacteria|Coriobacteriia|Coriobacteriales|Coriobacteriaceae|Eggerthella</td><td>PTPN22_SeverityGG_Severe</td><td>0.00198872</td><td>126</td><td>71</td><td>0.21046563</td><td>0.588829</td></tr>
<tr><td>PTPN22_Severity</td><td>Bacteria|Firmicutes|Clostridia|Clostridiales|Lachnospiraceae|Epulopiscium</td><td>PTPN22_SeverityGG_Severe</td><td>-0.0014709</td><td>126</td><td>83</td><td>0.21527082</td><td>0.588829</td></tr>
<tr><td>PTPN22_Severity</td><td>Bacteria|Actinobacteria|Coriobacteriia|Coriobacteriales|Coriobacteriaceae|Adlercreutzia</td><td>PTPN22_SeverityGG_Severe</td><td>-0.0008662</td><td>126</td><td>39</td><td>0.23360189</td><td>0.62071359</td></tr>
<tr><td>PTPN22_Severity</td><td>Bacteria|Actinobacteria|Actinobacteria|Actinomycetales|Micrococcaceae|Rothia</td><td>PTPN22_SeverityGG_Severe</td><td>0.00088964</td><td>126</td><td>53</td><td>0.24176199</td><td>0.62455182</td></tr>
<tr><td>PTPN22_Severity</td><td>Bacteria|Firmicutes|Bacilli|Lactobacillales|Enterococcaceae|Enterococcus</td><td>PTPN22_SeverityGG_Severe</td><td>-0.0008936</td><td>126</td><td>48</td><td>0.25244393</td><td>0.63452124</td></tr>
<tr><td>PTPN22_Severity</td><td>Bacteria|Bacteroidetes|Bacteroidia|Bacteroidales|Bacteroidaceae|Unclassified</td><td>PTPN22_SeverityGG_Severe</td><td>-0.0010781</td><td>126</td><td>65</td><td>0.28864817</td><td>0.70642843</td></tr>
<tr><td>PTPN22_Severity</td><td>Bacteria|Proteobacteria|Gammaproteobacteria|Pasteurellales|Pasteurellaceae|Actinobacillus</td><td>PTPN22_SeverityGG_Severe</td><td>-0.0014701</td><td>126</td><td>70</td><td>0.3057765</td><td>0.7154336</td></tr>
<tr><td>PTPN22_Severity</td><td>Bacteria|Bacteroidetes|Bacteroidia|Bacteroidales|[Odoribacteraceae]|Butyricimonas</td><td>PTPN22_SeverityGG_Severe</td><td>-0.0038198</td><td>126</td><td>54</td><td>0.31406198</td><td>0.7154336</td></tr>
<tr><td>PTPN22_Severity</td><td>Bacteria|Firmicutes|Bacilli|Lactobacillales|Streptococcaceae|Unclassified</td><td>PTPN22_SeverityGG_Severe</td><td>0.00066551</td><td>126</td><td>41</td><td>0.31540621</td><td>0.7154336</td></tr>
<tr><td>PTPN22_Severity</td><td>Bacteria|Bacteroidetes|Bacteroidia|Bacteroidales|Porphyromonadaceae|Parabacteroides</td><td>PTPN22_SeverityGG_Severe</td><td>-0.0163358</td><td>126</td><td>126</td><td>0.33462311</td><td>0.72440786</td></tr>
<tr><td>PTPN22_Severity</td><td>Bacteria|Firmicutes|Clostridia|Clostridiales|Peptostreptococcaceae|</td><td>PTPN22_SeverityGG_Severe</td><td>-0.0008695</td><td>126</td><td>63</td><td>0.34754558</td><td>0.72440786</td></tr>
<tr><td>PTPN22_Severity</td><td>Bacteria|Proteobacteria|Deltaproteobacteria|Desulfovibrionales|Desulfovibrionaceae|</td><td>PTPN22_SeverityGG_Severe</td><td>0.00100285</td><td>126</td><td>40</td><td>0.35021431</td><td>0.72440786</td></tr>
<tr><td>PTPN22_Severity</td><td>Bacteria|Bacteroidetes|Bacteroidia|Bacteroidales|S24_7|</td><td>PTPN22_SeverityGG_Severe</td><td>-0.0052165</td><td>126</td><td>125</td><td>0.36563491</td><td>0.72440786</td></tr>
<tr><td>PTPN22_Severity</td><td>Bacteria|Firmicutes|Clostridia|Clostridiales|Lachnospiraceae|Unclassified</td><td>PTPN22_SeverityGG_Severe</td><td>-0.0077844</td><td>126</td><td>126</td><td>0.36834989</td><td>0.72440786</td></tr>
<tr><td>PTPN22_Severity</td><td>Bacteria|Firmicutes|Clostridia|Clostridiales|Clostridiaceae|Unclassified</td><td>PTPN22_SeverityGG_Severe</td><td>-0.0013734</td><td>126</td><td>92</td><td>0.3724544</td><td>0.72440786</td></tr>
<tr><td>PTPN22_Severity</td><td>Bacteria|Proteobacteria|Gammaproteobacteria|Enterobacteriales|Enterobacteriaceae|Gluconacetobacter</td><td>PTPN22_SeverityGG_Severe</td><td>0.00065441</td><td>126</td><td>38</td><td>0.37514828</td><td>0.72440786</td></tr>
<tr><td>PTPN22_Severity</td><td>Bacteria|Firmicutes|Bacilli|Turicibacterales|Turicibacteraceae|Turicibacter</td><td>PTPN22_SeverityGG_Severe</td><td>-0.0010778</td><td>126</td><td>51</td><td>0.38294661</td><td>0.72440786</td></tr>
<tr><td>PTPN22_Severity</td><td>Bacteria|Proteobacteria|Betaproteobacteria|Neisseriales|Neisseriaceae|</td><td>PTPN22_SeverityGG_Severe</td><td>-0.0007401</td><td>126</td><td>44</td><td>0.41904961</td><td>0.72440786</td></tr>
<tr><td>PTPN22_Severity</td><td>Bacteria|Proteobacteria|Gammaproteobacteria|Unclassified|Unclassified|Unclassified</td><td>PTPN22_SeverityGG_Severe</td><td>0.00069461</td><td>126</td><td>66</td><td>0.42079805</td><td>0.72440786</td></tr>
<tr><td>PTPN22_Severity</td><td>Bacteria|Proteobacteria|Betaproteobacteria|Burkholderiales|Comamonadaceae|</td><td>PTPN22_SeverityGG_Severe</td><td>0.00070751</td><td>126</td><td>40</td><td>0.42120813</td><td>0.72440786</td></tr>
<tr><td>PTPN22_Severity</td><td>Bacteria|Proteobacteria|Gammaproteobacteria|Enterobacteriales|Enterobacteriaceae|Klebsiella</td><td>PTPN22_SeverityGG_Severe</td><td>-0.0014772</td><td>126</td><td>66</td><td>0.4421962</td><td>0.72440786</td></tr>
<tr><td>PTPN22_Severity</td><td>Bacteria|Proteobacteria|Gammaproteobacteria|Xanthomonadales|Xanthomonadaceae|</td><td>PTPN22_SeverityGG_Severe</td><td>-0.0005361</td><td>126</td><td>38</td><td>0.44296159</td><td>0.72440786</td></tr>
<tr><td>PTPN22_Severity</td><td>Bacteria|Proteobacteria|Betaproteobacteria|Burkholderiales|Comamonadaceae|Tepidimonas</td><td>PTPN22_SeverityGG_Severe</td><td>-0.0006126</td><td>126</td><td>52</td><td>0.4453133</td><td>0.72440786</td></tr>
<tr><td>PTPN22_Severity</td><td>Bacteria|Firmicutes|Clostridia|Clostridiales|Lachnospiraceae|Lachnobacterium</td><td>PTPN22_SeverityGG_Severe</td><td>-0.0003944</td><td>126</td><td>40</td><td>0.45146602</td><td>0.72440786</td></tr>
<tr><td>PTPN22_Severity</td><td>Bacteria|Actinobacteria|Actinobacteria|Actinomycetales|Actinomycetaceae|Actinomyces</td><td>PTPN22_SeverityGG_Severe</td><td>0.00092282</td><td>126</td><td>77</td><td>0.45496356</td><td>0.72440786</td></tr>
<tr><td>PTPN22_Severity</td><td>Bacteria|Proteobacteria|Gammaproteobacteria|Enterobacteriales|Enterobacteriaceae|Erwinia</td><td>PTPN22_SeverityGG_Severe</td><td>0.00100048</td><td>126</td><td>61</td><td>0.46275075</td><td>0.72440786</td></tr>
<tr><td>PTPN22_Severity</td><td>Bacteria|Firmicutes|Erysipelotrichi|Erysipelotrichales|Erysipelotrichaceae|[Eubacterium_</td><td>PTPN22_SeverityGG_Severe</td><td>-0.0026284</td><td>126</td><td>118</td><td>0.46349262</td><td>0.72440786</td></tr>
<tr><td>PTPN22_Severity</td><td>Bacteria|Bacteroidetes|Bacteroidia|Bacteroidales|[Odoribacteraceae]|Odoribacter</td><td>PTPN22_SeverityGG_Severe</td><td>-0.0053388</td><td>126</td><td>111</td><td>0.46735991</td><td>0.72440786</td></tr>
<tr><td>PTPN22_Severity</td><td>Bacteria|Firmicutes|Clostridia|Clostridiales|Veillonellaceae|</td><td>PTPN22_SeverityGG_Severe</td><td>0.00136007</td><td>126</td><td>72</td><td>0.49086683</td><td>0.74837074</td></tr>
<tr><td>PTPN22_Severity</td><td>Bacteria|Actinobacteria|Actinobacteria|Actinomycetales|Corynebacteriaceae|Corynebacterium</td><td>PTPN22_SeverityGG_Severe</td><td>-0.001278</td><td>126</td><td>74</td><td>0.49902926</td><td>0.7485439</td></tr>
<tr><td>PTPN22_Severity</td><td>Bacteria|Verrucomicrobia|Verrucomicrobiae|Verrucomicrobiales|Verrucomicrobiaceae|Akkermansia</td><td>PTPN22_SeverityGG_Severe</td><td>-0.0012739</td><td>126</td><td>79</td><td>0.51828419</td><td>0.76508619</td></tr>
<tr><td>PTPN22_Severity</td><td>Bacteria|Proteobacteria|Gammaproteobacteria|Pasteurellales|Pasteurellaceae|Haemophilus</td><td>PTPN22_SeverityGG_Severe</td><td>-0.001761</td><td>126</td><td>118</td><td>0.53686464</td><td>0.78013142</td></tr>
<tr><td>PTPN22_Severity</td><td>Bacteria|Firmicutes|Clostridia|Clostridiales|Clostridiaceae|</td><td>PTPN22_SeverityGG_Severe</td><td>-0.0011971</td><td>126</td><td>98</td><td>0.54717319</td><td>0.78287856</td></tr>
<tr><td>PTPN22_Severity</td><td>Bacteria|Firmicutes|Erysipelotrichi|Erysipelotrichales|Erysipelotrichaceae|Allobaculum</td><td>PTPN22_SeverityGG_Severe</td><td>0.00036794</td><td>126</td><td>42</td><td>0.56252025</td><td>0.79264217</td></tr>
<tr><td>PTPN22_Severity</td><td>Bacteria|Bacteroidetes|Bacteroidia|Bacteroidales|Prevotellaceae|Prevotella</td><td>PTPN22_SeverityGG_Severe</td><td>-0.0013262</td><td>126</td><td>126</td><td>0.59495699</td><td>0.82073687</td></tr>
<tr><td>PTPN22_Severity</td><td>Bacteria|Firmicutes|Bacilli|Bacillales|Staphylococcaceae|Staphylococcus</td><td>PTPN22_SeverityGG_Severe</td><td>-0.0006799</td><td>126</td><td>80</td><td>0.61148651</td><td>0.82073687</td></tr>
<tr><td>PTPN22_Severity</td><td>Bacteria|Bacteroidetes|Bacteroidia|Bacteroidales|[Barnesiellaceae]|</td><td>PTPN22_SeverityGG_Severe</td><td>-0.0048784</td><td>126</td><td>118</td><td>0.61404261</td><td>0.82073687</td></tr>
<tr><td>PTPN22_Severity</td><td>Bacteria|Firmicutes|Bacilli|Bacillales|Unclassified|Unclassified</td><td>PTPN22_SeverityGG_Severe</td><td>0.00076959</td><td>126</td><td>84</td><td>0.62666934</td><td>0.82073687</td></tr>
<tr><td>PTPN22_Severity</td><td>Bacteria|Firmicutes|Bacilli|Lactobacillales|Streptococcaceae|Lactococcus</td><td>PTPN22_SeverityGG_Severe</td><td>-0.0004286</td><td>126</td><td>58</td><td>0.63048289</td><td>0.82073687</td></tr>
<tr><td>PTPN22_Severity</td><td>Bacteria|Proteobacteria|Gammaproteobacteria|Enterobacteriales|Enterobacteriaceae|Unclassified</td><td>PTPN22_SeverityGG_Severe</td><td>0.00136528</td><td>126</td><td>92</td><td>0.63540919</td><td>0.82073687</td></tr>
<tr><td>PTPN22_Severity</td><td>Bacteria|Cyanobacteria|4C0d_2|YS2|</td><td>PTPN22_SeverityGG_Severe</td><td>0.00053667</td><td>126</td><td>42</td><td>0.65055057</td><td>0.82878361</td></tr>
<tr><td>PTPN22_Severity</td><td>Bacteria|Proteobacteria|Betaproteobacteria|Burkholderiales|Alcaligenaceae|Sutterella</td><td>PTPN22_SeverityGG_Severe</td><td>0.00658208</td><td>126</td><td>125</td><td>0.66173034</td><td>0.83163408</td></tr>
<tr><td>PTPN22_Severity</td><td>Bacteria|Firmicutes|Clostridia|Clostridiales|Lachnospiraceae|Lachnospira</td><td>PTPN22_SeverityGG_Severe</td><td>-0.0014539</td><td>126</td><td>105</td><td>0.68133039</td><td>0.84484968</td></tr>
<tr><td>PTPN22_Severity</td><td>Bacteria|Firmicutes|Clostridia|Clostridiales|Ruminococcaceae|Oscillospira</td><td>PTPN22_SeverityGG_Severe</td><td>-0.0018888</td><td>126</td><td>123</td><td>0.69780884</td><td>0.85389766</td></tr>
<tr><td>PTPN22_Severity</td><td>Bacteria|Actinobacteria|Coriobacteriia|Coriobacteriales|Coriobacteriaceae|</td><td>PTPN22_SeverityGG_Severe</td><td>-0.0003928</td><td>126</td><td>64</td><td>0.72753954</td><td>0.87871658</td></tr>
<tr><td>PTPN22_Severity</td><td>Bacteria|Firmicutes|Clostridia|Clostridiales|Veillonellaceae|Unclassified</td><td>PTPN22_SeverityGG_Severe</td><td>0.00045939</td><td>126</td><td>62</td><td>0.78005476</td><td>0.92756329</td></tr>
<tr><td>PTPN22_Severity</td><td>Bacteria|Proteobacteria|Gammaproteobacteria|Enterobacteriales|Enterobacteriaceae|</td><td>PTPN22_SeverityGG_Severe</td><td>0.00860258</td><td>126</td><td>126</td><td>0.7879301</td><td>0.92756329</td></tr>
<tr><td>PTPN22_Severity</td><td>Bacteria|Firmicutes|Clostridia|Clostridiales|Veillonellaceae|Acidaminococcus</td><td>PTPN22_SeverityGG_Severe</td><td>-0.0001263</td><td>126</td><td>44</td><td>0.80236713</td><td>0.93275179</td></tr>
<tr><td>PTPN22_Severity</td><td>Bacteria|Firmicutes|Clostridia|Clostridiales|Lachnospiraceae|Dorea</td><td>PTPN22_SeverityGG_Severe</td><td>0.00067029</td><td>126</td><td>123</td><td>0.82245993</td><td>0.94430585</td></tr>
<tr><td>PTPN22_Severity</td><td>Bacteria|Bacteroidetes|Bacteroidia|Bacteroidales|Porphyromonadaceae|Porphyromonas</td><td>PTPN22_SeverityGG_Severe</td><td>-0.0001569</td><td>126</td><td>51</td><td>0.83889946</td><td>0.95143476</td></tr>
<tr><td>PTPN22_Severity</td><td>Bacteria|Firmicutes|Erysipelotrichi|Erysipelotrichales|Erysipelotrichaceae|</td><td>PTPN22_SeverityGG_Severe</td><td>-0.0007898</td><td>126</td><td>124</td><td>0.88135274</td><td>0.96406151</td></tr>
<tr><td>PTPN22_Severity</td><td>Bacteria|Tenericutes|Mollicutes|RF39|</td><td>PTPN22_SeverityGG_Severe</td><td>-7.26E-05</td><td>126</td><td>41</td><td>0.88869981</td><td>0.96406151</td></tr>
<tr><td>PTPN22_Severity</td><td>Bacteria|Firmicutes|Clostridia|Clostridiales|Veillonellaceae|Phascolarctobacterium</td><td>PTPN22_SeverityGG_Severe</td><td>-0.0010641</td><td>126</td><td>103</td><td>0.89868028</td><td>0.96406151</td></tr>
<tr><td>PTPN22_Severity</td><td>Bacteria|Actinobacteria|Actinobacteria|Bifidobacteriales|Bifidobacteriaceae|Bifidobacterium</td><td>PTPN22_SeverityGG_Severe</td><td>-0.0002715</td><td>126</td><td>102</td><td>0.90731773</td><td>0.96406151</td></tr>
<tr><td>PTPN22_Severity</td><td>Bacteria|Fusobacteria|Fusobacteriia|Fusobacteriales|Fusobacteriaceae|Fusobacterium</td><td>PTPN22_SeverityGG_Severe</td><td>0.00094812</td><td>126</td><td>108</td><td>0.90821336</td><td>0.96406151</td></tr>
<tr><td>PTPN22_Severity</td><td>Bacteria|Firmicutes|Clostridia|Clostridiales|Lachnospiraceae|Roseburia</td><td>PTPN22_SeverityGG_Severe</td><td>-0.0008096</td><td>126</td><td>121</td><td>0.91263596</td><td>0.96406151</td></tr>
<tr><td>PTPN22_Severity</td><td>Bacteria|Firmicutes|Bacilli|Lactobacillales|Lactobacillaceae|Lactobacillus</td><td>PTPN22_SeverityGG_Severe</td><td>0.00032144</td><td>126</td><td>105</td><td>0.9225965</td><td>0.96406151</td></tr>
<tr><td>PTPN22_Severity</td><td>Bacteria|Firmicutes|Unclassified|Unclassified|Unclassified|Unclassified</td><td>PTPN22_SeverityGG_Severe</td><td>-3.06E-05</td><td>126</td><td>42</td><td>0.96858104</td><td>0.98988361</td></tr>
<tr><td>PTPN22_Severity</td><td>Bacteria|Proteobacteria|Gammaproteobacteria|Pseudomonadales|Pseudomonadaceae|Pseudomonas</td><td>PTPN22_SeverityGG_Severe</td><td>-1.66E-05</td><td>126</td><td>40</td><td>0.97665011</td><td>0.98988361</td></tr>
<tr><td>PTPN22_Severity</td><td>Bacteria|Actinobacteria|Coriobacteriia|Coriobacteriales|Coriobacteriaceae|Collinsella</td><td>PTPN22_SeverityGG_Severe</td><td>0.00015022</td><td>126</td><td>103</td><td>0.9792397</td><td>0.98988361</td></tr>
<tr><td>PTPN22_Severity</td><td>Bacteria|Firmicutes|Bacilli|Lactobacillales|Streptococcaceae|Streptococcus</td><td>PTPN22_SeverityGG_Severe</td><td>-2.82E-05</td><td>126</td><td>122</td><td>0.99235982</td><td>0.99235982</td></tr>
<tr><td>PTPN22_Severity</td><td>Bacteria|Firmicutes|Clostridia|Clostridiales|Lachnospiraceae|Lachnobacterium</td><td>PTPN22_SeverityGA_Severe</td><td>0.00497803</td><td>18</td><td>10</td><td>0.009324</td><td>0.67743499</td></tr>
<tr><td>PTPN22_Severity</td><td>Bacteria|Firmicutes|Erysipelotrichi|Erysipelotrichales|Erysipelotrichaceae|Unclassified</td><td>PTPN22_SeverityGA_Severe</td><td>-0.0110185</td><td>18</td><td>9</td><td>0.01315408</td><td>0.67743499</td></tr>
<tr><td>PTPN22_Severity</td><td>Bacteria|Actinobacteria|Actinobacteria|Actinomycetales|Corynebacteriaceae|Corynebacterium</td><td>PTPN22_SeverityGA_Severe</td><td>0.0092857</td><td>18</td><td>12</td><td>0.04163404</td><td>0.98568723</td></tr>
<tr><td>PTPN22_Severity</td><td>Bacteria|Firmicutes|Clostridia|Clostridiales|</td><td>PTPN22_SeverityGA_Severe</td><td>-0.1193731</td><td>18</td><td>18</td><td>0.06791289</td><td>0.98568723</td></tr>
<tr><td>PTPN22_Severity</td><td>Bacteria|Firmicutes|Bacilli|Bacillales|Staphylococcaceae|Staphylococcus</td><td>PTPN22_SeverityGA_Severe</td><td>0.00736251</td><td>18</td><td>12</td><td>0.07490053</td><td>0.98568723</td></tr>
<tr><td>PTPN22_Severity</td><td>Bacteria|Cyanobacteria|Chloroplast|Streptophyta|</td><td>PTPN22_SeverityGA_Severe</td><td>0.00309381</td><td>18</td><td>6</td><td>0.07841649</td><td>0.98568723</td></tr>
<tr><td>PTPN22_Severity</td><td>Bacteria|Actinobacteria|Coriobacteriia|Coriobacteriales|Coriobacteriaceae|</td><td>PTPN22_SeverityGA_Severe</td><td>-0.00623</td><td>18</td><td>11</td><td>0.10317268</td><td>0.98568723</td></tr>
<tr><td>PTPN22_Severity</td><td>Bacteria|Actinobacteria|Coriobacteriia|Coriobacteriales|Coriobacteriaceae|Collinsella</td><td>PTPN22_SeverityGA_Severe</td><td>0.01542932</td><td>18</td><td>14</td><td>0.12343743</td><td>0.98568723</td></tr>
<tr><td>PTPN22_Severity</td><td>Bacteria|Firmicutes|Bacilli|Turicibacterales|Turicibacteraceae|Turicibacter</td><td>PTPN22_SeverityGA_Severe</td><td>0.00754632</td><td>18</td><td>11</td><td>0.12861377</td><td>0.98568723</td></tr>
<tr><td>PTPN22_Severity</td><td>Bacteria|Firmicutes|Clostridia|Clostridiales|Lachnospiraceae|Unclassified</td><td>PTPN22_SeverityGA_Severe</td><td>-0.0305672</td><td>18</td><td>18</td><td>0.13474066</td><td>0.98568723</td></tr>
<tr><td>PTPN22_Severity</td><td>Bacteria|Proteobacteria|Deltaproteobacteria|Desulfovibrionales|Desulfovibrionaceae|Desulfovibrio</td><td>PTPN22_SeverityGA_Severe</td><td>-0.0272751</td><td>18</td><td>11</td><td>0.14632046</td><td>0.98568723</td></tr>
<tr><td>PTPN22_Severity</td><td>Bacteria|Firmicutes|Clostridia|Clostridiales|Veillonellaceae|Selenomonas</td><td>PTPN22_SeverityGA_Severe</td><td>0.00319199</td><td>18</td><td>6</td><td>0.1518327</td><td>0.98568723</td></tr>
<tr><td>PTPN22_Severity</td><td>Bacteria|Proteobacteria|Gammaproteobacteria|Enterobacteriales|Enterobacteriaceae|Unclassified</td><td>PTPN22_SeverityGA_Severe</td><td>0.00774055</td><td>18</td><td>12</td><td>0.15517886</td><td>0.98568723</td></tr>
<tr><td>PTPN22_Severity</td><td>Bacteria|Firmicutes|Bacilli|Lactobacillales|Lactobacillaceae|Lactobacillus</td><td>PTPN22_SeverityGA_Severe</td><td>0.01840661</td><td>18</td><td>15</td><td>0.17539551</td><td>0.98568723</td></tr>
<tr><td>PTPN22_Severity</td><td>Bacteria|Firmicutes|Bacilli|Lactobacillales|Enterococcaceae|Enterococcus</td><td>PTPN22_SeverityGA_Severe</td><td>0.0050924</td><td>18</td><td>11</td><td>0.17967382</td><td>0.98568723</td></tr>
<tr><td>PTPN22_Severity</td><td>Bacteria|Bacteroidetes|Bacteroidia|Bacteroidales|[Paraprevotellaceae]|[Prevotella_</td><td>PTPN22_SeverityGA_Severe</td><td>-0.0240243</td><td>18</td><td>17</td><td>0.18701024</td><td>0.98568723</td></tr>
<tr><td>PTPN22_Severity</td><td>Bacteria|Bacteroidetes|Bacteroidia|Bacteroidales|Porphyromonadaceae|Parabacteroides</td><td>PTPN22_SeverityGA_Severe</td><td>0.04432338</td><td>18</td><td>18</td><td>0.19030122</td><td>0.98568723</td></tr>
<tr><td>PTPN22_Severity</td><td>Bacteria|Firmicutes|Bacilli|Lactobacillales|Unclassified|Unclassified</td><td>PTPN22_SeverityGA_Severe</td><td>0.00266003</td><td>18</td><td>7</td><td>0.21158294</td><td>0.98568723</td></tr>
<tr><td>PTPN22_Severity</td><td>Bacteria|Firmicutes|Clostridia|Clostridiales|Lachnospiraceae|Dorea</td><td>PTPN22_SeverityGA_Severe</td><td>-0.006656</td><td>18</td><td>18</td><td>0.21314374</td><td>0.98568723</td></tr>
<tr><td>PTPN22_Severity</td><td>Bacteria|Firmicutes|Clostridia|Clostridiales|Unclassified|Unclassified</td><td>PTPN22_SeverityGA_Severe</td><td>-0.0185544</td><td>18</td><td>18</td><td>0.21671231</td><td>0.98568723</td></tr>
<tr><td>PTPN22_Severity</td><td>Bacteria|Bacteroidetes|Bacteroidia|Bacteroidales|Prevotellaceae|Prevotella</td><td>PTPN22_SeverityGA_Severe</td><td>-0.0293441</td><td>18</td><td>18</td><td>0.23167277</td><td>0.98568723</td></tr>
<tr><td>PTPN22_Severity</td><td>Bacteria|Firmicutes|Clostridia|Clostridiales|Ruminococcaceae|Oscillospira</td><td>PTPN22_SeverityGA_Severe</td><td>-0.0199444</td><td>18</td><td>17</td><td>0.24437828</td><td>0.98568723</td></tr>
<tr><td>PTPN22_Severity</td><td>Bacteria|Proteobacteria|Gammaproteobacteria|Pasteurellales|Pasteurellaceae|Aggregatibacter</td><td>PTPN22_SeverityGA_Severe</td><td>-0.0080852</td><td>18</td><td>9</td><td>0.24924296</td><td>0.98568723</td></tr>
<tr><td>PTPN22_Severity</td><td>Bacteria|Proteobacteria|Betaproteobacteria|Burkholderiales|Oxalobacteraceae|</td><td>PTPN22_SeverityGA_Severe</td><td>0.00224639</td><td>18</td><td>7</td><td>0.27233563</td><td>0.98568723</td></tr>
<tr><td>PTPN22_Severity</td><td>Bacteria|Proteobacteria|Deltaproteobacteria|Desulfovibrionales|Desulfovibrionaceae|Bilophila</td><td>PTPN22_SeverityGA_Severe</td><td>-0.0123848</td><td>18</td><td>10</td><td>0.27890743</td><td>0.98568723</td></tr>
<tr><td>PTPN22_Severity</td><td>Bacteria|Bacteroidetes|Bacteroidia|Bacteroidales|Porphyromonadaceae|Porphyromonas</td><td>PTPN22_SeverityGA_Severe</td><td>0.00276348</td><td>18</td><td>9</td><td>0.30301009</td><td>0.98568723</td></tr>
<tr><td>PTPN22_Severity</td><td>Bacteria|Firmicutes|Clostridia|Clostridiales|Veillonellaceae|</td><td>PTPN22_SeverityGA_Severe</td><td>-0.0176897</td><td>18</td><td>8</td><td>0.31542512</td><td>0.98568723</td></tr>
<tr><td>PTPN22_Severity</td><td>Bacteria|Firmicutes|Clostridia|Clostridiales|Veillonellaceae|Megasphaera</td><td>PTPN22_SeverityGA_Severe</td><td>-0.0026453</td><td>18</td><td>8</td><td>0.31941739</td><td>0.98568723</td></tr>
<tr><td>PTPN22_Severity</td><td>Bacteria|Fusobacteria|Fusobacteriia|Fusobacteriales|Leptotrichiaceae|Leptotrichia</td><td>PTPN22_SeverityGA_Severe</td><td>0.00179882</td><td>18</td><td>8</td><td>0.32592162</td><td>0.98568723</td></tr>
<tr><td>PTPN22_Severity</td><td>Bacteria|Bacteroidetes|Bacteroidia|Bacteroidales|[Paraprevotellaceae]|Paraprevotella</td><td>PTPN22_SeverityGA_Severe</td><td>0.00151062</td><td>18</td><td>10</td><td>0.34861788</td><td>0.98568723</td></tr>
<tr><td>PTPN22_Severity</td><td>Bacteria|Firmicutes|Clostridia|Clostridiales|Clostridiaceae|Clostridium</td><td>PTPN22_SeverityGA_Severe</td><td>0.00575189</td><td>18</td><td>13</td><td>0.35419863</td><td>0.98568723</td></tr>
<tr><td>PTPN22_Severity</td><td>Bacteria|Actinobacteria|Actinobacteria|Actinomycetales|Propionibacteriaceae|Propionibacterium</td><td>PTPN22_SeverityGA_Severe</td><td>0.00488455</td><td>18</td><td>12</td><td>0.35556809</td><td>0.98568723</td></tr>
<tr><td>PTPN22_Severity</td><td>Bacteria|Firmicutes|Clostridia|Clostridiales|Ruminococcaceae|Unclassified</td><td>PTPN22_SeverityGA_Severe</td><td>-0.0152796</td><td>18</td><td>17</td><td>0.35608274</td><td>0.98568723</td></tr>
<tr><td>PTPN22_Severity</td><td>Bacteria|Firmicutes|Clostridia|Clostridiales|Dehalobacteriaceae|Dehalobacterium</td><td>PTPN22_SeverityGA_Severe</td><td>-0.008059</td><td>18</td><td>6</td><td>0.35814301</td><td>0.98568723</td></tr>
<tr><td>PTPN22_Severity</td><td>Bacteria|Firmicutes|Clostridia|Clostridiales|Peptostreptococcaceae|</td><td>PTPN22_SeverityGA_Severe</td><td>0.00237533</td><td>18</td><td>10</td><td>0.36226076</td><td>0.98568723</td></tr>
<tr><td>PTPN22_Severity</td><td>Bacteria|Firmicutes|Clostridia|Clostridiales|Ruminococcaceae|Ruminococcus</td><td>PTPN22_SeverityGA_Severe</td><td>-0.0146916</td><td>18</td><td>18</td><td>0.36738007</td><td>0.98568723</td></tr>
<tr><td>PTPN22_Severity</td><td>Bacteria|Cyanobacteria|4C0d_2|YS2|</td><td>PTPN22_SeverityGA_Severe</td><td>-0.0025192</td><td>18</td><td>7</td><td>0.37011126</td><td>0.98568723</td></tr>
<tr><td>PTPN22_Severity</td><td>Bacteria|Proteobacteria|Betaproteobacteria|Burkholderiales|Comamonadaceae|</td><td>PTPN22_SeverityGA_Severe</td><td>0.00131958</td><td>18</td><td>6</td><td>0.37458338</td><td>0.98568723</td></tr>
<tr><td>PTPN22_Severity</td><td>Bacteria|Firmicutes|Unclassified|Unclassified|Unclassified|Unclassified</td><td>PTPN22_SeverityGA_Severe</td><td>0.00187446</td><td>18</td><td>8</td><td>0.38021504</td><td>0.98568723</td></tr>
<tr><td>PTPN22_Severity</td><td>Bacteria|Firmicutes|Clostridia|Clostridiales|Lachnospiraceae|[Ruminococcus_</td><td>PTPN22_SeverityGA_Severe</td><td>0.01573289</td><td>18</td><td>18</td><td>0.38442617</td><td>0.98568723</td></tr>
<tr><td>PTPN22_Severity</td><td>Bacteria|Firmicutes|Clostridia|Clostridiales|Lachnospiraceae|Coprococcus</td><td>PTPN22_SeverityGA_Severe</td><td>-0.0128952</td><td>18</td><td>18</td><td>0.39816046</td><td>0.98568723</td></tr>
<tr><td>PTPN22_Severity</td><td>Bacteria|Firmicutes|Bacilli|Gemellales|Gemellaceae|Unclassified</td><td>PTPN22_SeverityGA_Severe</td><td>0.0019389</td><td>18</td><td>8</td><td>0.40982903</td><td>0.98568723</td></tr>
<tr><td>PTPN22_Severity</td><td>Bacteria|Spirochaetes|[Brachyspirae]|[Brachyspirales]|Brachyspiraceae|Brachyspira</td><td>PTPN22_SeverityGA_Severe</td><td>0.00207109</td><td>18</td><td>8</td><td>0.41992756</td><td>0.98568723</td></tr>
<tr><td>PTPN22_Severity</td><td>Bacteria|Bacteroidetes|Bacteroidia|Bacteroidales|Rikenellaceae|</td><td>PTPN22_SeverityGA_Severe</td><td>-0.0243759</td><td>18</td><td>17</td><td>0.4283112</td><td>0.98568723</td></tr>
<tr><td>PTPN22_Severity</td><td>Bacteria|Firmicutes|Clostridia|Clostridiales|[Mogibacteriaceae]|</td><td>PTPN22_SeverityGA_Severe</td><td>-0.0036931</td><td>18</td><td>13</td><td>0.43064005</td><td>0.98568723</td></tr>
<tr><td>PTPN22_Severity</td><td>Bacteria|Bacteroidetes|Bacteroidia|Bacteroidales|Bacteroidaceae|Bacteroides</td><td>PTPN22_SeverityGA_Severe</td><td>0.12080923</td><td>18</td><td>18</td><td>0.45312611</td><td>0.98834715</td></tr>
<tr><td>PTPN22_Severity</td><td>Bacteria|Proteobacteria|Gammaproteobacteria|Enterobacteriales|Enterobacteriaceae|Klebsiella</td><td>PTPN22_SeverityGA_Severe</td><td>0.00646002</td><td>18</td><td>8</td><td>0.45435519</td><td>0.98834715</td></tr>
<tr><td>PTPN22_Severity</td><td>Bacteria|Firmicutes|Bacilli|Lactobacillales|Carnobacteriaceae|Granulicatella</td><td>PTPN22_SeverityGA_Severe</td><td>-0.0015333</td><td>18</td><td>7</td><td>0.46058896</td><td>0.98834715</td></tr>
<tr><td>PTPN22_Severity</td><td>Bacteria|Verrucomicrobia|Verrucomicrobiae|Verrucomicrobiales|Verrucomicrobiaceae|Akkermansia</td><td>PTPN22_SeverityGA_Severe</td><td>0.01500467</td><td>18</td><td>17</td><td>0.48709976</td><td>0.99440335</td></tr>
<tr><td>PTPN22_Severity</td><td>Bacteria|Bacteroidetes|Bacteroidia|Bacteroidales|S24_7|</td><td>PTPN22_SeverityGA_Severe</td><td>0.01820599</td><td>18</td><td>17</td><td>0.49156975</td><td>0.99440335</td></tr>
<tr><td>PTPN22_Severity</td><td>Bacteria|Firmicutes|Bacilli|Bacillales|Unclassified|Unclassified</td><td>PTPN22_SeverityGA_Severe</td><td>0.00308122</td><td>18</td><td>15</td><td>0.50016945</td><td>0.99440335</td></tr>
<tr><td>PTPN22_Severity</td><td>Bacteria|Proteobacteria|Gammaproteobacteria|Pasteurellales|Pasteurellaceae|Haemophilus</td><td>PTPN22_SeverityGA_Severe</td><td>-0.014062</td><td>18</td><td>16</td><td>0.55359572</td><td>0.99440335</td></tr>
<tr><td>PTPN22_Severity</td><td>Bacteria|Firmicutes|Clostridia|Clostridiales|Veillonellaceae|Veillonella</td><td>PTPN22_SeverityGA_Severe</td><td>-0.0043406</td><td>18</td><td>16</td><td>0.56293337</td><td>0.99440335</td></tr>
<tr><td>PTPN22_Severity</td><td>Bacteria|Firmicutes|Clostridia|Clostridiales|Ruminococcaceae|Faecalibacterium</td><td>PTPN22_SeverityGA_Severe</td><td>-0.0376687</td><td>18</td><td>18</td><td>0.56466426</td><td>0.99440335</td></tr>
<tr><td>PTPN22_Severity</td><td>Bacteria|Proteobacteria|Gammaproteobacteria|Pseudomonadales|Pseudomonadaceae|Pseudomonas</td><td>PTPN22_SeverityGA_Severe</td><td>0.00104048</td><td>18</td><td>6</td><td>0.57096767</td><td>0.99440335</td></tr>
<tr><td>PTPN22_Severity</td><td>Bacteria|Firmicutes|Clostridia|Clostridiales|Lachnospiraceae|Anaerostipes</td><td>PTPN22_SeverityGA_Severe</td><td>-0.0014576</td><td>18</td><td>9</td><td>0.57410292</td><td>0.99440335</td></tr>
<tr><td>PTPN22_Severity</td><td>Bacteria|Proteobacteria|Gammaproteobacteria|Pasteurellales|Pasteurellaceae|Actinobacillus</td><td>PTPN22_SeverityGA_Severe</td><td>0.00324628</td><td>18</td><td>13</td><td>0.58347557</td><td>0.99440335</td></tr>
<tr><td>PTPN22_Severity</td><td>Bacteria|Proteobacteria|Betaproteobacteria|Burkholderiales|Comamonadaceae|Tepidimonas</td><td>PTPN22_SeverityGA_Severe</td><td>-0.0007745</td><td>18</td><td>6</td><td>0.58854496</td><td>0.99440335</td></tr>
<tr><td>PTPN22_Severity</td><td>Bacteria|Firmicutes|Clostridia|Clostridiales|Clostridiaceae|</td><td>PTPN22_SeverityGA_Severe</td><td>0.00246257</td><td>18</td><td>15</td><td>0.59782481</td><td>0.99440335</td></tr>
<tr><td>PTPN22_Severity</td><td>Bacteria|Proteobacteria|Betaproteobacteria|Neisseriales|Neisseriaceae|Neisseria</td><td>PTPN22_SeverityGA_Severe</td><td>-0.0014209</td><td>18</td><td>14</td><td>0.60706746</td><td>0.99440335</td></tr>
<tr><td>PTPN22_Severity</td><td>Bacteria|Firmicutes|Erysipelotrichi|Erysipelotrichales|Erysipelotrichaceae|Allobaculum</td><td>PTPN22_SeverityGA_Severe</td><td>0.001214</td><td>18</td><td>7</td><td>0.60746178</td><td>0.99440335</td></tr>
<tr><td>PTPN22_Severity</td><td>Bacteria|Firmicutes|Clostridia|Clostridiales|Veillonellaceae|Unclassified</td><td>PTPN22_SeverityGA_Severe</td><td>-0.0015741</td><td>18</td><td>10</td><td>0.61054144</td><td>0.99440335</td></tr>
<tr><td>PTPN22_Severity</td><td>Bacteria|Firmicutes|Clostridia|Clostridiales|Veillonellaceae|Acidaminococcus</td><td>PTPN22_SeverityGA_Severe</td><td>0.00092948</td><td>18</td><td>8</td><td>0.61057765</td><td>0.99440335</td></tr>
<tr><td>PTPN22_Severity</td><td>Bacteria|Fusobacteria|Fusobacteriia|Fusobacteriales|Fusobacteriaceae|Fusobacterium</td><td>PTPN22_SeverityGA_Severe</td><td>-0.0057393</td><td>18</td><td>16</td><td>0.6452716</td><td>0.99440335</td></tr>
<tr><td>PTPN22_Severity</td><td>Bacteria|Actinobacteria|Actinobacteria|Actinomycetales|Micrococcaceae|Rothia</td><td>PTPN22_SeverityGA_Severe</td><td>-0.0017848</td><td>18</td><td>13</td><td>0.66458528</td><td>0.99440335</td></tr>
<tr><td>PTPN22_Severity</td><td>Bacteria|Firmicutes|Erysipelotrichi|Erysipelotrichales|Erysipelotrichaceae|[Eubacterium_</td><td>PTPN22_SeverityGA_Severe</td><td>-0.0034964</td><td>18</td><td>16</td><td>0.67097207</td><td>0.99440335</td></tr>
<tr><td>PTPN22_Severity</td><td>Bacteria|Actinobacteria|Actinobacteria|Bifidobacteriales|Bifidobacteriaceae|Bifidobacterium</td><td>PTPN22_SeverityGA_Severe</td><td>-0.0031055</td><td>18</td><td>12</td><td>0.71519695</td><td>0.99440335</td></tr>
<tr><td>PTPN22_Severity</td><td>Bacteria|Bacteroidetes|Bacteroidia|Bacteroidales|</td><td>PTPN22_SeverityGA_Severe</td><td>-0.0007093</td><td>18</td><td>9</td><td>0.72324257</td><td>0.99440335</td></tr>
<tr><td>PTPN22_Severity</td><td>Bacteria|Bacteroidetes|Bacteroidia|Bacteroidales|Unclassified|Unclassified</td><td>PTPN22_SeverityGA_Severe</td><td>-0.0023871</td><td>18</td><td>16</td><td>0.72556379</td><td>0.99440335</td></tr>
<tr><td>PTPN22_Severity</td><td>Bacteria|Firmicutes|Clostridia|Clostridiales|Lachnospiraceae|Epulopiscium</td><td>PTPN22_SeverityGA_Severe</td><td>-0.0006766</td><td>18</td><td>8</td><td>0.73902959</td><td>0.99440335</td></tr>
<tr><td>PTPN22_Severity</td><td>Bacteria|Proteobacteria|Betaproteobacteria|Burkholderiales|Alcaligenaceae|Sutterella</td><td>PTPN22_SeverityGA_Severe</td><td>0.01314112</td><td>18</td><td>18</td><td>0.74789458</td><td>0.99440335</td></tr>
<tr><td>PTPN22_Severity</td><td>Bacteria|Actinobacteria|Coriobacteriia|Coriobacteriales|Coriobacteriaceae|Atopobium</td><td>PTPN22_SeverityGA_Severe</td><td>-0.0009942</td><td>18</td><td>8</td><td>0.74940308</td><td>0.99440335</td></tr>
<tr><td>PTPN22_Severity</td><td>Bacteria|Proteobacteria|Alphaproteobacteria|RF32|</td><td>PTPN22_SeverityGA_Severe</td><td>0.0043173</td><td>18</td><td>16</td><td>0.75477216</td><td>0.99440335</td></tr>
<tr><td>PTPN22_Severity</td><td>Bacteria|Firmicutes|Erysipelotrichi|Erysipelotrichales|Erysipelotrichaceae|</td><td>PTPN22_SeverityGA_Severe</td><td>0.00356589</td><td>18</td><td>17</td><td>0.75952143</td><td>0.99440335</td></tr>
<tr><td>PTPN22_Severity</td><td>Bacteria|Firmicutes|Clostridia|Clostridiales|Ruminococcaceae|</td><td>PTPN22_SeverityGA_Severe</td><td>-0.0108886</td><td>18</td><td>18</td><td>0.7694758</td><td>0.99440335</td></tr>
<tr><td>PTPN22_Severity</td><td>Bacteria|Firmicutes|Bacilli|Lactobacillales|Streptococcaceae|Streptococcus</td><td>PTPN22_SeverityGA_Severe</td><td>0.0026722</td><td>18</td><td>18</td><td>0.77911703</td><td>0.99440335</td></tr>
<tr><td>PTPN22_Severity</td><td>Bacteria|Tenericutes|Mollicutes|RF39|</td><td>PTPN22_SeverityGA_Severe</td><td>0.00074998</td><td>18</td><td>6</td><td>0.78444423</td><td>0.99440335</td></tr>
<tr><td>PTPN22_Severity</td><td>Bacteria|Firmicutes|Erysipelotrichi|Erysipelotrichales|Erysipelotrichaceae|Coprobacillus</td><td>PTPN22_SeverityGA_Severe</td><td>0.00038933</td><td>18</td><td>7</td><td>0.82589953</td><td>0.99440335</td></tr>
<tr><td>PTPN22_Severity</td><td>Bacteria|Proteobacteria|Gammaproteobacteria|Enterobacteriales|Enterobacteriaceae|Erwinia</td><td>PTPN22_SeverityGA_Severe</td><td>-0.0005204</td><td>18</td><td>7</td><td>0.8262402</td><td>0.99440335</td></tr>
<tr><td>PTPN22_Severity</td><td>Bacteria|Firmicutes|Bacilli|Lactobacillales|Streptococcaceae|Lactococcus</td><td>PTPN22_SeverityGA_Severe</td><td>0.00125437</td><td>18</td><td>11</td><td>0.82806485</td><td>0.99440335</td></tr>
<tr><td>PTPN22_Severity</td><td>Bacteria|Proteobacteria|Gammaproteobacteria|Unclassified|Unclassified|Unclassified</td><td>PTPN22_SeverityGA_Severe</td><td>0.00035653</td><td>18</td><td>9</td><td>0.83172853</td><td>0.99440335</td></tr>
<tr><td>PTPN22_Severity</td><td>Bacteria|Bacteroidetes|Bacteroidia|Bacteroidales|[Odoribacteraceae]|Odoribacter</td><td>PTPN22_SeverityGA_Severe</td><td>-0.0038803</td><td>18</td><td>13</td><td>0.83537472</td><td>0.99440335</td></tr>
<tr><td>PTPN22_Severity</td><td>Bacteria|Firmicutes|Clostridia|Clostridiales|Lachnospiraceae|Lachnospira</td><td>PTPN22_SeverityGA_Severe</td><td>0.00242372</td><td>18</td><td>15</td><td>0.8421673</td><td>0.99440335</td></tr>
<tr><td>PTPN22_Severity</td><td>Bacteria|Actinobacteria|Actinobacteria|Actinomycetales|Actinomycetaceae|Actinomyces</td><td>PTPN22_SeverityGA_Severe</td><td>0.00091984</td><td>18</td><td>10</td><td>0.84702024</td><td>0.99440335</td></tr>
<tr><td>PTPN22_Severity</td><td>Bacteria|Firmicutes|Bacilli|Bacillales|Planococcaceae|Rummeliibacillus</td><td>PTPN22_SeverityGA_Severe</td><td>0.00050026</td><td>18</td><td>6</td><td>0.85813662</td><td>0.99440335</td></tr>
<tr><td>PTPN22_Severity</td><td>Bacteria|Firmicutes|Clostridia|Clostridiales|Clostridiaceae|Unclassified</td><td>PTPN22_SeverityGA_Severe</td><td>-0.0008182</td><td>18</td><td>11</td><td>0.88030827</td><td>0.99440335</td></tr>
<tr><td>PTPN22_Severity</td><td>Bacteria|Proteobacteria|Deltaproteobacteria|Desulfovibrionales|Desulfovibrionaceae|</td><td>PTPN22_SeverityGA_Severe</td><td>0.00075453</td><td>18</td><td>11</td><td>0.88068322</td><td>0.99440335</td></tr>
<tr><td>PTPN22_Severity</td><td>Bacteria|Firmicutes|Clostridia|Clostridiales|[Tissierellaceae]|Anaerococcus</td><td>PTPN22_SeverityGA_Severe</td><td>0.00045562</td><td>18</td><td>9</td><td>0.88114996</td><td>0.99440335</td></tr>
<tr><td>PTPN22_Severity</td><td>Bacteria|Actinobacteria|Coriobacteriia|Coriobacteriales|Coriobacteriaceae|Eggerthella</td><td>PTPN22_SeverityGA_Severe</td><td>-0.0007502</td><td>18</td><td>10</td><td>0.88128412</td><td>0.99440335</td></tr>
<tr><td>PTPN22_Severity</td><td>Bacteria|Firmicutes|Clostridia|Clostridiales|Ruminococcaceae|Anaerotruncus</td><td>PTPN22_SeverityGA_Severe</td><td>-0.0004616</td><td>18</td><td>12</td><td>0.88871803</td><td>0.99440335</td></tr>
<tr><td>PTPN22_Severity</td><td>Bacteria|Firmicutes|Clostridia|Clostridiales|Lachnospiraceae|</td><td>PTPN22_SeverityGA_Severe</td><td>0.00702798</td><td>18</td><td>18</td><td>0.89732116</td><td>0.99440335</td></tr>
<tr><td>PTPN22_Severity</td><td>Bacteria|Actinobacteria|Actinobacteria|Actinomycetales|Micrococcaceae|Micrococcus</td><td>PTPN22_SeverityGA_Severe</td><td>-0.0003284</td><td>18</td><td>8</td><td>0.91041183</td><td>0.99440335</td></tr>
<tr><td>PTPN22_Severity</td><td>Bacteria|Firmicutes|Clostridia|Clostridiales|Christensenellaceae|</td><td>PTPN22_SeverityGA_Severe</td><td>-0.0001556</td><td>18</td><td>7</td><td>0.91088959</td><td>0.99440335</td></tr>
<tr><td>PTPN22_Severity</td><td>Bacteria|Firmicutes|Clostridia|Clostridiales|Lachnospiraceae|Roseburia</td><td>PTPN22_SeverityGA_Severe</td><td>-0.0011903</td><td>18</td><td>18</td><td>0.91201679</td><td>0.99440335</td></tr>
<tr><td>PTPN22_Severity</td><td>Bacteria|Bacteroidetes|Bacteroidia|Bacteroidales|[Barnesiellaceae]|</td><td>PTPN22_SeverityGA_Severe</td><td>-0.0024618</td><td>18</td><td>17</td><td>0.92146088</td><td>0.99440335</td></tr>
<tr><td>PTPN22_Severity</td><td>Bacteria|Firmicutes|Clostridia|Clostridiales|Veillonellaceae|Dialister</td><td>PTPN22_SeverityGA_Severe</td><td>0.00122338</td><td>18</td><td>14</td><td>0.92782991</td><td>0.99440335</td></tr>
<tr><td>PTPN22_Severity</td><td>Bacteria|Firmicutes|Clostridia|Clostridiales|Veillonellaceae|Phascolarctobacterium</td><td>PTPN22_SeverityGA_Severe</td><td>-0.0014415</td><td>18</td><td>15</td><td>0.93647694</td><td>0.99440335</td></tr>
<tr><td>PTPN22_Severity</td><td>Bacteria|Proteobacteria|Gammaproteobacteria|Enterobacteriales|Enterobacteriaceae|</td><td>PTPN22_SeverityGA_Severe</td><td>0.0007559</td><td>18</td><td>18</td><td>0.9680343</td><td>0.9963165</td></tr>
<tr><td>PTPN22_Severity</td><td>Bacteria|Bacteroidetes|Bacteroidia|Bacteroidales|Bacteroidaceae|Unclassified</td><td>PTPN22_SeverityGA_Severe</td><td>8.43E-05</td><td>18</td><td>10</td><td>0.98079865</td><td>0.9963165</td></tr>
<tr><td>PTPN22_Severity</td><td>Bacteria|Firmicutes|Clostridia|Clostridiales|Lachnospiraceae|Blautia</td><td>PTPN22_SeverityGA_Severe</td><td>-0.0004045</td><td>18</td><td>18</td><td>0.98113437</td><td>0.9963165</td></tr>
<tr><td>PTPN22_Severity</td><td>Bacteria|Proteobacteria|Gammaproteobacteria|Enterobacteriales|Enterobacteriaceae|Gluconacetobacter</td><td>PTPN22_SeverityGA_Severe</td><td>4.39E-05</td><td>18</td><td>8</td><td>0.98280175</td><td>0.9963165</td></tr>
<tr><td>PTPN22_Severity</td><td>Bacteria|Proteobacteria|Gammaproteobacteria|Pseudomonadales|Moraxellaceae|Acinetobacter</td><td>PTPN22_SeverityGA_Severe</td><td>5.97E-05</td><td>18</td><td>9</td><td>0.98754926</td><td>0.9963165</td></tr>
<tr><td>PTPN22_Severity</td><td>Bacteria|Proteobacteria|Betaproteobacteria|Neisseriales|Neisseriaceae|</td><td>PTPN22_SeverityGA_Severe</td><td>1.72E-05</td><td>18</td><td>7</td><td>0.9963165</td><td>0.9963165</td></tr>
</table>
