# Supplementary material for: The presence of genetic risk variants within PTPN2 and PTPN22 is associated with intestinal microbiota alterations in Swiss IBD cohort patients
Source: PLoS One. 2018 Jul 2;13(7):e0199664. doi: 10.1371/journal.pone.0199664 (PMC6028086; doi:10.1371/journal.pone.0199664)
Supplement: S6 Table — Taxonomic difference of PTPN22 variants in UC disease group was identified based on the disease severity status and significant and non-significant differences were recorded based on MaAsLin output file. Table shows coefficient value for each taxa and number of samples that were analyzed. A p-value <0.05 is considered significant. (PDF) [file pone.0199664.s007.pdf]

Suppl. Table 6. Comparison of relative abundances of different disease severities and PTPN22 variants in UC samples using MaAsLin

Variable	Feature	Value	Coefficient	N	N not 0	P-value
PTPN22_Severity	Bacteria|Firmicutes|Clostridia|Clostridiales|Lachnospiraceae|Lachnobacterium	PTPN22_SeverityGG_Severe	-0.0098428	133	81	1.29E-05
PTPN22_Severity	Bacteria|Firmicutes|Clostridia|Clostridiales|Lachnospiraceae|Dorea	PTPN22_SeverityGG_Severe	0.00770113	133	129	0.00075014
PTPN22_Severity	Bacteria|Firmicutes|Clostridia|Clostridiales|Ruminococcaceae|Faecalibacterium	PTPN22_SeverityGG_Severe	-0.0599651	133	133	0.00789062
PTPN22_Severity	Bacteria|Proteobacteria|Gammaproteobacteria|Enterobacteriales|Enterobacteriaceae|Klebsiella	PTPN22_SeverityGG_Severe	0.00206065	133	44	0.0096676
PTPN22_Severity	Bacteria|Proteobacteria|Gammaproteobacteria|Enterobacteriales|Enterobacteriaceae|	PTPN22_SeverityGG_Severe	0.01798202	133	125	0.01470432
PTPN22_Severity	Bacteria|Proteobacteria|Gammaproteobacteria|Enterobacteriales|Enterobacteriaceae|Unclassified	PTPN22_SeverityGG_Severe	0.00274026	133	65	0.01479028
PTPN22_Severity	Bacteria|Actinobacteria|Actinobacteria|Bifidobacteriales|Bifidobacteriaceae|Bifidobacterium	PTPN22_SeverityGG_Severe	0.01258345	133	111	0.01633312
PTPN22_Severity	Bacteria|Firmicutes|Erysipelotrichi|Erysipelotrichales|Erysipelotrichaceae|Catenibacterium	PTPN22_SeverityGG_Severe	0.00908501	133	43	0.05043052
PTPN22_Severity	Bacteria|Fusobacteria|Fusobacteriia|Fusobacteriales|Fusobacteriaceae|Fusobacterium	PTPN22_SeverityGG_Severe	0.00595315	133	111	0.0526717
PTPN22_Severity	Bacteria|Verrucomicrobia|Verrucomicrobiae|Verrucomicrobiales|Verrucomicrobiaceae|Akkermansia	PTPN22_SeverityGG_Severe	-0.0123664	133	104	0.05979842
PTPN22_Severity	Bacteria|Actinobacteria|Actinobacteria|Actinomycetales|Micrococcaceae|Rothia	PTPN22_SeverityGG_Severe	0.00238002	133	62	0.06065486
PTPN22_Severity	Bacteria|Proteobacteria|Betaproteobacteria|Neisseriales|Neisseriaceae|Neisseria	PTPN22_SeverityGG_Severe	0.00229071	133	65	0.06813014
PTPN22_Severity	Bacteria|Firmicutes|Clostridia|Clostridiales|Lachnospiraceae|Roseburia	PTPN22_SeverityGG_Severe	0.01354352	133	133	0.08922931
PTPN22_Severity	Bacteria|Bacteroidetes|Bacteroidia|Bacteroidales|[Paraprevotellaceae]|[Prevotella_	PTPN22_SeverityGG_Severe	0.00268796	133	97	0.09678241
PTPN22_Severity	Bacteria|Firmicutes|Bacilli|Lactobacillales|Enterococcaceae|Enterococcus	PTPN22_SeverityGG_Severe	0.00153356	133	47	0.10058367
PTPN22_Severity	Bacteria|Actinobacteria|Coriobacteriia|Coriobacteriales|Coriobacteriaceae|Eggerthella	PTPN22_SeverityGG_Severe	-0.0020574	133	58	0.11114634
PTPN22_Severity	Bacteria|Bacteroidetes|Bacteroidia|Bacteroidales|Porphyromonadaceae|Porphyromonas	PTPN22_SeverityGG_Severe	0.00184356	133	48	0.13360293
PTPN22_Severity	Bacteria|Firmicutes|Erysipelotrichi|Erysipelotrichales|Erysipelotrichaceae|Coprobacillus	PTPN22_SeverityGG_Severe	-0.0018122	133	60	0.13643938
PTPN22_Severity	Bacteria|Firmicutes|Clostridia|Clostridiales|Clostridiaceae|	PTPN22_SeverityGG_Severe	-0.0049817	133	112	0.14125515
PTPN22_Severity	Bacteria|Actinobacteria|Coriobacteriia|Coriobacteriales|Coriobacteriaceae|Collinsella	PTPN22_SeverityGG_Severe	0.00685625	133	119	0.14781666
PTPN22_Severity	Bacteria|Proteobacteria|Deltaproteobacteria|Desulfovibrionales|Desulfovibrionaceae|Desulfovibrio	PTPN22_SeverityGG_Severe	-0.0016057	133	63	0.15016968
PTPN22_Severity	Bacteria|Firmicutes|Bacilli|Bacillales|Unclassified|Unclassified	PTPN22_SeverityGG_Severe	0.00190981	133	84	0.15286994
PTPN22_Severity	Bacteria|Bacteroidetes|Bacteroidia|Bacteroidales|[Odoribacteraceae]|Butyricimonas	PTPN22_SeverityGG_Severe	-0.004486	133	84	0.19317224
PTPN22_Severity	Bacteria|Bacteroidetes|Bacteroidia|Bacteroidales|S24_7|	PTPN22_SeverityGG_Severe	-0.0092588	133	129	0.19734487
PTPN22_Severity	Bacteria|Firmicutes|Clostridia|Clostridiales|Veillonellaceae|Megasphaera	PTPN22_SeverityGG_Severe	0.00129799	133	44	0.20740389
PTPN22_Severity	Bacteria|Firmicutes|Clostridia|Clostridiales|Veillonellaceae|Unclassified	PTPN22_SeverityGG_Severe	0.00136814	133	62	0.21910414
PTPN22_Severity	Bacteria|Proteobacteria|Gammaproteobacteria|Enterobacteriales|Enterobacteriaceae|Gluconacetobacter	PTPN22_SeverityGG_Severe	0.00140866	133	44	0.22220114
PTPN22_Severity	Bacteria|Proteobacteria|Betaproteobacteria|Burkholderiales|Comamonadaceae|	PTPN22_SeverityGG_Severe	-0.0013718	133	51	0.22728
PTPN22_Severity	Bacteria|Firmicutes|Bacilli|Lactobacillales|Streptococcaceae|Streptococcus	PTPN22_SeverityGG_Severe	0.00411794	133	130	0.22818761
PTPN22_Severity	Bacteria|Proteobacteria|Gammaproteobacteria|Pseudomonadales|Moraxellaceae|Acinetobacter	PTPN22_SeverityGG_Severe	0.00119455	133	58	0.25240308
PTPN22_Severity	Bacteria|Firmicutes|Clostridia|Clostridiales|[Tissierellaceae]|Anaerococcus	PTPN22_SeverityGG_Severe	0.00082177	133	41	0.28169395
PTPN22_Severity	Bacteria|Firmicutes|Clostridia|Clostridiales|Veillonellaceae|	PTPN22_SeverityGG_Severe	0.00102496	133	48	0.28368391
PTPN22_Severity	Bacteria|Firmicutes|Bacilli|Lactobacillales|Streptococcaceae|Lactococcus	PTPN22_SeverityGG_Severe	0.00093778	133	47	0.28543604
PTPN22_Severity	Bacteria|Cyanobacteria|4C0d_2|YS2|	PTPN22_SeverityGG_Severe	0.00166952	133	42	0.2874697
PTPN22_Severity	Bacteria|Firmicutes|Clostridia|Clostridiales|Lachnospiraceae|Blautia	PTPN22_SeverityGG_Severe	-0.0083837	133	133	0.29178112
PTPN22_Severity	Bacteria|Firmicutes|Bacilli|Gemellales|Gemellaceae|Unclassified	PTPN22_SeverityGG_Severe	0.00115624	133	61	0.30187279
PTPN22_Severity	Bacteria|Bacteroidetes|Bacteroidia|Bacteroidales|Rikenellaceae|	PTPN22_SeverityGG_Severe	0.00921582	133	131	0.31800888
PTPN22_Severity	Bacteria|Tenericutes|Mollicutes|RF39|	PTPN22_SeverityGG_Severe	0.00191161	133	66	0.32802395
PTPN22_Severity	Bacteria|Proteobacteria|Gammaproteobacteria|Pasteurellales|Pasteurellaceae|Actinobacillus	PTPN22_SeverityGG_Severe	0.00233732	133	77	0.33640082
PTPN22_Severity	Bacteria|Firmicutes|Clostridia|Clostridiales|Peptostreptococcaceae|	PTPN22_SeverityGG_Severe	0.00121588	133	71	0.34521092
PTPN22_Severity	Bacteria|Firmicutes|Clostridia|Clostridiales|Clostridiaceae|Unclassified	PTPN22_SeverityGG_Severe	-0.0017877	133	94	0.36738584
PTPN22_Severity	Bacteria|Proteobacteria|Gammaproteobacteria|Pseudomonadales|Pseudomonadaceae|Pseudomonas	PTPN22_SeverityGG_Severe	0.00094908	133	66	0.37229664
PTPN22_Severity	Bacteria|Firmicutes|Clostridia|Clostridiales|Lachnospiraceae|	PTPN22_SeverityGG_Severe	-0.0159997	133	133	0.38290499
PTPN22_Severity	Bacteria|Firmicutes|Clostridia|Clostridiales|Ruminococcaceae|Ruminococcus	PTPN22_SeverityGG_Severe	0.00679344	133	130	0.38837228
PTPN22_Severity	Bacteria|Firmicutes|Clostridia|Clostridiales|Lachnospiraceae|[Ruminococcus_	PTPN22_SeverityGG_Severe	0.0038388	133	133	0.39597422
PTPN22_Severity	Bacteria|Firmicutes|Clostridia|Clostridiales|Lachnospiraceae|Coprococcus	PTPN22_SeverityGG_Severe	-0.0036371	133	132	0.40017441
PTPN22_Severity	Bacteria|Firmicutes|Clostridia|Clostridiales|[Mogibacteriaceae]|	PTPN22_SeverityGG_Severe	-0.0010595	133	79	0.40908117
PTPN22_Severity	Bacteria|Firmicutes|Bacilli|Lactobacillales|Lactobacillaceae|Lactobacillus	PTPN22_SeverityGG_Severe	0.00271308	133	118	0.41805147
PTPN22_Severity	Bacteria|Firmicutes|Clostridia|Clostridiales|Lachnospiraceae|Oribacterium	PTPN22_SeverityGG_Severe	0.00073312	133	40	0.42392731
PTPN22_Severity	Bacteria|Firmicutes|Clostridia|Clostridiales|Clostridiaceae|Clostridium	PTPN22_SeverityGG_Severe	0.00171934	133	96	0.43886604
PTPN22_Severity	Bacteria|Firmicutes|Clostridia|Clostridiales|Ruminococcaceae|	PTPN22_SeverityGG_Severe	-0.0127937	133	133	0.46152161
PTPN22_Severity	Bacteria|Bacteroidetes|Bacteroidia|Bacteroidales|Porphyromonadaceae|Parabacteroides	PTPN22_SeverityGG_Severe	0.00823651	133	133	0.47640249
PTPN22_Severity	Bacteria|Proteobacteria|Betaproteobacteria|Burkholderiales|Alcaligenaceae|Sutterella	PTPN22_SeverityGG_Severe	-0.0107018	133	132	0.47922258
PTPN22_Severity	Bacteria|Proteobacteria|Gammaproteobacteria|Pasteurellales|Pasteurellaceae|Haemophilus	PTPN22_SeverityGG_Severe	-0.003494	133	120	0.5064621
PTPN22_Severity	Bacteria|Actinobacteria|Actinobacteria|Actinomycetales|Propionibacteriaceae|Propionibacterium	PTPN22_SeverityGG_Severe	-0.0015373	133	81	0.50824969
PTPN22_Severity	Bacteria|Bacteroidetes|Bacteroidia|Bacteroidales|Bacteroidaceae|Unclassified	PTPN22_SeverityGG_Severe	-0.0007998	133	64	0.51624684
PTPN22_Severity	Bacteria|Firmicutes|Erysipelotrichi|Erysipelotrichales|Erysipelotrichaceae|[Eubacterium_	PTPN22_SeverityGG_Severe	0.00397009	133	122	0.52029839
PTPN22_Severity	Bacteria|Proteobacteria|Alphaproteobacteria|RF32|	PTPN22_SeverityGG_Severe	-0.0029272	133	83	0.53335802
PTPN22_Severity	Bacteria|Firmicutes|Clostridia|Clostridiales|Veillonellaceae|Dialister	PTPN22_SeverityGG_Severe	-0.0022481	133	107	0.54284025
PTPN22_Severity	Bacteria|Firmicutes|Clostridia|Clostridiales|Lachnospiraceae|Lachnospira	PTPN22_SeverityGG_Severe	0.0025197	133	128	0.54385936
PTPN22_Severity	Bacteria|Bacteroidetes|Bacteroidia|Bacteroidales|[Paraprevotellaceae]|Paraprevotella	PTPN22_SeverityGG_Severe	-0.0027726	133	61	0.55215834
PTPN22_Severity	Bacteria|Firmicutes|Clostridia|Clostridiales|Veillonellaceae|Acidaminococcus	PTPN22_SeverityGG_Severe	0.00029887	133	42	0.59000536
PTPN22_Severity	Bacteria|Cyanobacteria|Chloroplast|Streptophyta|	PTPN22_SeverityGG_Severe	0.00056693	133	45	0.59287597
PTPN22_Severity	Bacteria|Firmicutes|Erysipelotrichi|Erysipelotrichales|Erysipelotrichaceae|Unclassified	PTPN22_SeverityGG_Severe	-0.0007476	133	67	0.60454561
PTPN22_Severity	Bacteria|Firmicutes|Erysipelotrichi|Erysipelotrichales|Erysipelotrichaceae|	PTPN22_SeverityGG_Severe	0.00246691	133	129	0.61949556
PTPN22_Severity	Bacteria|Fusobacteria|Fusobacteriia|Fusobacteriales|Leptotrichiaceae|Leptotrichia	PTPN22_SeverityGG_Severe	0.00029877	133	44	0.64694595
PTPN22_Severity	Bacteria|Proteobacteria|Gammaproteobacteria|Pasteurellales|Pasteurellaceae|Aggregatibacter	PTPN22_SeverityGG_Severe	0.00056468	133	51	0.67346419
PTPN22_Severity	Bacteria|Actinobacteria|Coriobacteriia|Coriobacteriales|Coriobacteriaceae|Unclassified	PTPN22_SeverityGG_Severe	0.00029167	133	41	0.68013525
PTPN22_Severity	Bacteria|Actinobacteria|Actinobacteria|Actinomycetales|Corynebacteriaceae|Corynebacterium	PTPN22_SeverityGG_Severe	0.00051727	133	73	0.68320382
PTPN22_Severity	Bacteria|Proteobacteria|Deltaproteobacteria|Desulfovibrionales|Desulfovibrionaceae|Bilophila	PTPN22_SeverityGG_Severe	0.00165343	133	88	0.69748093
PTPN22_Severity	Bacteria|Firmicutes|Bacilli|Lactobacillales|Streptococcaceae|Unclassified	PTPN22_SeverityGG_Severe	-0.0003141	133	42	0.69881861
PTPN22_Severity	Bacteria|Firmicutes|Clostridia|Clostridiales|Lachnospiraceae|Anaerostipes	PTPN22_SeverityGG_Severe	0.00059381	133	63	0.70641011
PTPN22_Severity	Bacteria|Firmicutes|Clostridia|Clostridiales|Ruminococcaceae|Unclassified	PTPN22_SeverityGG_Severe	0.00204512	133	130	0.76111699
PTPN22_Severity	Bacteria|Firmicutes|Clostridia|Clostridiales|Veillonellaceae|Phascolarctobacterium	PTPN22_SeverityGG_Severe	0.00296764	133	99	0.76237202
PTPN22_Severity	Bacteria|Bacteroidetes|Bacteroidia|Bacteroidales|Unclassified|Unclassified	PTPN22_SeverityGG_Severe	-0.0005992	133	109	0.7657654
PTPN22_Severity	Bacteria|Bacteroidetes|Bacteroidia|Bacteroidales|[Barnesiellaceae]|	PTPN22_SeverityGG_Severe	-0.003204	133	128	0.76841741
PTPN22_Severity	Bacteria|Bacteroidetes|Bacteroidia|Bacteroidales|[Odoribacteraceae]|Odoribacter	PTPN22_SeverityGG_Severe	0.00152167	133	118	0.79306506
PTPN22_Severity	Bacteria|Actinobacteria|Coriobacteriia|Coriobacteriales|Coriobacteriaceae|Adlercreutzia	PTPN22_SeverityGG_Severe	0.00023944	133	44	0.80927606
PTPN22_Severity	Bacteria|Actinobacteria|Actinobacteria|Actinomycetales|Actinomycetaceae|Actinomyces	PTPN22_SeverityGG_Severe	0.00027956	133	82	0.82089066
PTPN22_Severity	Bacteria|Firmicutes|Clostridia|Clostridiales|Veillonellaceae|Veillonella	PTPN22_SeverityGG_Severe	0.00053083	133	105	0.83892617
PTPN22_Severity	Bacteria|Firmicutes|Clostridia|Clostridiales|Unclassified|Unclassified	PTPN22_SeverityGG_Severe	0.00119639	133	133	0.84267934
PTPN22_Severity	Bacteria|Firmicutes|Clostridia|Clostridiales|Christensenellaceae|	PTPN22_SeverityGG_Severe	-0.0008108	133	79	0.84294992
PTPN22_Severity	Bacteria|Bacteroidetes|Bacteroidia|Bacteroidales|Prevotellaceae|Prevotella	PTPN22_SeverityGG_Severe	0.00645736	133	132	0.84426377
PTPN22_Severity	Bacteria|Firmicutes|Bacilli|Bacillales|Staphylococcaceae|Staphylococcus	PTPN22_SeverityGG_Severe	0.00022383	133	68	0.85676259
PTPN22_Severity	Bacteria|Proteobacteria|Betaproteobacteria|Burkholderiales|Comamonadaceae|Tepidimonas	PTPN22_SeverityGG_Severe	-0.0001811	133	87	0.90943072
PTPN22_Severity	Bacteria|Bacteroidetes|Bacteroidia|Bacteroidales|	PTPN22_SeverityGG_Severe	-0.0001483	133	69	0.9179803
PTPN22_Severity	Bacteria|Firmicutes|Clostridia|Clostridiales|Lachnospiraceae|Unclassified	PTPN22_SeverityGG_Severe	0.00100887	133	133	0.92824122
PTPN22_Severity	Bacteria|Actinobacteria|Coriobacteriia|Coriobacteriales|Coriobacteriaceae|	PTPN22_SeverityGG_Severe	0.00015183	133	99	0.93800439
PTPN22_Severity	Bacteria|Firmicutes|Clostridia|Clostridiales|Peptostreptococcaceae|Peptostreptococcus	PTPN22_SeverityGG_Severe	9.29E-05	133	43	0.94176741
PTPN22_Severity	Bacteria|Firmicutes|Clostridia|Clostridiales|Ruminococcaceae|Oscillospira	PTPN22_SeverityGG_Severe	-0.0004286	133	132	0.94218114
PTPN22_Severity	Bacteria|Tenericutes|Mollicutes|Anaeroplasmatales|Anaeroplasmataceae|Unclassified	PTPN22_SeverityGG_Severe	-0.0007525	133	48	0.95586957
PTPN22_Severity	Bacteria|Firmicutes|Bacilli|Turicibacterales|Turicibacteraceae|Turicibacter	PTPN22_SeverityGG_Severe	6.84E-05	133	64	0.95760353
PTPN22_Severity	Bacteria|Bacteroidetes|Bacteroidia|Bacteroidales|Bacteroidaceae|Bacteroides	PTPN22_SeverityGG_Severe	0.00202741	133	133	0.96377058
PTPN22_Severity	Bacteria|Firmicutes|Clostridia|Clostridiales|	PTPN22_SeverityGG_Severe	-0.0004331	133	133	0.96653542
PTPN22_Severity	Bacteria|Firmicutes|Clostridia|Clostridiales|Lachnospiraceae|Epulopiscium	PTPN22_SeverityGG_Severe	-6.47E-07	133	68	0.99951403
