# Supplementary material for: The presence of genetic risk variants within PTPN2 and PTPN22 is associated with intestinal microbiota alterations in Swiss IBD cohort patients
Source: PLoS One. 2018 Jul 2;13(7):e0199664. doi: 10.1371/journal.pone.0199664 (PMC6028086; doi:10.1371/journal.pone.0199664)
Supplement: S5 Table — Taxonomic difference of PTPN2 variants in UC disease group was identified based on the disease severity status and significant and non-significant differences were recorded based on MaAsLin output file. Table shows coefficient value for each taxa and number of samples that were analyzed. A p-value <0.05 is considered significant. (PDF) [file pone.0199664.s006.pdf]

Suppl. Table 5. Comparison of relative abundances of different disease severities and PTPN2 variants in UC samples using MaAsLin

<table>
<tr><th>Variable</th><th>Feature</th><th>Value</th><th>Coefficient</th><th>N</th><th>N not 0</th><th>P-value</th><th>Q-value</th></tr>
<tr><td>PTPN2_Severity</td><td>Bacteria|Firmicutes|Clostridia|Clostridiales|Lachnospiraceae|Lachnobacterium</td><td>PTPN2_SeverityTT_Severe</td><td>-0.0086065</td><td>107</td><td>58</td><td>0.00035318</td><td>0.03355224</td></tr>
<tr><td>PTPN2_Severity</td><td>Bacteria|Proteobacteria|Gammaproteobacteria|Pseudomonadales|Pseudomonadaceae|Pseudomonas</td><td>PTPN2_SeverityTT_Severe</td><td>0.00326238</td><td>107</td><td>48</td><td>0.00179475</td><td>0.08525071</td></tr>
<tr><td>PTPN2_Severity</td><td>Bacteria|Firmicutes|Clostridia|Clostridiales|Ruminococcaceae|Faecalibacterium</td><td>PTPN2_SeverityTT_Severe</td><td>-0.0746171</td><td>107</td><td>107</td><td>0.0032528</td><td>0.08996042</td></tr>
<tr><td>PTPN2_Severity</td><td>Bacteria|Firmicutes|Clostridia|Clostridiales|Lachnospiraceae|Dorea</td><td>PTPN2_SeverityTT_Severe</td><td>0.00756711</td><td>107</td><td>104</td><td>0.00384079</td><td>0.08996042</td></tr>
<tr><td>PTPN2_Severity</td><td>Bacteria|Proteobacteria|Gammaproteobacteria|Enterobacteriales|Enterobacteriaceae|Klebsiella</td><td>PTPN2_SeverityTT_Severe</td><td>0.00254054</td><td>107</td><td>37</td><td>0.00531193</td><td>0.08996042</td></tr>
<tr><td>PTPN2_Severity</td><td>Bacteria|Proteobacteria|Gammaproteobacteria|Enterobacteriales|Enterobacteriaceae|Unclassified</td><td>PTPN2_SeverityTT_Severe</td><td>0.00565534</td><td>107</td><td>58</td><td>0.00568171</td><td>0.08996042</td></tr>
<tr><td>PTPN2_Severity</td><td>Bacteria|Proteobacteria|Gammaproteobacteria|Enterobacteriales|Enterobacteriaceae|</td><td>PTPN2_SeverityTT_Severe</td><td>0.0467068</td><td>107</td><td>102</td><td>0.00722914</td><td>0.09568366</td></tr>
<tr><td>PTPN2_Severity</td><td>Bacteria|Firmicutes|Clostridia|Clostridiales|Ruminococcaceae|Unclassified</td><td>PTPN2_SeverityTT_Severe</td><td>-0.016607</td><td>107</td><td>105</td><td>0.00805757</td><td>0.09568366</td></tr>
<tr><td>PTPN2_Severity</td><td>Bacteria|Firmicutes|Bacilli|Lactobacillales|Lactobacillaceae|Lactobacillus</td><td>PTPN2_SeverityTT_Severe</td><td>0.00820504</td><td>107</td><td>91</td><td>0.0198955</td><td>0.19850322</td></tr>
<tr><td>PTPN2_Severity</td><td>Bacteria|Actinobacteria|Actinobacteria|Bifidobacteriales|Bifidobacteriaceae|Bifidobacterium</td><td>PTPN2_SeverityTT_Severe</td><td>0.01324271</td><td>107</td><td>93</td><td>0.02089508</td><td>0.19850322</td></tr>
<tr><td>PTPN2_Severity</td><td>Bacteria|Firmicutes|Clostridia|Clostridiales|Ruminococcaceae|</td><td>PTPN2_SeverityTT_Severe</td><td>-0.0423184</td><td>107</td><td>107</td><td>0.02419383</td><td>0.20894673</td></tr>
<tr><td>PTPN2_Severity</td><td>Bacteria|Firmicutes|Clostridia|Clostridiales|Lachnospiraceae|Coprococcus</td><td>PTPN2_SeverityTT_Severe</td><td>-0.0099318</td><td>107</td><td>107</td><td>0.03564207</td><td>0.2702808</td></tr>
<tr><td>PTPN2_Severity</td><td>Bacteria|Bacteroidetes|Bacteroidia|Bacteroidales|[Odoribacteraceae]|Butyricimonas</td><td>PTPN2_SeverityTT_Severe</td><td>-0.0073957</td><td>107</td><td>66</td><td>0.03698579</td><td>0.2702808</td></tr>
<tr><td>PTPN2_Severity</td><td>Bacteria|Firmicutes|Erysipelotrichi|Erysipelotrichales|Erysipelotrichaceae|Coprobacillus</td><td>PTPN2_SeverityTT_Severe</td><td>-0.0028552</td><td>107</td><td>49</td><td>0.04234116</td><td>0.28415034</td></tr>
<tr><td>PTPN2_Severity</td><td>Bacteria|Actinobacteria|Coriobacteriia|Coriobacteriales|Coriobacteriaceae|Eggerthella</td><td>PTPN2_SeverityTT_Severe</td><td>-0.0028771</td><td>107</td><td>51</td><td>0.04486584</td><td>0.28415034</td></tr>
<tr><td>PTPN2_Severity</td><td>Bacteria|Firmicutes|Bacilli|Lactobacillales|Streptococcaceae|Streptococcus</td><td>PTPN2_SeverityTT_Severe</td><td>0.00670561</td><td>107</td><td>105</td><td>0.05268458</td><td>0.31281468</td></tr>
<tr><td>PTPN2_Severity</td><td>Bacteria|Actinobacteria|Actinobacteria|Actinomycetales|Corynebacteriaceae|Corynebacterium</td><td>PTPN2_SeverityTT_Severe</td><td>0.00240802</td><td>107</td><td>58</td><td>0.06427968</td><td>0.3577365</td></tr>
<tr><td>PTPN2_Severity</td><td>Bacteria|Bacteroidetes|Bacteroidia|Bacteroidales|[Barnesiellaceae]|</td><td>PTPN2_SeverityTT_Severe</td><td>-0.0206695</td><td>107</td><td>102</td><td>0.07394407</td><td>0.3577365</td></tr>
<tr><td>PTPN2_Severity</td><td>Bacteria|Bacteroidetes|Bacteroidia|Bacteroidales|Unclassified|Unclassified</td><td>PTPN2_SeverityTT_Severe</td><td>-0.0032786</td><td>107</td><td>88</td><td>0.07960756</td><td>0.3577365</td></tr>
<tr><td>PTPN2_Severity</td><td>Bacteria|Proteobacteria|Betaproteobacteria|Burkholderiales|Comamonadaceae|Tepidimonas</td><td>PTPN2_SeverityTT_Severe</td><td>0.00280642</td><td>107</td><td>70</td><td>0.08102844</td><td>0.3577365</td></tr>
<tr><td>PTPN2_Severity</td><td>Bacteria|Proteobacteria|Gammaproteobacteria|Pseudomonadales|Moraxellaceae|Acinetobacter</td><td>PTPN2_SeverityTT_Severe</td><td>0.00200271</td><td>107</td><td>48</td><td>0.08206635</td><td>0.3577365</td></tr>
<tr><td>PTPN2_Severity</td><td>Bacteria|Firmicutes|Clostridia|Clostridiales|Peptostreptococcaceae|</td><td>PTPN2_SeverityTT_Severe</td><td>0.00229093</td><td>107</td><td>60</td><td>0.091943</td><td>0.3577365</td></tr>
<tr><td>PTPN2_Severity</td><td>Bacteria|Actinobacteria|Actinobacteria|Actinomycetales|Micrococcaceae|Rothia</td><td>PTPN2_SeverityTT_Severe</td><td>0.00222653</td><td>107</td><td>50</td><td>0.09421908</td><td>0.3577365</td></tr>
<tr><td>PTPN2_Severity</td><td>Bacteria|Cyanobacteria|Chloroplast|Streptophyta|</td><td>PTPN2_SeverityTT_Severe</td><td>0.00127244</td><td>107</td><td>35</td><td>0.09506611</td><td>0.3577365</td></tr>
<tr><td>PTPN2_Severity</td><td>Bacteria|Verrucomicrobia|Verrucomicrobiae|Verrucomicrobiales|Verrucomicrobiaceae|Akkermansia</td><td>PTPN2_SeverityTT_Severe</td><td>-0.012254</td><td>107</td><td>83</td><td>0.09791322</td><td>0.3577365</td></tr>
<tr><td>PTPN2_Severity</td><td>Bacteria|Firmicutes|Bacilli|Lactobacillales|Enterococcaceae|Enterococcus</td><td>PTPN2_SeverityTT_Severe</td><td>0.0016239</td><td>107</td><td>43</td><td>0.10192708</td><td>0.3577365</td></tr>
<tr><td>PTPN2_Severity</td><td>Bacteria|Firmicutes|Clostridia|Clostridiales|Lachnospiraceae|</td><td>PTPN2_SeverityTT_Severe</td><td>-0.0293127</td><td>107</td><td>107</td><td>0.10218703</td><td>0.3577365</td></tr>
<tr><td>PTPN2_Severity</td><td>Bacteria|Actinobacteria|Actinobacteria|Actinomycetales|Propionibacteriaceae|Propionibacterium</td><td>PTPN2_SeverityTT_Severe</td><td>0.00325633</td><td>107</td><td>66</td><td>0.10543813</td><td>0.3577365</td></tr>
<tr><td>PTPN2_Severity</td><td>Bacteria|Firmicutes|Erysipelotrichi|Erysipelotrichales|Erysipelotrichaceae|[Eubacterium_</td><td>PTPN2_SeverityTT_Severe</td><td>0.00939465</td><td>107</td><td>97</td><td>0.10964572</td><td>0.35918426</td></tr>
<tr><td>PTPN2_Severity</td><td>Bacteria|Firmicutes|Clostridia|Clostridiales|Lachnospiraceae|Blautia</td><td>PTPN2_SeverityTT_Severe</td><td>-0.0142554</td><td>107</td><td>107</td><td>0.1162029</td><td>0.36541996</td></tr>
<tr><td>PTPN2_Severity</td><td>Bacteria|Bacteroidetes|Bacteroidia|Bacteroidales|Prevotellaceae|Prevotella</td><td>PTPN2_SeverityTT_Severe</td><td>0.0514279</td><td>107</td><td>107</td><td>0.1192423</td><td>0.36541996</td></tr>
<tr><td>PTPN2_Severity</td><td>Bacteria|Bacteroidetes|Bacteroidia|Bacteroidales|Bacteroidaceae|Unclassified</td><td>PTPN2_SeverityTT_Severe</td><td>-0.0019505</td><td>107</td><td>52</td><td>0.12817712</td><td>0.38052583</td></tr>
<tr><td>PTPN2_Severity</td><td>Bacteria|Firmicutes|Clostridia|Clostridiales|Clostridiaceae|</td><td>PTPN2_SeverityTT_Severe</td><td>-0.005656</td><td>107</td><td>91</td><td>0.14539335</td><td>0.4185566</td></tr>
<tr><td>PTPN2_Severity</td><td>Bacteria|Firmicutes|Clostridia|Clostridiales|Ruminococcaceae|Oscillospira</td><td>PTPN2_SeverityTT_Severe</td><td>-0.0089214</td><td>107</td><td>106</td><td>0.15219627</td><td>0.42525429</td></tr>
<tr><td>PTPN2_Severity</td><td>Bacteria|Firmicutes|Clostridia|Clostridiales|Peptostreptococcaceae|Peptostreptococcus</td><td>PTPN2_SeverityTT_Severe</td><td>0.00165221</td><td>107</td><td>34</td><td>0.16542604</td><td>0.44901354</td></tr>
<tr><td>PTPN2_Severity</td><td>Bacteria|Fusobacteria|Fusobacteriia|Fusobacteriales|Fusobacteriaceae|Fusobacterium</td><td>PTPN2_SeverityTT_Severe</td><td>0.00470673</td><td>107</td><td>87</td><td>0.17632445</td><td>0.459776</td></tr>
<tr><td>PTPN2_Severity</td><td>Bacteria|Actinobacteria|Coriobacteriia|Coriobacteriales|Coriobacteriaceae|Collinsella</td><td>PTPN2_SeverityTT_Severe</td><td>0.0071896</td><td>107</td><td>96</td><td>0.17907065</td><td>0.459776</td></tr>
<tr><td>PTPN2_Severity</td><td>Bacteria|Firmicutes|Bacilli|Bacillales|Unclassified|Unclassified</td><td>PTPN2_SeverityTT_Severe</td><td>0.00198376</td><td>107</td><td>69</td><td>0.1845124</td><td>0.46128101</td></tr>
<tr><td>PTPN2_Severity</td><td>Bacteria|Proteobacteria|Gammaproteobacteria|Unclassified|Unclassified|Unclassified</td><td>PTPN2_SeverityTT_Severe</td><td>0.00104079</td><td>107</td><td>35</td><td>0.21639504</td><td>0.51783222</td></tr>
<tr><td>PTPN2_Severity</td><td>Bacteria|Firmicutes|Clostridia|Clostridiales|Ruminococcaceae|Ruminococcus</td><td>PTPN2_SeverityTT_Severe</td><td>0.01016108</td><td>107</td><td>104</td><td>0.21803462</td><td>0.51783222</td></tr>
<tr><td>PTPN2_Severity</td><td>Bacteria|Firmicutes|Clostridia|Clostridiales|Lachnospiraceae|[Ruminococcus_</td><td>PTPN2_SeverityTT_Severe</td><td>0.00646877</td><td>107</td><td>107</td><td>0.22833675</td><td>0.52907295</td></tr>
<tr><td>PTPN2_Severity</td><td>Bacteria|Firmicutes|Bacilli|Lactobacillales|Streptococcaceae|Lactococcus</td><td>PTPN2_SeverityTT_Severe</td><td>0.0011044</td><td>107</td><td>42</td><td>0.2501102</td><td>0.56572546</td></tr>
<tr><td>PTPN2_Severity</td><td>Bacteria|Bacteroidetes|Bacteroidia|Bacteroidales|[Paraprevotellaceae]|[Prevotella_</td><td>PTPN2_SeverityTT_Severe</td><td>0.00215166</td><td>107</td><td>80</td><td>0.25619686</td><td>0.56601632</td></tr>
<tr><td>PTPN2_Severity</td><td>Bacteria|Bacteroidetes|Bacteroidia|Bacteroidales|S24_7|</td><td>PTPN2_SeverityTT_Severe</td><td>0.0073175</td><td>107</td><td>104</td><td>0.28039418</td><td>0.60539652</td></tr>
<tr><td>PTPN2_Severity</td><td>Bacteria|Proteobacteria|Gammaproteobacteria|Enterobacteriales|Enterobacteriaceae|Gluconacetobacter</td><td>PTPN2_SeverityTT_Severe</td><td>0.00077262</td><td>107</td><td>33</td><td>0.29322802</td><td>0.61667932</td></tr>
<tr><td>PTPN2_Severity</td><td>Bacteria|Proteobacteria|Betaproteobacteria|Neisseriales|Neisseriaceae|Neisseria</td><td>PTPN2_SeverityTT_Severe</td><td>0.00133138</td><td>107</td><td>56</td><td>0.29860262</td><td>0.61667932</td></tr>
<tr><td>PTPN2_Severity</td><td>Bacteria|Firmicutes|Clostridia|Clostridiales|Lachnospiraceae|Unclassified</td><td>PTPN2_SeverityTT_Severe</td><td>-0.0125705</td><td>107</td><td>107</td><td>0.30793256</td><td>0.62241688</td></tr>
<tr><td>PTPN2_Severity</td><td>Bacteria|Proteobacteria|Betaproteobacteria|Burkholderiales|Comamonadaceae|</td><td>PTPN2_SeverityTT_Severe</td><td>0.00109164</td><td>107</td><td>38</td><td>0.319335</td><td>0.63201718</td></tr>
<tr><td>PTPN2_Severity</td><td>Bacteria|Firmicutes|Bacilli|Bacillales|Staphylococcaceae|Staphylococcus</td><td>PTPN2_SeverityTT_Severe</td><td>0.00100356</td><td>107</td><td>50</td><td>0.33797695</td><td>0.64351415</td></tr>
<tr><td>PTPN2_Severity</td><td>Bacteria|Firmicutes|Bacilli|Gemellales|Gemellaceae|Unclassified</td><td>PTPN2_SeverityTT_Severe</td><td>0.00123416</td><td>107</td><td>46</td><td>0.34506587</td><td>0.64351415</td></tr>
<tr><td>PTPN2_Severity</td><td>Bacteria|Actinobacteria|Actinobacteria|Actinomycetales|Actinomycetaceae|Actinomyces</td><td>PTPN2_SeverityTT_Severe</td><td>0.00128664</td><td>107</td><td>65</td><td>0.34546549</td><td>0.64351415</td></tr>
<tr><td>PTPN2_Severity</td><td>Bacteria|Firmicutes|Clostridia|Clostridiales|Ruminococcaceae|Anaerotruncus</td><td>PTPN2_SeverityTT_Severe</td><td>0.00065422</td><td>107</td><td>35</td><td>0.37359919</td><td>0.68253699</td></tr>
<tr><td>PTPN2_Severity</td><td>Bacteria|Proteobacteria|Alphaproteobacteria|RF32|</td><td>PTPN2_SeverityTT_Severe</td><td>-0.0047341</td><td>107</td><td>66</td><td>0.38702201</td><td>0.6937187</td></tr>
<tr><td>PTPN2_Severity</td><td>Bacteria|Proteobacteria|Gammaproteobacteria|Pasteurellales|Pasteurellaceae|Haemophilus</td><td>PTPN2_SeverityTT_Severe</td><td>-0.0048957</td><td>107</td><td>96</td><td>0.41816726</td><td>0.72119996</td></tr>
<tr><td>PTPN2_Severity</td><td>Bacteria|Bacteroidetes|Bacteroidia|Bacteroidales|Bacteroidaceae|Bacteroides</td><td>PTPN2_SeverityTT_Severe</td><td>-0.0383563</td><td>107</td><td>107</td><td>0.41828502</td><td>0.72119996</td></tr>
<tr><td>PTPN2_Severity</td><td>Bacteria|Proteobacteria|Betaproteobacteria|Neisseriales|Neisseriaceae|</td><td>PTPN2_SeverityTT_Severe</td><td>0.00052765</td><td>107</td><td>33</td><td>0.4251284</td><td>0.72119996</td></tr>
<tr><td>PTPN2_Severity</td><td>Bacteria|Firmicutes|Clostridia|Clostridiales|Lachnospiraceae|Anaerostipes</td><td>PTPN2_SeverityTT_Severe</td><td>0.00098893</td><td>107</td><td>48</td><td>0.43633747</td><td>0.72722911</td></tr>
<tr><td>PTPN2_Severity</td><td>Bacteria|Firmicutes|Erysipelotrichi|Erysipelotrichales|Erysipelotrichaceae|Catenibacterium</td><td>PTPN2_SeverityTT_Severe</td><td>0.00032841</td><td>107</td><td>37</td><td>0.48065775</td><td>0.78728425</td></tr>
<tr><td>PTPN2_Severity</td><td>Bacteria|Firmicutes|Clostridia|Clostridiales|Lachnospiraceae|Roseburia</td><td>PTPN2_SeverityTT_Severe</td><td>-0.0068342</td><td>107</td><td>107</td><td>0.49454156</td><td>0.79629574</td></tr>
<tr><td>PTPN2_Severity</td><td>Bacteria|Bacteroidetes|Bacteroidia|Bacteroidales|Rikenellaceae|</td><td>PTPN2_SeverityTT_Severe</td><td>-0.0054876</td><td>107</td><td>106</td><td>0.53699858</td><td>0.85024776</td></tr>
<tr><td>PTPN2_Severity</td><td>Bacteria|Actinobacteria|Coriobacteriia|Coriobacteriales|Coriobacteriaceae|Adlercreutzia</td><td>PTPN2_SeverityTT_Severe</td><td>0.00065597</td><td>107</td><td>33</td><td>0.55213451</td><td>0.85988161</td></tr>
<tr><td>PTPN2_Severity</td><td>Bacteria|Firmicutes|Clostridia|Clostridiales|</td><td>PTPN2_SeverityTT_Severe</td><td>-0.0062848</td><td>107</td><td>107</td><td>0.57792981</td><td>0.88553761</td></tr>
<tr><td>PTPN2_Severity</td><td>Bacteria|Firmicutes|Clostridia|Clostridiales|Lachnospiraceae|Oribacterium</td><td>PTPN2_SeverityTT_Severe</td><td>0.00061939</td><td>107</td><td>38</td><td>0.59250016</td><td>0.89345263</td></tr>
<tr><td>PTPN2_Severity</td><td>Bacteria|Firmicutes|Clostridia|Clostridiales|Veillonellaceae|Dialister</td><td>PTPN2_SeverityTT_Severe</td><td>-0.002116</td><td>107</td><td>88</td><td>0.60375376</td><td>0.89619699</td></tr>
<tr><td>PTPN2_Severity</td><td>Bacteria|Bacteroidetes|Bacteroidia|Bacteroidales|Porphyromonadaceae|Parabacteroides</td><td>PTPN2_SeverityTT_Severe</td><td>-0.0066827</td><td>107</td><td>107</td><td>0.61683613</td><td>0.90152972</td></tr>
<tr><td>PTPN2_Severity</td><td>Bacteria|Actinobacteria|Coriobacteriia|Coriobacteriales|Coriobacteriaceae|Unclassified</td><td>PTPN2_SeverityTT_Severe</td><td>0.00034089</td><td>107</td><td>34</td><td>0.66147534</td><td>0.94176115</td></tr>
<tr><td>PTPN2_Severity</td><td>Bacteria|Fusobacteria|Fusobacteriia|Fusobacteriales|Leptotrichiaceae|Leptotrichia</td><td>PTPN2_SeverityTT_Severe</td><td>0.00031342</td><td>107</td><td>39</td><td>0.66595796</td><td>0.94176115</td></tr>
<tr><td>PTPN2_Severity</td><td>Bacteria|Firmicutes|Clostridia|Clostridiales|Lachnospiraceae|Lachnospira</td><td>PTPN2_SeverityTT_Severe</td><td>0.00187631</td><td>107</td><td>103</td><td>0.6798386</td><td>0.94176115</td></tr>
<tr><td>PTPN2_Severity</td><td>Bacteria|Proteobacteria|Deltaproteobacteria|Desulfovibrionales|Desulfovibrionaceae|Bilophila</td><td>PTPN2_SeverityTT_Severe</td><td>-0.0018693</td><td>107</td><td>77</td><td>0.69209056</td><td>0.94176115</td></tr>
<tr><td>PTPN2_Severity</td><td>Bacteria|Tenericutes|Mollicutes|RF39|</td><td>PTPN2_SeverityTT_Severe</td><td>0.00075873</td><td>107</td><td>48</td><td>0.70277644</td><td>0.94176115</td></tr>
<tr><td>PTPN2_Severity</td><td>Bacteria|Firmicutes|Clostridia|Clostridiales|Veillonellaceae|Phascolarctobacterium</td><td>PTPN2_SeverityTT_Severe</td><td>-0.0041304</td><td>107</td><td>77</td><td>0.70384254</td><td>0.94176115</td></tr>
<tr><td>PTPN2_Severity</td><td>Bacteria|Firmicutes|Clostridia|Clostridiales|Veillonellaceae|Megasphaera</td><td>PTPN2_SeverityTT_Severe</td><td>-0.0003821</td><td>107</td><td>39</td><td>0.7325048</td><td>0.94628824</td></tr>
<tr><td>PTPN2_Severity</td><td>Bacteria|Bacteroidetes|Bacteroidia|Bacteroidales|</td><td>PTPN2_SeverityTT_Severe</td><td>-0.0004846</td><td>107</td><td>56</td><td>0.73327737</td><td>0.94628824</td></tr>
<tr><td>PTPN2_Severity</td><td>Bacteria|Bacteroidetes|Bacteroidia|Bacteroidales|[Odoribacteraceae]|Odoribacter</td><td>PTPN2_SeverityTT_Severe</td><td>-0.0019479</td><td>107</td><td>94</td><td>0.73894465</td><td>0.94628824</td></tr>
<tr><td>PTPN2_Severity</td><td>Bacteria|Firmicutes|Clostridia|Clostridiales|Veillonellaceae|Veillonella</td><td>PTPN2_SeverityTT_Severe</td><td>0.00089192</td><td>107</td><td>86</td><td>0.75458235</td><td>0.94628824</td></tr>
<tr><td>PTPN2_Severity</td><td>Bacteria|Cyanobacteria|4C0d_2|YS2|</td><td>PTPN2_SeverityTT_Severe</td><td>0.00060765</td><td>107</td><td>38</td><td>0.76276451</td><td>0.94628824</td></tr>
<tr><td>PTPN2_Severity</td><td>Bacteria|Firmicutes|Clostridia|Clostridiales|Veillonellaceae|Unclassified</td><td>PTPN2_SeverityTT_Severe</td><td>-0.0003729</td><td>107</td><td>51</td><td>0.76699152</td><td>0.94628824</td></tr>
<tr><td>PTPN2_Severity</td><td>Bacteria|Tenericutes|Mollicutes|Anaeroplasmatales|Anaeroplasmataceae|Unclassified</td><td>PTPN2_SeverityTT_Severe</td><td>0.00400416</td><td>107</td><td>40</td><td>0.79350853</td><td>0.96571988</td></tr>
<tr><td>PTPN2_Severity</td><td>Bacteria|Bacteroidetes|Bacteroidia|Bacteroidales|[Paraprevotellaceae]|Paraprevotella</td><td>PTPN2_SeverityTT_Severe</td><td>0.00074476</td><td>107</td><td>49</td><td>0.80408352</td><td>0.96571988</td></tr>
<tr><td>PTPN2_Severity</td><td>Bacteria|Bacteroidetes|Bacteroidia|Bacteroidales|Porphyromonadaceae|Porphyromonas</td><td>PTPN2_SeverityTT_Severe</td><td>0.0002667</td><td>107</td><td>43</td><td>0.81323779</td><td>0.96571988</td></tr>
<tr><td>PTPN2_Severity</td><td>Bacteria|Firmicutes|Bacilli|Turicibacterales|Turicibacteraceae|Turicibacter</td><td>PTPN2_SeverityTT_Severe</td><td>0.00026162</td><td>107</td><td>49</td><td>0.85496361</td><td>0.96888798</td></tr>
<tr><td>PTPN2_Severity</td><td>Bacteria|Firmicutes|Clostridia|Clostridiales|Lachnospiraceae|Epulopiscium</td><td>PTPN2_SeverityTT_Severe</td><td>0.00017692</td><td>107</td><td>54</td><td>0.87615197</td><td>0.96888798</td></tr>
<tr><td>PTPN2_Severity</td><td>Bacteria|Firmicutes|Erysipelotrichi|Erysipelotrichales|Erysipelotrichaceae|</td><td>PTPN2_SeverityTT_Severe</td><td>0.00069717</td><td>107</td><td>104</td><td>0.87700158</td><td>0.96888798</td></tr>
<tr><td>PTPN2_Severity</td><td>Bacteria|Proteobacteria|Betaproteobacteria|Burkholderiales|Alcaligenaceae|Sutterella</td><td>PTPN2_SeverityTT_Severe</td><td>-0.0026051</td><td>107</td><td>107</td><td>0.87988245</td><td>0.96888798</td></tr>
<tr><td>PTPN2_Severity</td><td>Bacteria|Firmicutes|Clostridia|Clostridiales|Unclassified|Unclassified</td><td>PTPN2_SeverityTT_Severe</td><td>-0.0008486</td><td>107</td><td>107</td><td>0.89235186</td><td>0.96888798</td></tr>
<tr><td>PTPN2_Severity</td><td>Bacteria|Proteobacteria|Gammaproteobacteria|Pasteurellales|Pasteurellaceae|Actinobacillus</td><td>PTPN2_SeverityTT_Severe</td><td>-0.0003882</td><td>107</td><td>65</td><td>0.89412663</td><td>0.96888798</td></tr>
<tr><td>PTPN2_Severity</td><td>Bacteria|Actinobacteria|Coriobacteriia|Coriobacteriales|Coriobacteriaceae|</td><td>PTPN2_SeverityTT_Severe</td><td>-0.0002583</td><td>107</td><td>80</td><td>0.90228089</td><td>0.96888798</td></tr>
<tr><td>PTPN2_Severity</td><td>Bacteria|Firmicutes|Clostridia|Clostridiales|Clostridiaceae|Unclassified</td><td>PTPN2_SeverityTT_Severe</td><td>-0.0001946</td><td>107</td><td>73</td><td>0.91065441</td><td>0.96888798</td></tr>
<tr><td>PTPN2_Severity</td><td>Bacteria|Firmicutes|Erysipelotrichi|Erysipelotrichales|Erysipelotrichaceae|Unclassified</td><td>PTPN2_SeverityTT_Severe</td><td>0.00016288</td><td>107</td><td>52</td><td>0.91913923</td><td>0.96888798</td></tr>
<tr><td>PTPN2_Severity</td><td>Bacteria|Firmicutes|Clostridia|Clostridiales|Clostridiaceae|Clostridium</td><td>PTPN2_SeverityTT_Severe</td><td>0.00018496</td><td>107</td><td>79</td><td>0.91941361</td><td>0.96888798</td></tr>
<tr><td>PTPN2_Severity</td><td>Bacteria|Firmicutes|Clostridia|Clostridiales|Christensenellaceae|</td><td>PTPN2_SeverityTT_Severe</td><td>0.00036786</td><td>107</td><td>60</td><td>0.93321062</td><td>0.96888798</td></tr>
<tr><td>PTPN2_Severity</td><td>Bacteria|Firmicutes|Clostridia|Clostridiales|[Mogibacteriaceae]|</td><td>PTPN2_SeverityTT_Severe</td><td>-0.0001045</td><td>107</td><td>66</td><td>0.93829152</td><td>0.96888798</td></tr>
<tr><td>PTPN2_Severity</td><td>Bacteria|Proteobacteria|Deltaproteobacteria|Desulfovibrionales|Desulfovibrionaceae|Desulfovibrio</td><td>PTPN2_SeverityTT_Severe</td><td>-5.18E-05</td><td>107</td><td>50</td><td>0.96012428</td><td>0.98077212</td></tr>
<tr><td>PTPN2_Severity</td><td>Bacteria|Firmicutes|Clostridia|Clostridiales|Veillonellaceae|</td><td>PTPN2_SeverityTT_Severe</td><td>2.34E-05</td><td>107</td><td>42</td><td>0.98286929</td><td>0.98955758</td></tr>
<tr><td>PTPN2_Severity</td><td>Bacteria|Proteobacteria|Gammaproteobacteria|Pasteurellales|Pasteurellaceae|Aggregatibacter</td><td>PTPN2_SeverityTT_Severe</td><td>-2.45E-05</td><td>107</td><td>44</td><td>0.98955758</td><td>0.98955758</td></tr>
<tr><td>PTPN2_Severity</td><td>Bacteria|Firmicutes|Clostridia|Clostridiales|Ruminococcaceae|Unclassified</td><td>PTPN2_SeverityCT_Severe</td><td>0.06603122</td><td>41</td><td>40</td><td>3.58E-07</td><td>3.15E-05</td></tr>
<tr><td>PTPN2_Severity</td><td>Bacteria|Firmicutes|Clostridia|Clostridiales|Ruminococcaceae|Oscillospira</td><td>PTPN2_SeverityCT_Severe</td><td>0.03797621</td><td>41</td><td>41</td><td>0.00321615</td><td>0.10700327</td></tr>
<tr><td>PTPN2_Severity</td><td>Bacteria|Firmicutes|Clostridia|Clostridiales|Ruminococcaceae|</td><td>PTPN2_SeverityCT_Severe</td><td>0.10106289</td><td>41</td><td>41</td><td>0.00439844</td><td>0.10700327</td></tr>
<tr><td>PTPN2_Severity</td><td>Bacteria|Bacteroidetes|Bacteroidia|Bacteroidales|[Paraprevotellaceae]|[Prevotella_</td><td>PTPN2_SeverityCT_Severe</td><td>0.00605976</td><td>41</td><td>31</td><td>0.00670254</td><td>0.10700327</td></tr>
<tr><td>PTPN2_Severity</td><td>Bacteria|Proteobacteria|Betaproteobacteria|Burkholderiales|Comamonadaceae|Tepidimonas</td><td>PTPN2_SeverityCT_Severe</td><td>-0.0080812</td><td>41</td><td>27</td><td>0.00777096</td><td>0.10700327</td></tr>
<tr><td>PTPN2_Severity</td><td>Bacteria|Tenericutes|Mollicutes|RF39|</td><td>PTPN2_SeverityCT_Severe</td><td>0.01106452</td><td>41</td><td>21</td><td>0.00866678</td><td>0.10700327</td></tr>
<tr><td>PTPN2_Severity</td><td>Bacteria|Firmicutes|Clostridia|Clostridiales|Veillonellaceae|Unclassified</td><td>PTPN2_SeverityCT_Severe</td><td>0.0089677</td><td>41</td><td>19</td><td>0.0096247</td><td>0.10700327</td></tr>
<tr><td>PTPN2_Severity</td><td>Bacteria|Firmicutes|Clostridia|Clostridiales|Lachnospiraceae|Unclassified</td><td>PTPN2_SeverityCT_Severe</td><td>0.04850464</td><td>41</td><td>41</td><td>0.01105995</td><td>0.10700327</td></tr>
<tr><td>PTPN2_Severity</td><td>Bacteria|Proteobacteria|Betaproteobacteria|Burkholderiales|Comamonadaceae|</td><td>PTPN2_SeverityCT_Severe</td><td>-0.006201</td><td>41</td><td>17</td><td>0.0117077</td><td>0.10700327</td></tr>
<tr><td>PTPN2_Severity</td><td>Bacteria|Bacteroidetes|Bacteroidia|Bacteroidales|S24_7|</td><td>PTPN2_SeverityCT_Severe</td><td>-0.0423296</td><td>41</td><td>40</td><td>0.01215946</td><td>0.10700327</td></tr>
<tr><td>PTPN2_Severity</td><td>Bacteria|Proteobacteria|Gammaproteobacteria|Pasteurellales|Pasteurellaceae|Aggregatibacter</td><td>PTPN2_SeverityCT_Severe</td><td>0.0040674</td><td>41</td><td>13</td><td>0.01435772</td><td>0.10741986</td></tr>
<tr><td>PTPN2_Severity</td><td>Bacteria|Firmicutes|Clostridia|Clostridiales|Lachnospiraceae|Blautia</td><td>PTPN2_SeverityCT_Severe</td><td>0.03769273</td><td>41</td><td>41</td><td>0.01464816</td><td>0.10741986</td></tr>
<tr><td>PTPN2_Severity</td><td>Bacteria|Proteobacteria|Gammaproteobacteria|Xanthomonadales|Xanthomonadaceae|</td><td>PTPN2_SeverityCT_Severe</td><td>-0.0040989</td><td>41</td><td>13</td><td>0.01977547</td><td>0.12939906</td></tr>
<tr><td>PTPN2_Severity</td><td>Bacteria|Firmicutes|Clostridia|Clostridiales|Lachnospiraceae|Coprococcus</td><td>PTPN2_SeverityCT_Severe</td><td>0.01877155</td><td>41</td><td>40</td><td>0.02058621</td><td>0.12939906</td></tr>
<tr><td>PTPN2_Severity</td><td>Bacteria|Bacteroidetes|Bacteroidia|Bacteroidales|Rikenellaceae|</td><td>PTPN2_SeverityCT_Severe</td><td>0.04806325</td><td>41</td><td>40</td><td>0.02390393</td><td>0.14023637</td></tr>
<tr><td>PTPN2_Severity</td><td>Bacteria|Bacteroidetes|Bacteroidia|Bacteroidales|Bacteroidaceae|Unclassified</td><td>PTPN2_SeverityCT_Severe</td><td>0.00835005</td><td>41</td><td>21</td><td>0.02902139</td><td>0.15961766</td></tr>
<tr><td>PTPN2_Severity</td><td>Bacteria|Proteobacteria|Deltaproteobacteria|Desulfovibrionales|Desulfovibrionaceae|Desulfovibrio</td><td>PTPN2_SeverityCT_Severe</td><td>-0.0051752</td><td>41</td><td>22</td><td>0.03954517</td><td>0.20463293</td></tr>
<tr><td>PTPN2_Severity</td><td>Bacteria|Bacteroidetes|Bacteroidia|Bacteroidales|[Barnesiellaceae]|</td><td>PTPN2_SeverityCT_Severe</td><td>0.04664297</td><td>41</td><td>41</td><td>0.04185674</td><td>0.20463293</td></tr>
<tr><td>PTPN2_Severity</td><td>Bacteria|Firmicutes|Clostridia|Clostridiales|Lachnospiraceae|Lachnobacterium</td><td>PTPN2_SeverityCT_Severe</td><td>-0.0201585</td><td>41</td><td>30</td><td>0.04705348</td><td>0.21793193</td></tr>
<tr><td>PTPN2_Severity</td><td>Bacteria|Bacteroidetes|Bacteroidia|Bacteroidales|Unclassified|Unclassified</td><td>PTPN2_SeverityCT_Severe</td><td>0.00919842</td><td>41</td><td>35</td><td>0.0586194</td><td>0.23216577</td></tr>
<tr><td>PTPN2_Severity</td><td>Bacteria|Firmicutes|Clostridia|Clostridiales|Clostridiaceae|Clostridium</td><td>PTPN2_SeverityCT_Severe</td><td>0.00837698</td><td>41</td><td>31</td><td>0.05904447</td><td>0.23216577</td></tr>
<tr><td>PTPN2_Severity</td><td>Bacteria|Actinobacteria|Actinobacteria|Bifidobacteriales|Bifidobacteriaceae|Bifidobacterium</td><td>PTPN2_SeverityCT_Severe</td><td>0.02054417</td><td>41</td><td>32</td><td>0.05969656</td><td>0.23216577</td></tr>
<tr><td>PTPN2_Severity</td><td>Bacteria|Bacteroidetes|Bacteroidia|Bacteroidales|Bacteroidaceae|Bacteroides</td><td>PTPN2_SeverityCT_Severe</td><td>0.1664446</td><td>41</td><td>41</td><td>0.06085613</td><td>0.23216577</td></tr>
<tr><td>PTPN2_Severity</td><td>Bacteria|Proteobacteria|Betaproteobacteria|Neisseriales|Neisseriaceae|Neisseria</td><td>PTPN2_SeverityCT_Severe</td><td>0.00338568</td><td>41</td><td>17</td><td>0.06331794</td><td>0.23216577</td></tr>
<tr><td>PTPN2_Severity</td><td>Bacteria|Firmicutes|Bacilli|Lactobacillales|Lactobacillaceae|Lactobacillus</td><td>PTPN2_SeverityCT_Severe</td><td>-0.0112212</td><td>41</td><td>40</td><td>0.08436173</td><td>0.29695331</td></tr>
<tr><td>PTPN2_Severity</td><td>Bacteria|Actinobacteria|Actinobacteria|Actinomycetales|Corynebacteriaceae|Corynebacterium</td><td>PTPN2_SeverityCT_Severe</td><td>-0.004275</td><td>41</td><td>26</td><td>0.08889594</td><td>0.30087857</td></tr>
<tr><td>PTPN2_Severity</td><td>Bacteria|Actinobacteria|Actinobacteria|Actinomycetales|Propionibacteriaceae|Propionibacterium</td><td>PTPN2_SeverityCT_Severe</td><td>-0.0092346</td><td>41</td><td>22</td><td>0.1066385</td><td>0.33912261</td></tr>
<tr><td>PTPN2_Severity</td><td>Bacteria|Firmicutes|Clostridia|Clostridiales|Lachnospiraceae|Roseburia</td><td>PTPN2_SeverityCT_Severe</td><td>0.01792663</td><td>41</td><td>41</td><td>0.10790265</td><td>0.33912261</td></tr>
<tr><td>PTPN2_Severity</td><td>Bacteria|Fusobacteria|Fusobacteriia|Fusobacteriales|Fusobacteriaceae|Fusobacterium</td><td>PTPN2_SeverityCT_Severe</td><td>0.00745539</td><td>41</td><td>39</td><td>0.11211182</td><td>0.34020139</td></tr>
<tr><td>PTPN2_Severity</td><td>Bacteria|Firmicutes|Clostridia|Clostridiales|Lachnospiraceae|Lachnospira</td><td>PTPN2_SeverityCT_Severe</td><td>0.01408766</td><td>41</td><td>40</td><td>0.11933725</td><td>0.35005593</td></tr>
<tr><td>PTPN2_Severity</td><td>Bacteria|Actinobacteria|Coriobacteriia|Coriobacteriales|Coriobacteriaceae|</td><td>PTPN2_SeverityCT_Severe</td><td>0.00512251</td><td>41</td><td>28</td><td>0.13000748</td><td>0.36401579</td></tr>
<tr><td>PTPN2_Severity</td><td>Bacteria|Firmicutes|Erysipelotrichi|Erysipelotrichales|Erysipelotrichaceae|Unclassified</td><td>PTPN2_SeverityCT_Severe</td><td>-0.0042268</td><td>41</td><td>27</td><td>0.13236938</td><td>0.36401579</td></tr>
<tr><td>PTPN2_Severity</td><td>Bacteria|Proteobacteria|Betaproteobacteria|Burkholderiales|Alcaligenaceae|Sutterella</td><td>PTPN2_SeverityCT_Severe</td><td>-0.0291047</td><td>41</td><td>40</td><td>0.14203575</td><td>0.37876199</td></tr>
<tr><td>PTPN2_Severity</td><td>Bacteria|Firmicutes|Clostridia|Clostridiales|Veillonellaceae|Dialister</td><td>PTPN2_SeverityCT_Severe</td><td>-0.0073186</td><td>41</td><td>30</td><td>0.18046166</td><td>0.46707725</td></tr>
<tr><td>PTPN2_Severity</td><td>Bacteria|Firmicutes|Clostridia|Clostridiales|Christensenellaceae|</td><td>PTPN2_SeverityCT_Severe</td><td>0.01030625</td><td>41</td><td>28</td><td>0.20331783</td><td>0.48750051</td></tr>
<tr><td>PTPN2_Severity</td><td>Bacteria|Actinobacteria|Actinobacteria|Actinomycetales|Actinomycetaceae|Actinomyces</td><td>PTPN2_SeverityCT_Severe</td><td>-0.0027887</td><td>41</td><td>27</td><td>0.20805141</td><td>0.48750051</td></tr>
<tr><td>PTPN2_Severity</td><td>Bacteria|Actinobacteria|Actinobacteria|Actinomycetales|Micrococcaceae|Rothia</td><td>PTPN2_SeverityCT_Severe</td><td>0.00321233</td><td>41</td><td>17</td><td>0.22148009</td><td>0.48750051</td></tr>
<tr><td>PTPN2_Severity</td><td>Bacteria|Proteobacteria|Gammaproteobacteria|Pseudomonadales|Pseudomonadaceae|Pseudomonas</td><td>PTPN2_SeverityCT_Severe</td><td>-0.0050163</td><td>41</td><td>24</td><td>0.22375861</td><td>0.48750051</td></tr>
<tr><td>PTPN2_Severity</td><td>Bacteria|Firmicutes|Clostridia|Clostridiales|[Mogibacteriaceae]|</td><td>PTPN2_SeverityCT_Severe</td><td>-0.0031837</td><td>41</td><td>23</td><td>0.23097395</td><td>0.48750051</td></tr>
<tr><td>PTPN2_Severity</td><td>Bacteria|Proteobacteria|Alphaproteobacteria|RF32|</td><td>PTPN2_SeverityCT_Severe</td><td>-0.0166816</td><td>41</td><td>31</td><td>0.23143479</td><td>0.48750051</td></tr>
<tr><td>PTPN2_Severity</td><td>Bacteria|Firmicutes|Clostridia|Clostridiales|Lachnospiraceae|Dorea</td><td>PTPN2_SeverityCT_Severe</td><td>0.00432884</td><td>41</td><td>40</td><td>0.23458339</td><td>0.48750051</td></tr>
<tr><td>PTPN2_Severity</td><td>Bacteria|Firmicutes|Bacilli|Bacillales|Staphylococcaceae|Staphylococcus</td><td>PTPN2_SeverityCT_Severe</td><td>-0.0031529</td><td>41</td><td>25</td><td>0.23564449</td><td>0.48750051</td></tr>
<tr><td>PTPN2_Severity</td><td>Bacteria|Bacteroidetes|Bacteroidia|Bacteroidales|[Odoribacteraceae]|Butyricimonas</td><td>PTPN2_SeverityCT_Severe</td><td>0.0087425</td><td>41</td><td>26</td><td>0.23821048</td><td>0.48750051</td></tr>
<tr><td>PTPN2_Severity</td><td>Bacteria|Firmicutes|Clostridia|Clostridiales|Ruminococcaceae|Faecalibacterium</td><td>PTPN2_SeverityCT_Severe</td><td>-0.0436721</td><td>41</td><td>41</td><td>0.24645431</td><td>0.49290863</td></tr>
<tr><td>PTPN2_Severity</td><td>Bacteria|Firmicutes|Clostridia|Clostridiales|Unclassified|Unclassified</td><td>PTPN2_SeverityCT_Severe</td><td>0.01320007</td><td>41</td><td>41</td><td>0.27689601</td><td>0.53134066</td></tr>
<tr><td>PTPN2_Severity</td><td>Bacteria|Firmicutes|Erysipelotrichi|Erysipelotrichales|Erysipelotrichaceae|Coprobacillus</td><td>PTPN2_SeverityCT_Severe</td><td>0.00269361</td><td>41</td><td>21</td><td>0.27774625</td><td>0.53134066</td></tr>
<tr><td>PTPN2_Severity</td><td>Bacteria|Firmicutes|Clostridia|Clostridiales|Clostridiaceae|SMB53</td><td>PTPN2_SeverityCT_Severe</td><td>-0.0013032</td><td>41</td><td>16</td><td>0.29684908</td><td>0.55520647</td></tr>
<tr><td>PTPN2_Severity</td><td>Bacteria|Firmicutes|Clostridia|Clostridiales|Veillonellaceae|Phascolarctobacterium</td><td>PTPN2_SeverityCT_Severe</td><td>0.01746121</td><td>41</td><td>31</td><td>0.3071892</td><td>0.55520647</td></tr>
<tr><td>PTPN2_Severity</td><td>Bacteria|Firmicutes|Clostridia|Clostridiales|Ruminococcaceae|Ruminococcus</td><td>PTPN2_SeverityCT_Severe</td><td>0.01705586</td><td>41</td><td>41</td><td>0.30914906</td><td>0.55520647</td></tr>
<tr><td>PTPN2_Severity</td><td>Bacteria|Bacteroidetes|Bacteroidia|Bacteroidales|</td><td>PTPN2_SeverityCT_Severe</td><td>0.00298034</td><td>41</td><td>19</td><td>0.36241347</td><td>0.63784771</td></tr>
<tr><td>PTPN2_Severity</td><td>Bacteria|Cyanobacteria|Chloroplast|Streptophyta|</td><td>PTPN2_SeverityCT_Severe</td><td>-0.0021339</td><td>41</td><td>13</td><td>0.41546063</td><td>0.71687325</td></tr>
<tr><td>PTPN2_Severity</td><td>Bacteria|Proteobacteria|Deltaproteobacteria|Desulfovibrionales|Desulfovibrionaceae|</td><td>PTPN2_SeverityCT_Severe</td><td>-0.0016995</td><td>41</td><td>13</td><td>0.44502058</td><td>0.74175639</td></tr>
<tr><td>PTPN2_Severity</td><td>Bacteria|Actinobacteria|Coriobacteriia|Coriobacteriales|Coriobacteriaceae|Adlercreutzia</td><td>PTPN2_SeverityCT_Severe</td><td>0.00157332</td><td>41</td><td>19</td><td>0.44820927</td><td>0.74175639</td></tr>
<tr><td>PTPN2_Severity</td><td>Bacteria|Firmicutes|Clostridia|Clostridiales|[Tissierellaceae]|Anaerococcus</td><td>PTPN2_SeverityCT_Severe</td><td>-0.0011848</td><td>41</td><td>13</td><td>0.45516869</td><td>0.74175639</td></tr>
<tr><td>PTPN2_Severity</td><td>Bacteria|Firmicutes|Clostridia|Clostridiales|</td><td>PTPN2_SeverityCT_Severe</td><td>0.01431834</td><td>41</td><td>41</td><td>0.47285605</td><td>0.75656969</td></tr>
<tr><td>PTPN2_Severity</td><td>Bacteria|Firmicutes|Bacilli|Turicibacterales|Turicibacteraceae|Turicibacter</td><td>PTPN2_SeverityCT_Severe</td><td>0.00161126</td><td>41</td><td>22</td><td>0.48443759</td><td>0.76125907</td></tr>
<tr><td>PTPN2_Severity</td><td>Bacteria|Firmicutes|Clostridia|Clostridiales|Lachnospiraceae|[Ruminococcus_</td><td>PTPN2_SeverityCT_Severe</td><td>0.00563086</td><td>41</td><td>41</td><td>0.5016084</td><td>0.76665001</td></tr>
<tr><td>PTPN2_Severity</td><td>Bacteria|Firmicutes|Clostridia|Clostridiales|Peptostreptococcaceae|Peptostreptococcus</td><td>PTPN2_SeverityCT_Severe</td><td>-0.0013284</td><td>41</td><td>13</td><td>0.50529205</td><td>0.76665001</td></tr>
<tr><td>PTPN2_Severity</td><td>Bacteria|Proteobacteria|Gammaproteobacteria|Enterobacteriales|Enterobacteriaceae|Gluconacetobacter</td><td>PTPN2_SeverityCT_Severe</td><td>-0.0015708</td><td>41</td><td>14</td><td>0.52197497</td><td>0.76832618</td></tr>
<tr><td>PTPN2_Severity</td><td>Bacteria|Bacteroidetes|Bacteroidia|Bacteroidales|Porphyromonadaceae|Parabacteroides</td><td>PTPN2_SeverityCT_Severe</td><td>0.01044211</td><td>41</td><td>41</td><td>0.52825344</td><td>0.76832618</td></tr>
<tr><td>PTPN2_Severity</td><td>Bacteria|Firmicutes|Clostridia|Clostridiales|Veillonellaceae|Acidaminococcus</td><td>PTPN2_SeverityCT_Severe</td><td>0.00081213</td><td>41</td><td>13</td><td>0.53258974</td><td>0.76832618</td></tr>
<tr><td>PTPN2_Severity</td><td>Bacteria|Firmicutes|Clostridia|Clostridiales|Peptostreptococcaceae|</td><td>PTPN2_SeverityCT_Severe</td><td>0.00108405</td><td>41</td><td>22</td><td>0.55292717</td><td>0.78479986</td></tr>
<tr><td>PTPN2_Severity</td><td>Bacteria|Firmicutes|Clostridia|Clostridiales|Veillonellaceae|Veillonella</td><td>PTPN2_SeverityCT_Severe</td><td>-0.0027355</td><td>41</td><td>30</td><td>0.5706329</td><td>0.79707453</td></tr>
<tr><td>PTPN2_Severity</td><td>Bacteria|Proteobacteria|Gammaproteobacteria|Enterobacteriales|Enterobacteriaceae|</td><td>PTPN2_SeverityCT_Severe</td><td>-0.0066117</td><td>41</td><td>38</td><td>0.61448307</td><td>0.84491422</td></tr>
<tr><td>PTPN2_Severity</td><td>Bacteria|Proteobacteria|Alphaproteobacteria|Rhizobiales|Bradyrhizobiaceae|Unclassified</td><td>PTPN2_SeverityCT_Severe</td><td>-0.0006459</td><td>41</td><td>15</td><td>0.64212357</td><td>0.86867433</td></tr>
<tr><td>PTPN2_Severity</td><td>Bacteria|Firmicutes|Clostridia|Clostridiales|Lachnospiraceae|Anaerostipes</td><td>PTPN2_SeverityCT_Severe</td><td>-0.0016107</td><td>41</td><td>22</td><td>0.65618159</td><td>0.86867433</td></tr>
<tr><td>PTPN2_Severity</td><td>Bacteria|Firmicutes|Bacilli|Lactobacillales|Streptococcaceae|Unclassified</td><td>PTPN2_SeverityCT_Severe</td><td>-0.0008664</td><td>41</td><td>18</td><td>0.66934785</td><td>0.86867433</td></tr>
<tr><td>PTPN2_Severity</td><td>Bacteria|Bacteroidetes|Bacteroidia|Bacteroidales|[Odoribacteraceae]|Odoribacter</td><td>PTPN2_SeverityCT_Severe</td><td>0.00546313</td><td>41</td><td>37</td><td>0.68290102</td><td>0.86867433</td></tr>
<tr><td>PTPN2_Severity</td><td>Bacteria|Firmicutes|Clostridia|Clostridiales|Lachnospiraceae|Epulopiscium</td><td>PTPN2_SeverityCT_Severe</td><td>-0.0008813</td><td>41</td><td>24</td><td>0.68781491</td><td>0.86867433</td></tr>
<tr><td>PTPN2_Severity</td><td>Bacteria|Proteobacteria|Deltaproteobacteria|Desulfovibrionales|Desulfovibrionaceae|Bilophila</td><td>PTPN2_SeverityCT_Severe</td><td>0.00298681</td><td>41</td><td>24</td><td>0.69691099</td><td>0.86867433</td></tr>
<tr><td>PTPN2_Severity</td><td>Bacteria|Proteobacteria|Gammaproteobacteria|Pasteurellales|Pasteurellaceae|Haemophilus</td><td>PTPN2_SeverityCT_Severe</td><td>-0.002762</td><td>41</td><td>39</td><td>0.72491404</td><td>0.86867433</td></tr>
<tr><td>PTPN2_Severity</td><td>Bacteria|Proteobacteria|Gammaproteobacteria|Pseudomonadales|Moraxellaceae|Acinetobacter</td><td>PTPN2_SeverityCT_Severe</td><td>-0.0006583</td><td>41</td><td>18</td><td>0.72670899</td><td>0.86867433</td></tr>
<tr><td>PTPN2_Severity</td><td>Bacteria|Actinobacteria|Coriobacteriia|Coriobacteriales|Coriobacteriaceae|Collinsella</td><td>PTPN2_SeverityCT_Severe</td><td>0.00173072</td><td>41</td><td>37</td><td>0.72799778</td><td>0.86867433</td></tr>
<tr><td>PTPN2_Severity</td><td>Bacteria|Actinobacteria|Coriobacteriia|Coriobacteriales|Coriobacteriaceae|Eggerthella</td><td>PTPN2_SeverityCT_Severe</td><td>0.00051552</td><td>41</td><td>17</td><td>0.73047614</td><td>0.86867433</td></tr>
<tr><td>PTPN2_Severity</td><td>Bacteria|Bacteroidetes|Bacteroidia|Bacteroidales|[Paraprevotellaceae]|Paraprevotella</td><td>PTPN2_SeverityCT_Severe</td><td>0.00269291</td><td>41</td><td>15</td><td>0.75956052</td><td>0.88736556</td></tr>
<tr><td>PTPN2_Severity</td><td>Bacteria|Firmicutes|Erysipelotrichi|Erysipelotrichales|Erysipelotrichaceae|[Eubacterium_</td><td>PTPN2_SeverityCT_Severe</td><td>-0.0043585</td><td>41</td><td>38</td><td>0.76636117</td><td>0.88736556</td></tr>
<tr><td>PTPN2_Severity</td><td>Bacteria|Firmicutes|Bacilli|Gemellales|Gemellaceae|Unclassified</td><td>PTPN2_SeverityCT_Severe</td><td>0.00053245</td><td>41</td><td>21</td><td>0.79700608</td><td>0.90217368</td></tr>
<tr><td>PTPN2_Severity</td><td>Bacteria|Firmicutes|Bacilli|Lactobacillales|Streptococcaceae|Streptococcus</td><td>PTPN2_SeverityCT_Severe</td><td>0.00192866</td><td>41</td><td>40</td><td>0.80548489</td><td>0.90217368</td></tr>
<tr><td>PTPN2_Severity</td><td>Bacteria|Proteobacteria|Gammaproteobacteria|Pasteurellales|Pasteurellaceae|Actinobacillus</td><td>PTPN2_SeverityCT_Severe</td><td>0.00055807</td><td>41</td><td>20</td><td>0.80990592</td><td>0.90217368</td></tr>
<tr><td>PTPN2_Severity</td><td>Bacteria|Firmicutes|Bacilli|Bacillales|Unclassified|Unclassified</td><td>PTPN2_SeverityCT_Severe</td><td>0.00049375</td><td>41</td><td>27</td><td>0.84806966</td><td>0.93287662</td></tr>
<tr><td>PTPN2_Severity</td><td>Bacteria|Firmicutes|Clostridia|Clostridiales|Lachnospiraceae|</td><td>PTPN2_SeverityCT_Severe</td><td>0.0062678</td><td>41</td><td>41</td><td>0.8884293</td><td>0.96487475</td></tr>
<tr><td>PTPN2_Severity</td><td>Bacteria|Bacteroidetes|Bacteroidia|Bacteroidales|Prevotellaceae|Prevotella</td><td>PTPN2_SeverityCT_Severe</td><td>-0.0005693</td><td>41</td><td>40</td><td>0.90786814</td><td>0.96487475</td></tr>
<tr><td>PTPN2_Severity</td><td>Bacteria|Verrucomicrobia|Verrucomicrobiae|Verrucomicrobiales|Verrucomicrobiaceae|Akkermansia</td><td>PTPN2_SeverityCT_Severe</td><td>0.00032679</td><td>41</td><td>32</td><td>0.91005232</td><td>0.96487475</td></tr>
<tr><td>PTPN2_Severity</td><td>Bacteria|Proteobacteria|Gammaproteobacteria|Enterobacteriales|Enterobacteriaceae|Unclassified</td><td>PTPN2_SeverityCT_Severe</td><td>0.00012034</td><td>41</td><td>15</td><td>0.93655718</td><td>0.97661063</td></tr>
<tr><td>PTPN2_Severity</td><td>Bacteria|Firmicutes|Erysipelotrichi|Erysipelotrichales|Erysipelotrichaceae|</td><td>PTPN2_SeverityCT_Severe</td><td>0.00071249</td><td>41</td><td>40</td><td>0.94331709</td><td>0.97661063</td></tr>
<tr><td>PTPN2_Severity</td><td>Bacteria|Firmicutes|Clostridia|Clostridiales|Clostridiaceae|</td><td>PTPN2_SeverityCT_Severe</td><td>-0.0002266</td><td>41</td><td>34</td><td>0.95569426</td><td>0.97791971</td></tr>
<tr><td>PTPN2_Severity</td><td>Bacteria|Firmicutes|Clostridia|Clostridiales|Clostridiaceae|Unclassified</td><td>PTPN2_SeverityCT_Severe</td><td>-0.0001223</td><td>41</td><td>31</td><td>0.9705227</td><td>0.98080628</td></tr>
<tr><td>PTPN2_Severity</td><td>Bacteria|Firmicutes|Bacilli|Bacillales|Planococcaceae|Rummeliibacillus</td><td>PTPN2_SeverityCT_Severe</td><td>-3.30E-05</td><td>41</td><td>13</td><td>0.98080628</td><td>0.98080628</td></tr>
</table>
